# *Plasmodium vivax* Merozoite Surface Protein-3 (PvMSP3): Expression of an 11 Member Multigene Family in Blood-Stage Parasites

**DOI:** 10.1371/journal.pone.0063888

**Published:** 2013-05-23

**Authors:** Jianlin Jiang, John W. Barnwell, Esmeralda V. S. Meyer, Mary R. Galinski

**Affiliations:** 1 Emory Vaccine Center, Yerkes National Primate Research Center, Emory University, Atlanta, Georgia, United States of America; 2 Malaria Branch, Division of Parasitic Diseases and Malaria, Centers for Disease Control and Prevention, Atlanta, Georgia, United States of America; 3 Department of Medicine, Division of Infectious Diseases, Emory University School of Medicine and Emory Vaccine Center, Yerkes National Primate Research Center, Emory University, Atlanta, Georgia, United States of America; Universidade Federal de Minas Gerais, Brazil

## Abstract

**Background:**

Three members of the *Plasmodium vivax* merozoite surface protein-3 (PvMSP3) family (PvMSP3-α, PvMSP3-β and PvMSP3-γ) were initially characterized and later shown to be part of a larger highly diverse family, encoded by a cluster of genes arranged head-to-tail in chromosome 10. PvMSP3-α and PvMSP3-β have become genetic markers in epidemiological studies, and are being evaluated as vaccine candidates. This research investigates the gene and protein expression of the entire family and pertinent implications.

**Methodology/Principal Findings:**

A 60 kb multigene locus from chromosome 10 in *P. vivax* (Salvador 1 strain) was studied to classify the number of *pvmsp*3 genes present, and compare their transcription, translation and protein localization patterns during blood-stage development. Eleven *pvmsp*3 paralogs encode an N-terminal NLRNG signature motif, a central domain containing repeated variable heptad sequences, and conserved hydrophilic C-terminal features. One additional ORF in the locus lacks these features and was excluded as a member of the family. Transcripts representing all eleven *pvmsp*3 genes were detected in trophozoite- and schizont-stage RNA. Quantitative immunoblots using schizont-stage extracts and antibodies specific for each PvMSP3 protein demonstrated that all but PvMSP3.11 could be detected. Homologs were also detected by immunoblot in the closely related simian species, *P. cynomolgi* and *P. knowlesi*. Immunofluorescence assays confirmed that eight of the PvMSP3s are present in mature schizonts. Uniquely, PvMSP3.7 was expressed exclusively at the apical end of merozoites.

**Conclusion/Significance:**

Specific proteins were detected representing the expression of 10 out of 11 genes confirmed as members of the *pvmsp*3 family. Eight PvMSP3s were visualized surrounding merozoites. In contrast, PvMSP3.7 was detected at the apical end of the merozoites. *Pvmsp*3.11 transcripts were present, though no corresponding protein was detected. PvMSP3 functions remain unknown. The ten expressed PvMSP3s are predicted to have unique and complementary functions in merozoite biology.

## Introduction

*Plasmodium vivax* causes extensive morbidity in over 95 countries, accounting for 2.85 billion people exposed to transmission, and the potential to cause severe disease and sometimes death [1], [2]. There is an urgency to reduce and ultimately eliminate these infections, with emphasis on the prevention and treatment of this species’ active and quiescent liver-stage forms, as well as blood-stage forms that are the cause of disease manifestation and transmission [3]–[5].

The merozoite stage of the parasite’s life cycle represents one possible target of intervention [6]. *Plasmodium* merozoites are single cell Apicomplexan parasitic organisms that are ripe with proteins that are critical for the successful invasion and propagation of the parasite within erythrocytes. The merozoites have a highly structured protein surface coat comprised of ten or more unique proteins. In electron transmission microscopy images (shown for *P. vivax*) the merozoite surface has a spiked appearance, suggesting an organized presentation of the proteins at the surface [7]. The merozoite’s apical pole contains rhoptry, microneme and other organelles with proteins known to be involved in the attachment to red blood cells, invasion and host cell modification [8]. Within minutes after being released from infected erythrocytes, each merozoite progeny must properly attach to and invade a new host red blood cell. A cascade of molecular adhesion and enzymatic processes occur, with various points for possible intervention, as the merozoite’s apical pole initiates the entry process and the parasite becomes enveloped and secluded within the selected host cell. Both the surface molecules and proteins that become localized at the apical pole are important for the successful invasion of erythrocytes by merozoites. The surface coat and organellar proteins are therefore of high interest as possible malaria vaccine candidates or drug targets, for *P. vivax* and other species. However, the full set and specific roles of the composite proteins are still largely under defined [9]–[12].

The MSP3 family of *P. vivax* was originally characterized based on the identification of three related genes and encoded proteins: *pvmsp*3-α, *pvmsp*3-β, and *pvmsp*3-γ [13], [14]. Each of these *pvmsp*3 genes encodes a protein with a predominant central alanine-rich domain containing heptad repeats predicted to form α-helical secondary and coiled-coil tertiary structures [15]. PvMSP3-α and PvMSP3-β were shown to be present at the surface of merozoites, within schizont-infected red blood cells (RBCs), as well as free merozoites, despite their lack of a transmembrane domain or GPI-lipid modification site [13], [14]. The PvMSP3 members were therefore predicted to be associated with other merozoite surface molecules, possibly through protein-protein interactions involving the coiled-coil domains [13], [14], similarly as suggested for the initially described *P. falciparum* MSP3 [16], [17]. The coiled-coil structure in PfMSP3.1 has been shown experimentally by ‘H NMR spectroscopy with the analysis of synthesized peptide, whereby the α-helices were found to contain 38 amino acids [18]. The α-helical secondary structures present in recombinant PfMSP3.1 [19], rPvMSP3-α (PvMSP3.10) and rPvMSP3-β (PvMSP3.3) [20] were also confirmed by far-UV CD spectroscopy. The central coiled-coil domains are highly polymorphic, while the flanking N- and C-terminal regions are relatively more conserved, but still polymorphic. As shown for *pvmsp*3*-*α and *pvmsp*3*-*β, the allelic polymorphism observed in the central domain can include many point mutations as well as large insertions and deletions [21], [22]. Diversity studies with isolates from around the world continue to show extensive polymorphism in these genes and the encoded proteins, and the *pvmsp*3 alleles have therefore become highly regarded as genetic tools to distinguish different parasite isolates and study population dynamics [23]. They have also been regarded as potential vaccine candidates, following in the path of PfMSP3 [24], [25]. Immune response studies carried out to date in Brazil assessing naturally acquired immunity to PvMSP3-α show the presence of broadly recognized B cell epitopes from the central region of PvMSP3α, IgG1 and IgG3 antibody subclasses associated with increased exposure to the parasite, and the association of HLA types with such responses [26], [27]. Others have reported a predominance of IgG1 and IgG2, and an association with anemia [28].

With the discovery of three members of the *P. vivax* MSP3 family (PvMSP3α, β, and γ proteins), prior to the genome era, an unusual defining characteristic signature motif with the consensus sequence NLRNG was noted immediately after the signal peptide and shown to be conserved in homolog proteins present in *P. knowlesi* and *P. falciparum* [13]. This signature motif and the availability of *Plasmodium* genome sequences facilitated the recognition of additional family members in the context of a multigene locus of *msp*3 genes in *P. vivax* and *P. falciparum* the [29]–[32].

In the current study, we aimed to investigate the structure and expression of each of the putative *P. vivax msp*3 genes present in a 60 kb multigene locus in chromosome 10 [30]. Whether all or a subset of these predicted *msp*3 ORFs are transcribed and translated in blood-stage parasites was unknown, and this information is relevant for understanding the parasite’s biology and the potential of family members as vaccine or drug targets. We have succeeded in further characterizing and defining the expression of this gene family, and we also report the discovery of a novel apically located protein that may be functionally important for the invasion of RBCs.

## Results

### Definition of the pvmsp3 Multigene Locus with Eleven Putative PvMSP3 Members Encoded in Tandem

**Figure 1:**
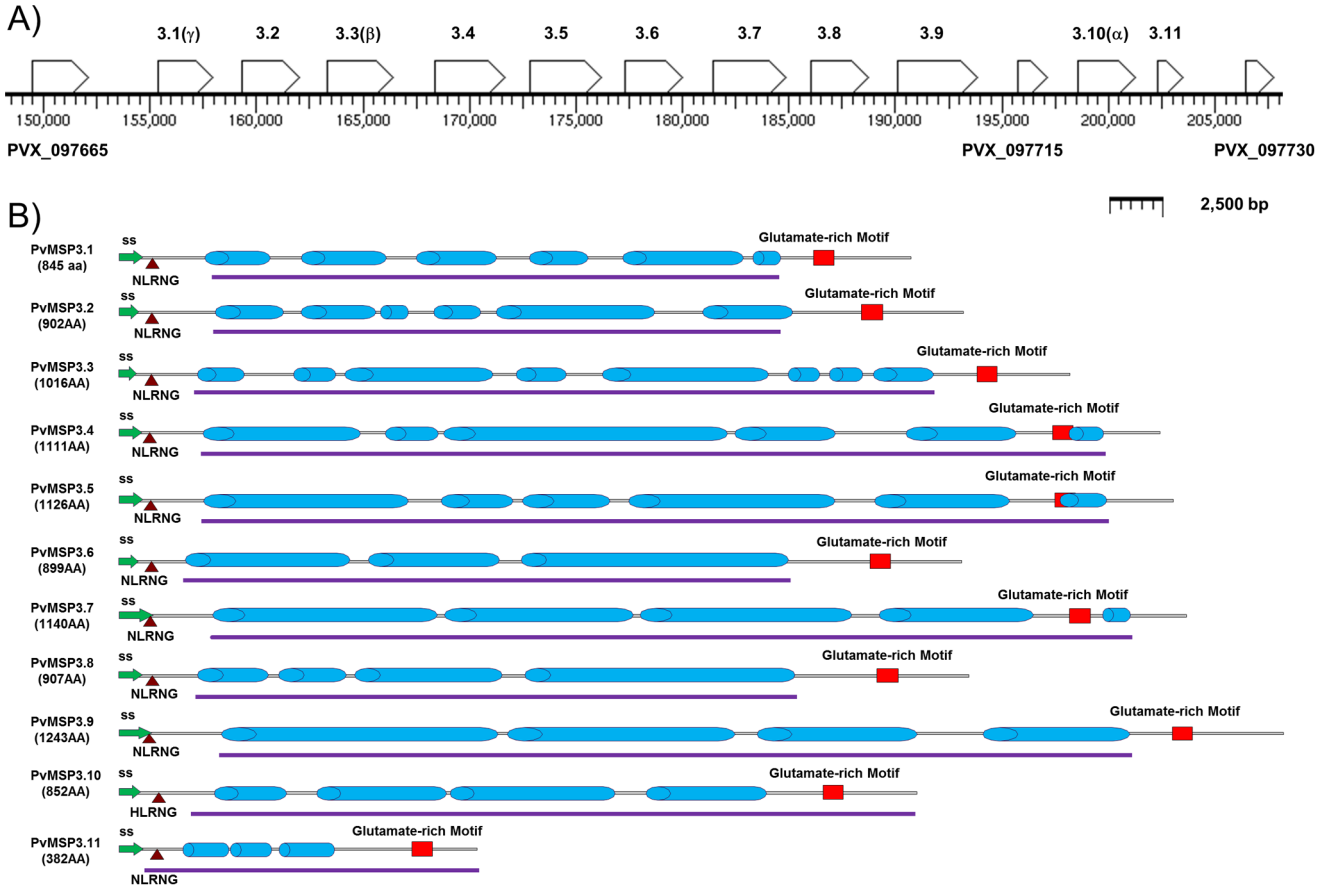
Schematic depicting the eleven members of the *pvmsp*3 gene family in chromosome 10 and characteristic features of all PvMSP3 family members. 1A. *Pvmsp*3 gene family members are arranged in tandem in chromosome 10, and flanked by unrelated loci (PVX_097665 and PVX_097730). The eleven *pvmsp*3 genes are represented as open boxes and labeled as *pvmsp*3.1 through *pvmsp*3.11. The gene IDS are: PVX_097670, PVX_097675, PVX_097680, PVX_097685, PVX_097690, PVX_097695, PVX_097700, PVX_097705, PVX_097710, PVX_097720, PVX_097725, respectively. The originally described *pvmsp*3 family members (α, β and γ) are also marked as such. One ORF, PVX_097715, does not encode characteristic features of PvMSP3 proteins (Fig. 2) and has thus been excluded as a member of the *pvmsp*3 gene family. **1B**. Each PvMSP-3 is depicted with the protein’s size in amino acids noted. The predominant centrally located coiled-coil structures of each protein are depicted by blue coiled cylinders. Other characteristic features include a conserved NLRNG motif (triangle) near the N-terminus and a glutamate-rich motif (red rectangles) near the C-terminus. Putative signal peptides (green) are marked by arrows at the N-termini and purple lines below each protein schematic underscores the regions expressed as recombinant proteins. The calculated molecular weights of these recombinant proteins are noted in Table 1.

The *P. vivax* genome database shows that the *pvmsp*3-α, *pvmsp*3-β and *pvmsp*3-γ genes encoding the originally identified members of the MSP3 family [13], [14] are located in chromosome 10 within a segment of DNA spanning close to 60 kb (www.plasmodb.org). Our analyses of this region and the surrounding DNA has resulted in eight additional open reading frames (ORF) within the ∼60 kb segment being classified as members of the *pvmsp*3 family and the complete set of eleven *msp*3 genes being annotated as *pvmsp*3.1 through *pvmsp*3.11 [30] (Fig. 1A, Table 1). Based on their position in the series, the *pvmsp*3-α, *pvmsp*3-β, and *pvmsp*3-γ genes have been renamed *pvmsp*3.10, *pvmsp*3.3 and *pvmsp*3.1, respectively. Each gene is contained in a single exon, without intervening sequences, and they are positioned head-to-tail within the multigene locus. The ATG start codon of each member of the *pvmsp*3 family is preceded by an adenine at position -3, forming the expected consensus start sequence described in *Plasmodium* [33].

**Table 1:** Biochemical characteristics of PvMSP3 proteins.

Name	PvMSP3.1(γ)	PvMSP3.2	PvMSP3.3(β)	PvMSP3.4	PvMSP3.5	PvMSP3.6	PvMSP3.7	PvMSP3.8	PvMSP3.9	PvMSP3.10(α)	PvMSP3.11
	PVX_097670	PVX_097675	PVX_097680	PVX_097685	PVX_097690	PVX_097695	PVX_097700	PVX_097705	PVX_097710	PVX_097720	PVX_097725
**Size (bp)**	2535	2706	3048	3333	3378	2697	3420	2721	3729	2556	1146
**Deduced Protein M.W. (kDa)**	91.5	97.4	109.7	117.5	120.8	96	122.1	97	134	90.6	41.5
**Recombinant protein M.W. (kDa)**	64.1	76.0	84.1	101.2	102.8	67.9	104.2	67.5	103.1	81.7	38.2
**Ala (%)**	19.76	19.29	18.8	23.31	21.67	21.02	23.33	21.28	22.2	22.18	15.97
**Glu (%)**	16.21	16.3	18.31	15.3	15.99	15.13	16.84	15.66	18.1	15.61	20.42
**Lys (%)**	16.21	15.3	16.54	16.11	15.9	15.24	15.7	16.1	15.93	14.67	10.21
**pI**	4.96	4.96	4.94	4.92	4.88	4.96	4.88	5.00	4.95	4.96	4.17
**C-C region (%)**	66.30%	69.30%	71.20%	75.30%	76.30%	84.20%	80.00%	82.40%	80.50%	75.40%	66.10%

pI = Isoelectric Point; C-C = Coiled-Coil.

**Figure 2:**
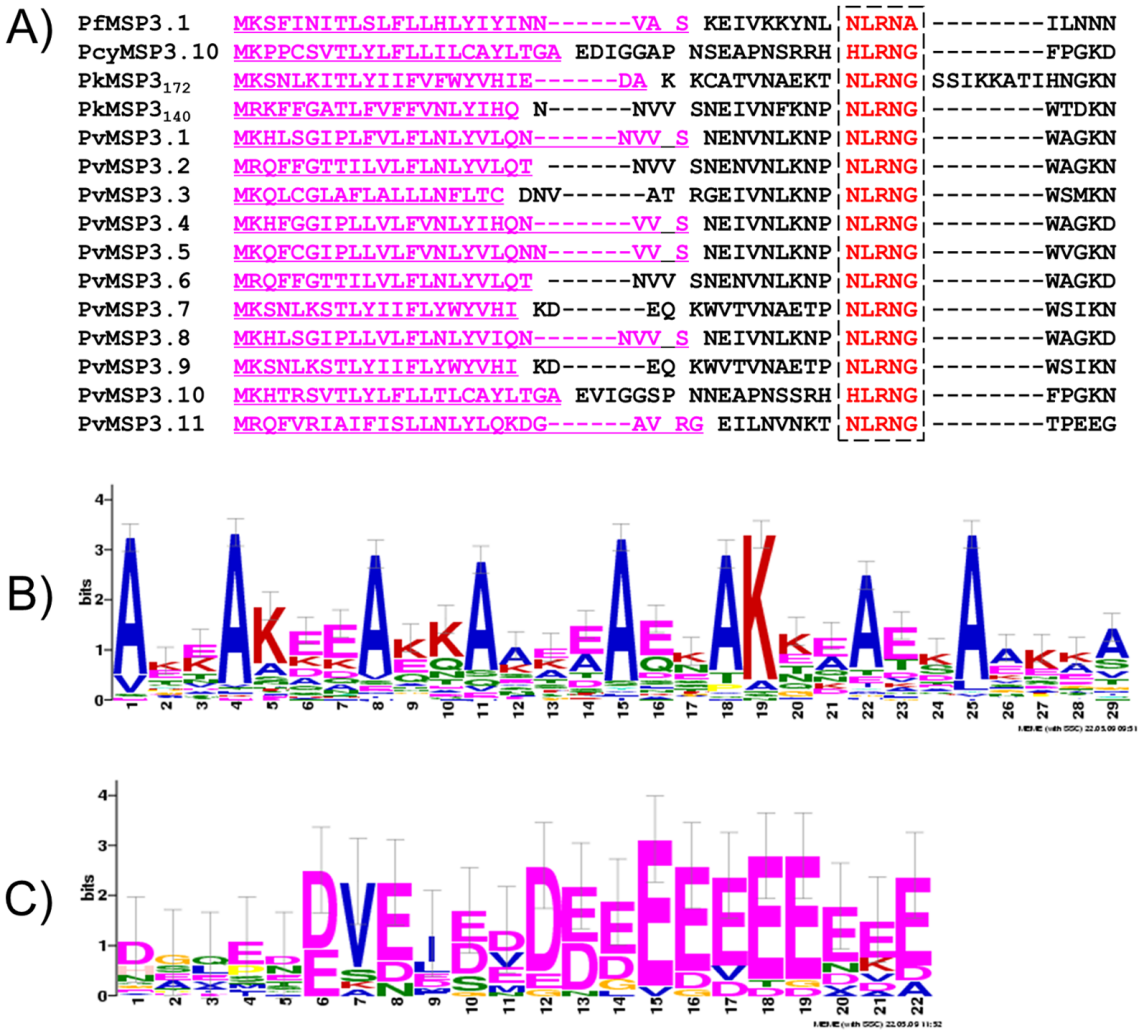
Three motifs are common to all known *Plasmodium* MSP3 proteins. Putative protein sequences used to generate these analyses were obtained from the gene IDs presented in Table S2. **2A**. The conserved NLRNG motif, common in all MSP3 proteins is evident at the N-termini, 8–16 amino acids downstream of the putative signal peptide cleavage site (underlined). **2B**. One typical tandemly-repeated heptad motif with the consensus sequence AXXAXXX to form α-helical coiled-coil structures were found at all MSP3 proteins including PfMSP3, PkMSP3, and PcyMSP3 (details in Fig. S2). **2C**. The glutamate-rich motif exists in all MSP3 C-termini (details in Fig. S3), this hydrophilic domain is characterized by a high content of acidic amino acids in clusters, particularly glutamates.

Three characteristic protein features have been confirmed for the proteins encoded by all eleven members of the *pvmsp*3 family: an NLRNG motif immediately following the putative signal peptide cleavage site, a predominant central alanine-rich region with predicted coiled-coil domains, and a C-terminal motif containing a glutamate-rich domain (Fig. 1B, 2, S1, S2 and S3).

To arrive at this conclusion, the MEME (Multiple Em for Motif Elicitation) algorithm was used to search for significantly overrepresented amino acid residues in each predicted PvMSP3 family member. Our MEME analysis also included the full-length sequences of the following MSP3 orthologs from *P. falciparum*, *P. knowlesi* and *P. cynomolgi*, respectively: PfMSP3.1 (FC27 and CSL2 strains), PkMSP3_140_, PkMSP3_172_, and PcyMSP3.10 (Berok strain) (Fig. 2, sequence accession numbers in Table S2). It is of special interest that the NLRNG motif is present in each deduced MSP3 sequence, despite otherwise quite divergent MSP3 sequences in all of these species, whether closely (*P. cynomolgi* and *P. knowlesi*) or distantly (*P. falciparum*) related to *P. vivax* [34].

Using the Multicoil algorithm (http://groups.csail.mit.edu/cb/multicoil/cgi-bin/multicoil.cgi), the central alanine-rich region of all predicted PvMSP3s scores significantly above the cut-off value for the formation of coiled-coils. The central domain spans the majority (66.1%–84.2%) of the total amino acid sequences, and this domain differs in size for each member of the family (Table 1, Fig. 1B, S1 and S2). Though the alanine residues are often in positions **a** and **d** of the tandemly repeated heptad AxxAxxx of other MSP3 proteins, we found that the predicted heptad repeat in the PvMSP3 family is better described as having any hydrophobic residue, such as Valine, Isoleucine, Serine or Alanine in the **a** and **d** positions (Fig. 2B, S1 and S2).

A complete protein sequence analysis revealed that Ala, Glu, and Lys account for more than 50% of the amino acid composition of the entire PvMSP3 family, although there is low amino acid sequence identity between family members (Table 1). They also have different calculated molecular weights ranging from 41.1 kDa to 134 kDa. The isoelectric point (pI) of 10 of the 11 predicted proteins is close to 5.0, with the one exception being the smallest predicted member, PvMSP3.11, with a pI of 4.2 (Table 1). It is worth noting that in further experimental studies (below) we did not detect any protein expressed from this gene. Thus, the predicted molecular weights of the PvMSP3s that were found to be expressed range from 90.6 kDa to 134 kDa, although each of them consistently migrates significantly higher by SDS-PAGE (see below), as reported earlier for the PvMSP3α and β family members [13], [14].

Global pairwise alignment of the putative PvMSP3 sequences results in similarity scores well below 50%; however, identity scores are much higher between PvMSP3.6 and PvMSP3.8 (81.8%), PvMSP3.4 and PvMSP3.5 (72.6%), and PvMSP3.7 and PvMSP3.9 (63.2%) (Table 2). Alignment of the predicted amino acids of these proteins showed varying identity and similarity percentages, for example, PvMSP3.6 and PvMSP3.8 scored 88 and 82%, for similarity and identity, respectively (Table 2), suggesting a common ancestry and occurrence of gene duplications, compared to the highly polymorphic central domains.

**Table 2:** Amino acid similarities and identities between the different members of the PvMSP3 family.

%	PvMSP3.1 (γ)	PvMSP3.2	PvMSP3.3 (β)	PvMSP3.4	PvMSP3.5	PvMSP3.6	PvMSP3.7	PvMSP3.8	PvMSP3.9	PvMSP3.10 (α)	PvMSP3.11
PvMSP3.1 (γ)		**41**	**31.6**	**34**	**31.8**	**35.5**	**29.9**	**36.7**	**29.4**	**34.8**	**19.2**
PvMSP3.2	*56.8*		**31.4**	**32.7**	**31.8**	**37.6**	**28.5**	**36.8**	**27.8**	**33.3**	**18.5**
PvMSP3.3 (β)	*47.9*	*48.5*		**34.1**	**34.1**	**31.8**	**34**	**32.5**	**31.2**	**32**	**18.2**
PvMSP3.4	*45.4*	*46.7*	*52.3*		**72.6**	**33**	**44.8**	**33.5**	**41.8**	**29.9**	**16.6**
PvMSP3.5	*45.1*	*46.4*	*51.5*	*83.4*		**33.4**	**47**	**32.8**	**42.9**	**29.6**	**16.1**
PvMSP3.6	*54.5*	*55.3*	*50*	*47.9*	*48*		**29.5**	**81.8**	**29.3**	**40.1**	**19.2**
PvMSP3.7	*42.7*	*44*	*49.6*	*61.2*	*63.8*	*44.4*		**30**	**63.2**	**29.9**	**16.4**
PvMSP3.8	*55.9*	*55.5*	*50.5*	*49.4*	*48.1*	*87.8*	*45.7*		**29.2**	**39.9**	**20.5**
PvMSP3.9	*41.5*	*42.2*	*48.3*	*57.7*	*59*	*43.1*	*74.8*	*43.4*		**27.5**	**14.6**
PvMSP3.10 (α)	*54*	*51.2*	*47.6*	*43.7*	*43.3*	*59.4*	*43.2*	*58.2*	*40.8*		**19**
PvMSP3.11	*27.6*	*27.2*	*25.3*	*23*	*23.3*	*27.6*	*23.1*	*27.9*	*20.8*	*27.7*	

Similarities (*italics*) and identities (**bolded**) were generated with the MatGAT 2.0 program (http://www.bitincka.com/ledion/matgat/) and the BLOSUM50 scoring matrix.

The third characteristic motif of PvMSP3 members is a hydrophilic region in the C-terminus, which contains a high content of acidic amino acids in clusters, particularly glutamic acid and aspartic acid (Fig. 1B, 2C and S3), and lacks a C-terminal transmembrane motif, or any site for post-translational GPI modifications. Notably, all PvMSP3 members also lack a leucine-zipper, which is typical at the terminal end of MSP3 proteins in *P. falciparum* [16,17 31,35].

An additional ORF was identified between *pvmsp*3.9 and *pvmsp*3.10 with the gene ID: PVX_097715 (Fig. 1A). The putative protein sequence encoded by this ORF lacks the typical coiled-coil region, the N-terminal NLRNG motif and the C-terminal motif containing the glutamate-rich domain; therefore, it has not been included as a member of this family. Likewise, the 5′ (PVX_097665) and 3′ (PVX_097730) ORFs flanking the *pvmsp*3 cluster do not have characteristic features of *pvmsp*3 genes [13], [14]. These are annotated in PlasmoDB database as the 4*-diphosphocytidyl-*2c*-methyl-*D*-erythritol kinase* gene and a hypothetical gene, respectively.

### All Eleven pvmsp3 Gene Transcripts were Detected in *P. vivax* Blood-stage Parasites

**Figure 3:**
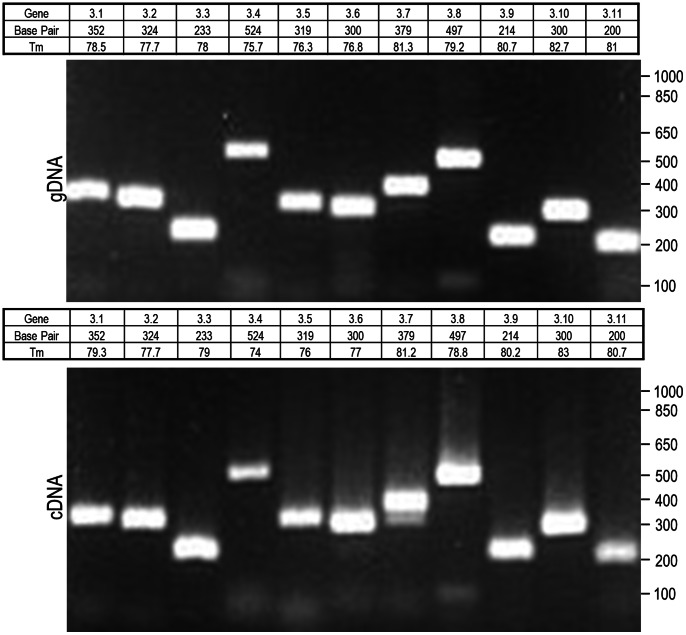
Comparison between gene and transcript amplification of each *pvmsp*3. Amplification products are shown after electrophoresis in 1.5% agarose gels. Top panel: PCR products from genomic DNA; bottom panel: RT-PCR products from cDNA.

We set out to determine whether all eleven putative *pvmsp*3 genes were transcribed in blood-stage infections, or only a subset. If each of these genes proved to be transcribed, it was also important to know when, during the life cycle of the parasite, these transcripts were being produced and their abundance. To achieve this aim, gene-specific primer sets based on the *P. vivax* Salvador I strain sequences (Table S1) were tested in PCR and RT-PCR experiments using *P. vivax* Salvador I strain gDNA and cDNA as templates, respectively. The primer pairs were strategically designed to generate products of different sizes to distinguish and unambiguously confirm the specific expression of individual gene family members. The cDNA was produced from total RNA representing predominantly *P. vivax* Salvador I strain schizonts from a *S. boliviensis* blood-stage infection. The expected product sizes for all eleven genes were obtained in all reactions performed with gDNA and cDNA (Fig. 3). The melting curve generated with SYBR Green after PCR and RT-PCR amplifications showed similar, sometimes identical, melting temperatures (Tm) between the two templates (Fig. 3). These primer pairs were also evaluated for their ability to amplify the individual *pvmsp*3 genes from *P. vivax* gDNA of the Belem strain. No amplicons were detected for *pvmsp*3.1, 3.2, 3.3, 3.5, and 3.6 from this strain, while amplicons were detected for the rest of the genes (data not shown). In these reactions, the retrieval of all *pvmsp*3 genes would not be expected, due to polymorphisms between the respective gene sequences represented by the oligonucleotide primer pairs, or possible differences in the number of *pvmsp*3 genes maintained in each strain.

**Figure 4:**
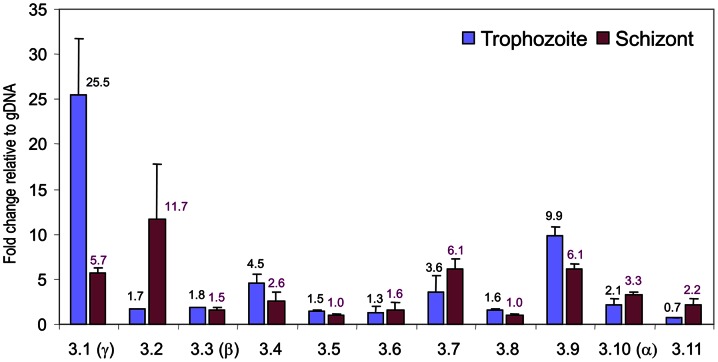
Stage-specific transcriptional level of the *pvmsp*3 gene family members evaluated by real-time PCR. The fold change of each *pvmsp*3 transcript is shown from trophozoite- and schizont-stage RNA samples relative to genomic DNA.

Next, we sought to determine whether each of the *pvmsp*3 transcripts from the *P. vivax* Salvador I strain was similarly expressed, or not. Real-time quantitative qRT-PCR assays were carried out with Taqman probes for specificity to quantify and compare the *pvmsp*3 transcript levels of each family member present in predominantly trophozoite or schizont total RNA samples, purified from *P. vivax* Salvador I strain infected RBCs generated from *S. boliviensis* blood-stage infections (Table S3 and Fig. 4). The real-time qRT-PCR results confirmed that all eleven *pvmsp*3 genes from the Salvador I strain of *P. vivax* were transcribed during the blood-stage cycle, but at different levels. The detected transcript levels of the different members of the gene family showed between 0.7- to 25.5-fold changes relative to the gDNA calibrator sample. The overall transcription level of the *pvmsp*3 genes was below a 6-fold change relative to gDNA in both the trophozoite and schizont stages. However, in the trophozoite-stage sample, transcript levels for the *pvmsp*3.1 (*pvmsp*3-γ) and *pvmsp*3.9 genes showed 25.5- and 9.9-fold changes, respectively, and in the schizont stage, *pvmsp*3.2, *pvmsp*3.7 and *pvmsp*3.9 transcripts showed >6-fold increases relative to gDNA. The comparison of the levels of any *pvmsp*3 gene transcript between the trophozoite and schizont stages revealed two genes with noticeable transcriptional distinctions: *pvmsp*3.1 (*pvmsp*3-γ), which decreased 4.5-fold, and *pvmsp*3.2, which increased 6.9-fold. The products generated from these experiments were also separated by agarose electrophoresis to confirm the real-time PCR results (data not shown).

### Recombinant PvMSP3s Contain Antigen-specific and Cross-reactive B Cell Epitopes

To support investigations regarding the expression of the PvMSP3 family, and to specifically distinguish potential differences in protein expression between family members, recombinant proteins and specific antisera were produced representing each member of the family. The recombinant proteins produced included the unique central coiled-coil regions of PvMSP3.1 through PvMSP3.9, the coil-coiled region plus the C-terminal of PvMSP3.10 (PvMSP3-α) and the near full-length sequence of PvMSP3.11 (Fig. 1B).

The antiserum produced against each recombinant PvMSP3 showed extensive cross reactivity as determined by immunoblot (Fig. S5), preventing the use of these antisera to identify the expression of the individual proteins. The cross-reactive antibodies from each specific rPvMSP3 antiserum were eliminated by affinity purification. Individual rPvMSPs were selectively bound to Affi-Gel beads and used to adsorb antibody reactivities in a strategic manner, starting with the strongest cross-reactivities (Fig. S6). For example, cross-reactive antibodies present in the rPvMSP3.1 antiserum were removed by serial passage through affinity columns containing rPvMSP3.2, rPvMSP3.8, and rPvMSP3.9 proteins. We found that it was not necessary to run each antiserum through all ten protein-coupled resins. Antibodies specific to each PvMSP3 family member were attained after three to seven sequential passages over different selected affinity columns. These results indicate that each rabbit antiserum contained antibodies that were specific for each rPvMSP3 immunogen, as well as cross-reacting antibodies, which could be removed by strategic adsorption procedures.

Antibodies recognizing the central domain of most PvMSP3s (rPvMSP3.1 - rPvMSP3.9) did not recognize rPvMSP3.10 or rPvMSP3.11, expressed as full-length proteins. In contrast, the anti-rPvMSP3.10 and anti-rPvMSP3.11 sera recognized all of the other rPvMSP3s but did not cross-recognize each other. All anti-rPvMSP3 sera did not recognize unrelated His-tagged recombinant proteins cloned in the same expression vector or *E. coli* lysates (Fig. S5).

### PvMSP3 Family Members are Differentially Expressed in Schizonts

**Figure 5:**
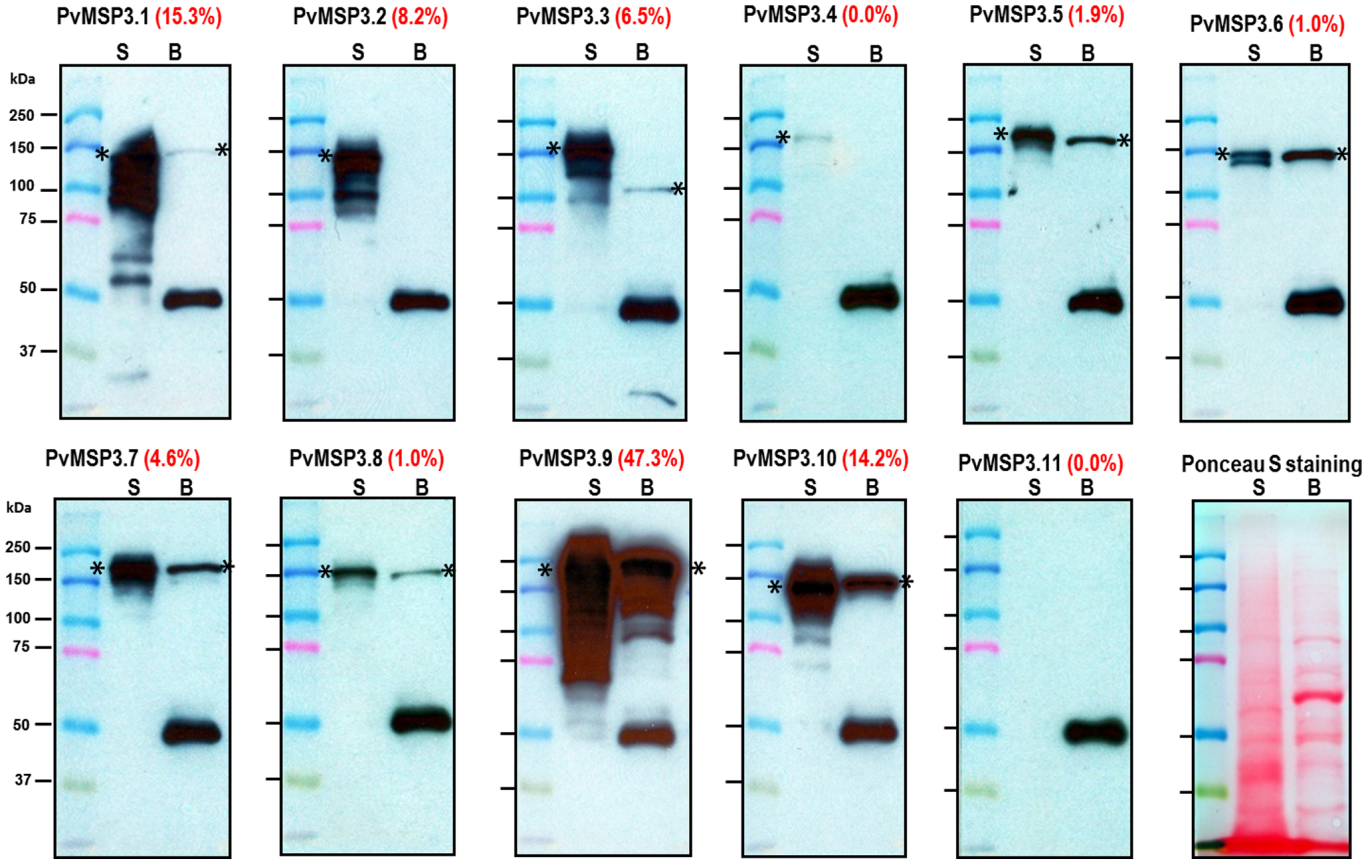
Native protein expression of the PvMSP3 family members by quantitative immunoblot. *Plasmodium vivax* parasite extracts representing late-stage trophozoites and early schizonts from the Belem strain (B) or schizont stage from the Salvador I strain (S) were separated by SDS-PAGE, transferred to nitrocellulose membranes and probed with primary antisera at 1∶10,000 dilution. The expression level of each gene in the Salvador I strain refers to the relative percentage of all PvMSP3 family members (red). Asterisks mark the position of the various MSP3 proteins of the expected size recognized by specific antisera. A 40 kDa band was consistently detected in blots using Belem strain and may be accounted as non-specific reactivity.

The expression of the PvMSP3 family was further evaluated by quantitative immunoblot assays. Affinity-purified, PvMSP3-specific antisera were used to probe immunoblots containing *P. vivax* proteins extracted from infected RBCs containing late trophozoites and schizonts of different stages of maturity from *P. vivax* (Salvador I strain) and Belem strain parasites (Fig. 5). Ten of the eleven predicted PvMSP3 proteins (PvMSP3.1 through PvMSP3.10) were detected in the Salvador I strain, but at quantitatively different levels; PvMSP3.11 was the only protein not detected in these preparations. An especially large (47.3%) amount of the total amount of PvMSP3 detected corresponded to PvMSP3.9. By comparison, <0.8% of the total PvMSP3 protein corresponded to PvMSP3.4 or PvMSP3.8. The comparative levels of the originally described PvMSP3 family members PvMSP3.1 (PvMSP3-γ), PvMSP3.3 (PvMSP3-β) and PvMSP3.10 (PvMSP3-α) were found to be approximately 15.3%, 6.5%, 14.2%, respectively. Antisera reactive with PvMSP3.1, 3.2, 3.3, 3.9 and 3.10 also recognized small fragments, presumably breakdown products. As noticed in previous studies, likely due to aberrant migration of coiled-coil proteins [13], [14], the apparent molecular weights of PvMSP3s after polyacrylamide gel separation were at least 50% greater in size than their predicted value based on deduced protein sequences (Fig. 5, Table 1).

Eight antisera showed cross reactivity with the Belem strain rPvMSP3 proteins, in support of the likelihood that there is cross-reactivity of antibody immune responses against different MSP3 family members expressed in human infections. Predicted counterpart PvMSP3.1, 3.5, 3.6, 3.7, 3.8, 3.9 and 3.10 proteins were recognized, but not 3.2, 3.4 and 3.11. PvMSP3.11 was not expressed in either strain. Of potential significance in terms of understanding the function and relative immunodominance of these proteins, PvMSP3.9 was detected in the greatest abundance in both strains.

We also tested the reactivity of the panel of antibodies against *P. cynomolgi* (Berok strain) and *P. knowlesi* (H strain) schizont extracts in immunoblots. *P. cynomolgi* extracts were detected by all rPvMSP3 antisera except anti-rPvMSP3.4 and PvMSP3.5. Fewer positive reactions were detected in *P. knowlesi* (Fig. S7). Only two MSP3 proteins are present in *P. knowlesi.* They are encoded on different chromosomes and have a high degree of homology in the regions flanking the central coiled-coil domain ([36] and unpublished data).

### Native PvMSP3 Proteins were Detected on the Merozoite Surface

**Figure 6:**
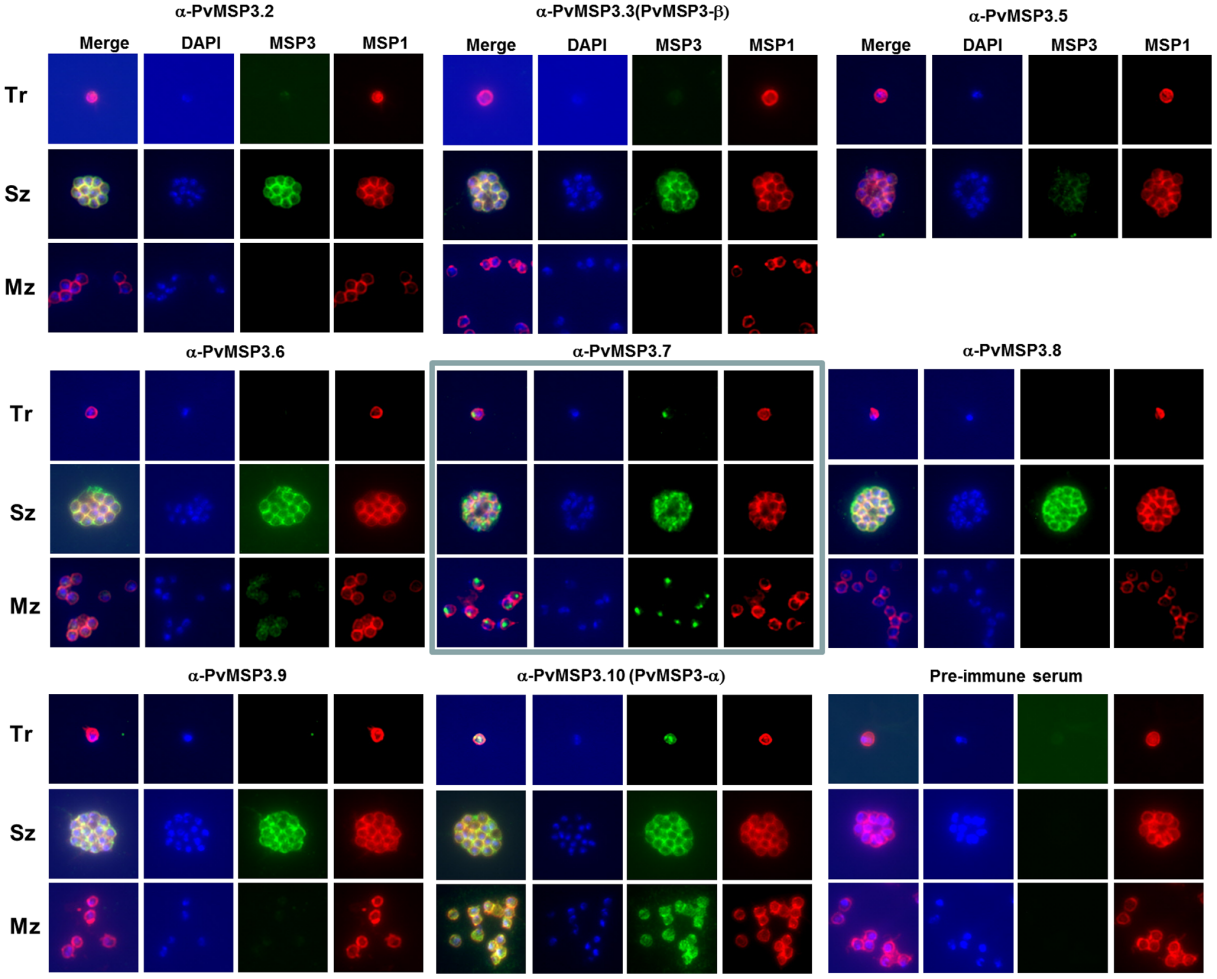
Immunofluorescence reactivity of *P. vivax* MSP3 proteins in Salvador I parasites. Air-dried thin smears were probed with each adsorbed rabbit anti-rPvMSP3 serum, followed by goat anti-rabbit IgG conjugated with Alexa 488 (green). A positive monoclonal antibody, 3F8.1A2, was included to detect PvMSP-1 followed by goat anti-mouse IgG conjugated to Alexa 555 (red). Parasite nuclei were stained with DAPI (Blue) in ProLong® Gold antifade reagent. Tr = trophozoites, Sz = schizonts, Mz = free merozoites. A merozoite surface protein fluorescence pattern was observed with each anti-rPvMSP3 on the schizont stage except for anti-rPvMSP3.7 (light blue square).

The expression of native PvMSP3 family members in the infected RBCs was then studied using the affinity purified rPvMSP3 antibody reagents. Indirect immunofluorescence assays were conducted using each of the affinity purified rPvMSP3 antibodies, DAPI nuclear stain and a well-characterized PvMSP1 monoclonal antibody [37] for co-localization studies on blood smears containing *P. vivax* (Salvador I strain) trophozoites, schizonts and free merozoites (Fig. 6). Several of the PvMSP3 fluorescent patterns appeared to fill the area surrounding the differentiated merozoites within the parasitophorous vacuole. Specifically, eight of the proteins (PvMSP3.2, 3.3, 3.5, 3.6, 3.7, 3.8, 3.9 and 3.10) were detected in mature schizonts. Three of the proteins (PvMSP3.6, PvMSP3.7 and PvMSP3.10) were also detected with free merozoites. Only two of the proteins (PvMSP3.7 and PvMSP3.10) were detected by IFA in trophozoites, schizonts and free merozoites. Three proteins, PvMSP3.1, PvMSP3.4 and PvMSP3.11 were not detected in these assays (not shown). The fluorescent patterns observed by IFA for the various PvMSP3 antisera resemble that of a merozoite surface fluorescence pattern exemplified by PvMSP1 antibodies, included in these assays for comparison (Fig. 6).

### PvMSP3.7 Uniquely Localizes to the Apical End of the Merozoite

Unexpectedly, PvMSP3.7 specific antiserum produced a distinct fluorescent pattern in both mature schizonts and free merozoites that was not typical of a MSP fluorescent pattern and was not suggestive of localization in the PV space. Rather, the observed pattern was typical for antigens localized in organelles at the apical end of merozoites, in this case as a large single dot. Moreover, immunoblot experiments distinctly detected a protein of about 200 kDa in both Salvador 1 and Belem strain parasites without any other bands detected. These data suggest that this gene, despite being denoted as *pvmsp*3.7, based on the criteria established and summarized above for MSP3s, encodes a unique protein that becomes specifically expressed at the apical pole of merozoites (Fig. 6).

## Discussion

This study presents a detailed analysis of the clustered 11-member *pvmsp*3 gene family in the prototype genome of the Salvador 1 strain of *P. vivax* [30] and we show that 10 of the 11 predicted PvMSP3 proteins are expressed. Transcripts for all 11 genes were detected in trophozoite and schizont parasite RNA preparations. While we confirmed the expression of 11 transcripts, there were distinct differences in the amount of the various *pvmsp*3 transcripts detected, which could reflect differential expression or stability of the transcripts. These results could be explained by differences in actual transcription, post-transcriptional turnover of the RNA, less than 100% purity of the stage-specific samples, or the less than perfect integrity of the RNA samples with induced bias in the RT-PCR experiments. Only *pvmsp*3.11, the smallest of the gene family members and located at the end of the cluster, while transcribed in the blood stages, was not expressed as a protein detectable by either immunoblot using parasite lysates or IFA. We attempted but were not able to amplify transcripts that could represent polycistronic messages supporting the view that each gene is driven by its own promoter, as is typically the case in *Plasmodium* [38], with few exceptions reported [39], [40].

The fact that as many as 10 of the 11 *pvmsp*3 genes have been shown here to be translated, with the expression of most members confirmed during the trophozoite and schizont stages of development, suggests that the parasite maintains the expression of multiple structurally similar but highly polymorphic proteins with putative redundant functions. However, certain members may also have the potential to perform unique functions. The latter possibility is supported by the fact that the timing of expression is not identical, and the expression patterns and locations were not the same for all family members. The timing of expression and precise localization of the PvMSP3s differ in trophozoites, schizonts or free merozoites, with the expression of the majority of the proteins confirmed, as anticipated, in the schizont stage. Together, this finding also supports the view that the role of each individual PvMSP3 may be unique, yet complementary. An alternative possible outcome that might have been observed is the restricted expression of just one or a few members of the *pvmsp*3 gene family at a given time or in certain strains as has been reported for the large *var* gene family, which encodes variant antigens that become expressed at the surface of *P. falciparum* infected red blood cells [41]. Some members of the *ebl* and *rbl* invasion ligand gene families, on the other hand, may represent redundant copies and provide alternative ligands for entry into erythrocytes [12], [42].

Consistent with global gene expression studies using a combination of microarray and proteomic technology, whereby transcripts and protein expression in *Plasmodium* do not always coincide [43], the amount of individual *pvmsp*3 transcripts as determined by quantitative RT-PCR at a particular stage did not necessarily correlate with the amount of protein detected by immunoblot or IFA. For example, in the schizont stage, where expression of MSP3s would be expected, the highest level of transcripts detected corresponded to *pvmsp*3.2, followed by *pvmsp*3.7, *pvmsp*3.9 and *pvmsp*3.11. However, the most abundant protein detected was PvMSP3.9, equivalent to almost half the amount of the total PvMSP3 produced from all members combined. The gene family could be evolving such that there is a level of acceptable relaxation in the control of expression and redundancy in function of the members of this family. PvMSP3.1 (PvMSP3-γ), on the other hand, showed a level of high protein expression by immunoblot (15.3% of total amount of PvMSP3 proteins), but was not detected at all by IFA on air-dried trophozoites and schizonts. This result suggests that the B cell epitopes in the native protein are mostly conformational and the rabbit antibody response generated against the PvMSP3.1 recombinant protein may have been mostly against linear epitopes.

Previous studies on *P. vivax* (Belem strain) using a suspension staining method showed that PvMSP3.10 (PvMSP3α) and PvMSP3.3 (PvMSP3β) localized to the surface of free merozoites [13], [14]. The fluorescence pattern on air-dried parasites diffusely covered the body of the parasite during early stages of schizogony and the typical “bunch of grapes” pattern was only revealed in fully segmented schizonts, consistent with the proteins ultimate presence at the surface of the matured merozoites. Since PvMSP3s do not have transmembrane domains, it was thought that these soluble proteins could associate with other merozoite surface proteins through protein-protein interaction by means of the coiled-coil domain [13]. In this study, most of the PvMSP3-specific antibodies reacted with segmented schizonts localizing to the merozoite surface and the parasitophorous vacuolar space (Fig. 6 and S4). The lack of reactivity on air-dried free merozoites with α-rPvMSP3.2, rPvMSP3.3, rPvMSP3.8 and rPvMSP3.9 (Fig. 6) may be due to low-affinity interactions with the free merozoite surface, a lack of antibody recognition of native protein epitopes, because the antigen is not present at the free merozoite surface or some combination of these possibilities.

Most uniquely in respect to the above spatial patterns of the majority of MSP3 family members, antibodies against the coiled-coil region of PvMSP3.7 have localized this protein at the apical pole of merozoites during late schizogony and in free merozoites, clearly differentiating this protein and its possible function from the other PvMSP3 family members. A specific protein band of ∼200 kDa was detected in Salvador I and Belem strain lysates, without any additional bands that would suggest the presence of multiple proteins with cross reactive epitopes. Proteomic experiments analyzing anti-rPvMSP3.7 immunoprecipitates would therefore be expected to corroborate this result. Proteins that localize to the apical pole of merozoites tend to be involved in the recognition and invasion of host erythrocytes and are thus also regarded as potential vaccine targets [8]–[12]. It remains to be determined whether this protein specifically localizes to the merozoite’s micronemes, rhoptries or other organelles, but it certainly warrants further study, particularly since relatively few proteins have been defined at the apical pole of *P. vivax* merozoites.

Future experiments disrupting or altering the expression of PvMSP3.7 or other members of the family will be required to study the specific function of each PvMSP3, and determine which if any, or what set of PvMSP3 proteins, are essential for the parasite’s survival or alter the parasite’s growth. It is of interest in this regard that disruption of the first-discovered *msp*3 gene in *P. falciparum*, now designated as *pfmsp*3.1 [35], as well as *pfmsp*3.3 and *pfmsp*3.7 caused no apparent change in the parasite’s growth, multiplication and viability in *in vitro* cultures [44], [45]. Only one study has been reported to date showing the successful genetic manipulation of *P. vivax*, and this has been via transient transfection of the parasite [46]. Genetic integration has been achieved with *P. cynomolgi* [47], and this species can also be used as a model for such studies.

Comparing and contrasting the MSP3 members from *P. falciparum* and other primate malaria species with the *P. vivax* proteins have identified specific characteristics, one of the most curious being the N-terminal NLRNG motif present in the sequences of all eleven PvMSP3 family members, 8–17 amino acids downstream from the putative signal peptide. The function of the NLRNG motif is not known. We speculate that it may have a role in trafficking and localization of these proteins, or be essential for their conformation. This sequence is strikingly conserved across the primate malaria species (*P. falciparum, P. knowlesi*, *P. cynomolgi* and *P. vivax*), to the extent that it has become recognized as a molecular signature for MSP3 proteins [13], [14], [35]. The identification of this motif amidst otherwise diverse amino acid sequences was, in fact, instrumental in the recognition of the six *msp*3 family members in *P. falciparum* [35], particularly in the absence of a central domain with heptad repeats in all but the first relatively small (48 kDa) member discovered in *P. falciparum* (PfMSP3.1) [16], [48].

It is intriguing that each of the *P. vivax* and simian malaria *msp*3 encoded proteins have a predominant central domain with predicted coiled-coil structure (Fig. 1B, S2 and Table 1), while this is not typically the case for the *P. falciparum* MSP3 family [35], with the exception of PfMSP3.1. PfMSP3.1 has three blocks of tandemly repeated heptads with the consensus sequence AXXAXXX and predicted to form coiled-coil structures [16], [17]. In contrast, the central region of each PvMSP3 contains as many as 24 blocks of heptad repeated motifs, and a wide range of predicted coiled-coil structures covering 66.1–84.2% of the protein (Fig. 1B and S2). The MSP3 families are otherwise quite different between *P. vivax* and *P. falciparum*. The C-terminal domain in PfMSP3.1 (and the other PfMSP3s [35]) is characterized by the presence of a leucine-zipper, which was shown to be important for the formation of dimers and tetramers [19]. The leucine-zipper motif is not present in any of the *P. vivax* MSP3s, raising questions about the higher potential structural complexity of these proteins and how they may form.

While the central coiled-coil structure may function as previously proposed to enable the positioning of this protein at the surface of merozoites, particularly in the absence of a C-terminal hydrophobic transmembrane region or GPI anchor, since most PfMSP3 family members do not have central coiled-coils regions, surface localization may not be the primary role of this family of proteins [35] and the same may be true for *P. vivax*. It remains conceivable that these proteins may have a critical primary role in the parasitophorous vacuole, as they sometimes appear to be abundantly expressed in the space surrounding the developing schizonts. Like PfMSP3 [29], [48], they may also function to elicit antibody-dependent cellular inhibition (ADCI) activity as a means to control the rise in parasitemia. This mechanism has not yet been demonstrated in *P. vivax,* though it largely forms the basis of developing effective PfMSP3 vaccines [24], [25], [49], [50].

If PvMSP3 vaccines are to be pursued, a more thorough understanding of the potential for diversity [21], [22] and immunogenicity of all members of this family will be important. In malaria endemic areas, PfMSP3 alleles have been shown to induce allele-specific immunity under natural immune selection [51], [52]. We detected extensive cross reactivity in the antibody responses generated against all 11 recombinant PvMSP3s, suggesting that the naturally acquired immune response generated to each protein may provide broad protection against the potential repertoire of expressed proteins; however, this needs to be analyzed in the field, complementing the few studies published to date showing the presence of naturally acquired immunity to PvMSP3-α [26]–[28]. Cross-reacting antibodies could easily be removed by running the various anti-rMSP3 sera through columns containing selected recombinant proteins, but not the complete panel, suggesting that these proteins have specific as well as shared B cell epitopes.

In summary, we have characterized the expression profiles of 11 *pvmsp*3 gene family members, which are positioned in tandem in a head-to tail fashion in chromosome 10 in the *P. vivax* (Salvador I strain) genome. We show that all 11 *pvmsp*3 genes from the *P. vivax* Salvador I strain (propagated in *S. boliviensis* monkeys) were transcribed in blood-stage parasites, and ten were expressed as protein, with eight PvMSP3s confirmed in mature schizonts/merozoites, and one localizing to the merozoite’s apical pole. The function of each protein is unknown, and naturally acquired immune responses have so far only been studied against PvMSP3.10 (PvMSP3-α) [26]–[28], leaving much to be understood about the biological and immunogenic role this family of proteins has in terms of immunomodulation and protection. Finally, just as *pvmsp*3*-α* and *pvmsp*3*-β* have become genetic markers of diversity and tools for studying population genetics of *P. vivax* [213–23,53–60], other members of the family may be similarly suited.

## Materials and Methods

### Ethics Statement

All animal experiments in the current study were conducted in AAALAC-accredited facilities at the Yerkes National Primate Research Center in accordance with the Animal Welfare Act and the Guide for the Care and Use of Laboratory Animals. All experimental protocols were approved by Emory University’s Animal Care and Use Committee. Adult Bolivian squirrel monkeys (*Saimiri boliviensis*) were housed indoors under conditions of controlled temperatures (67 to 77F) in same-sex pairs. Each cage was equipped with perches and at least the minimum amount of floor space required by federal rules, regulations, and guidelines. Animals were fed a diet of LabDiet New World Monkey Diet (Richmond, IN) twice daily. Peanuts and at least 1/4 orange were offered as enviromental enrichment. All animals are monitored daily by veterinary staff for potential health problems. All animals were trained by experienced personnel to voluntary present at the front of the cage for skin prick using positive reinforcement. For large blood collection volumes, animals were sedated with Ketamine (dose/route) or Telazol (dose/route) to minimize distress. No animals were sacrificed in this study.

### Parasite Collection, Genomic DNA and RNA

*Plasmodium vivax* (Salvador I and Belem strains) [61], [62] parasites were obtained from blood-stage infections in *Saimiri boliviensis* monkeys. The *P. vivax* late-stage trophozoite-infected erythrocytes or early schizonts were purified as described previously [61] and aliquoted samples were stored as frozen stocks. Genomic DNA (gDNA) was extracted as described previously [63]. Total RNA was extracted from the Salvador I strain samples using a RiboPure™-Blood kit (Ambion), followed by two rounds of DNase I digestion to remove any remaining gDNA. Since the *pvmsp*3 genes have a one-exon gene structure, trace contamination of gDNA in RNA samples would dramatically affect the results of quantitative real-time Reverse Transcription - Polymerase Chain Reactions (qRT-PCRs). DNA contamination was evaluated by PCR amplification of total RNA samples without reverse transcription using Taq DNA polymerase (Invitrogen), either a weak or no amplicon was detected, thus, we concluded that our RNA samples contained little or no gDNA.

### Polymerase Chain Reaction and Reverse Transcription-PCR

First strand complementary DNA (cDNA) was synthesized using the SuperScript™ III First-Strand Synthesis System (Invitrogen) with an oligo(dT)_20_ primer at 50°C, and resulting cDNA samples were stored at −20°C and thawed as needed for RT-PCR studies. PCR and RT-PCR were performed using gDNA or single strand cDNA as templates, respectively, the SYBR Green PCR Master Mix system (Applied Biosystems), the iCycler machine (Bio-Rad) and gene-specific primers (Table S1). Amplification conditions consisted of one cycle of 15 min at 95°C followed by 45 cycles of 95°C for 30 sec, 58°C for 15 sec, and 72°C for 60 sec. At the end of the reactions, using SYBR Green dye, the melting curve of each sample was acquired and analyzed. After amplification, the size of the PCR products amplified from both gDNA and cDNA were analyzed by electrophoresis on 1.5% agarose gels.

### Real-time Quantitative RT-PCR

Real-time qRT-PCR was performed using TaqMan® Universal PCR Master Mix Kit (Applied Biosystems) and the iCycler (Bio-Rad) detection system. An initial incubation at 55°C for 2 min activated the AmpErase UNG (degradation of potential carry-over DNA contamination). Then, amplification conditions consisted of one cycle of denaturation (95°C, 10 min) and 45 cycles of amplification (95°C, 15sec, and 60°C, 1 min). 25 ul reactions contained 1 ng of gDNA or cDNA, 0.3 µM each primer, 0.2 µM Taqman probe and 12.5 µl of TaqMan® Universal PCR Master Mix. Real-time qRT-PCR experiments using pairs of primers (Table S1) representing each of the 11 *pvmsp*3 genes were repeated in duplicate including the *P. vivax* seryl-tRNA synthetase gene as an internal control and both stage-specific cDNA and gDNA as templates. Negative controls without primers were also included in the qRT-PCR amplifications.

### qRT-PCR Data Analysis using the 2^−ΔΔCt^ Method

The relative quantification method was used to measure gene transcription levels. First, the amplification efficiency of primers pairs representing the 11 *pvmsp*3 genes were validated using dilutions of gDNA [64]. The *P. vivax seryl*-tRNA synthetase gene served as an internal control. The validation experiment, using gDNA diluted in a 100-fold range, demonstrated that the efficiencies of all primer sets amplifying the *pvmsp*3 gene family members, and the internal control reference gene, were approximately equal (data not shown). *P. vivax* Salvador I gDNA, known to harbor single copies of each *msp*3 gene family, was used as a calibrator in the qPCR, and data was analyzed by the 2^−ΔΔCT^ method [64]. To calculate the relative quantification of each *pvmsp*3 gene, the ΔΔCt formula was used as follows ΔΔCt = (Ct, *pvmsp*3 − Ct, *pvseryl-*tRNA synthetase)χ – (Ct, *pvmsp*3 - Ct, *pvseryl-*tRNA synthetase)y, where χ = cDNA and y = gDNA.

### Production of Eleven PvMSP3 Recombinant Proteins in *Escherichia coli*

The alanine-rich coiled-coil central domains of all but one PvMSP3 were expressed as recombinant fusion protein (rPvMSP3) in *E. coli* using the Champion™ pET200 Directional TOPO® expression vector **(**Invitrogen**)**, the PvMSP3.10 (PvMSP3α) gene was alternatively expressed in the vector pET-24d(+) expression vector **(**Novagen**)**. All fragments were amplified from *P. vivax* (Salvador I) gDNA using high fidelity KOD DNA polymerase (Novagen) and directional ligation. Positive clones were confirmed by DNA sequencing and subsequently transformed into BL21 Star™ (DE3) *E. coli* cell (Invitrogen) for expression. Fusion proteins were induced by the addition of 1 mM isopropyl-b-D-thiogalactopyranoside (IPTG) at 37°C for 3 hr. Recombinant proteins (rMSP3s) were expressed as 6X-His tagged fusion proteins and were purified using a combination of HisTrap™ HP columns (Amersham) and gel filtration or anion exchange columns. New Zealand White female rabbits were immunized with the PvMSP3 recombinant proteins for production of a set of specific polyclonal antisera following a three-dose immunization schedule (Covance).

Reactivity of each rabbit polyclonal serum was tested against its own recombinant and the other 10 recombinant proteins by immunoblot. All rabbit antisera showed variable but selective cross-reactivity against some rPvMSP3. Thus, the antisera were adsorbed with highly reactive PvMSP3 recombinants coupled to Affi-Gel 15 (Bio-Rad). Briefly, 1.5 mg of purified recombinant protein was coupled to 2 ml Affi-Gel 15 using the recommended protocol. After blocking the active protein binding ester sites with 1 M ethanolamine HCI (pH 8) and equilibration of the resin with 1xTBS containing 10% dry milk, 200 µl of each antiserum was run through the Affi-Gel 15 column. The flow-through was then run through columns containing other selected antigens that were also causing cross-reactivity. All antisera showed no cross-reactivity by immunoblot after performance of these adsorption procedures (Fig. S6). The affinity purified antisera were stored at −20°C in 10% dry skim milk.

### Indirect Immunofluorescence Assays

Indirect immunofluorescence assays (IFA) were conducted on air-dried thin smears of *P. vivax* (Salvador I strain) iRBCs purified from blood-stage infections of *S. boliviensis,* using Institutional Animal Care and Use Committee approved protocols. When necessary, the desired stage of parasite development was obtained by maturation in short-term *in vitro* culture [61], and slide collections were stored at −80°C as frozen stocks. Upon removed from −80°C storage, the slides were immediately fixed with 0.25% paraformaldehyde in 1x DPBS at room temperature for 20 minutes. Primary antibodies, adsorbed rabbit anti-PvMSP3 sera and PvMSP1 monoclonal antibody (3F8.1A2), were mixed and diluted at 1∶250 and 1∶1000 respectively with 1x DPBS containing 0.2% fraction V bovine serum albumin (BSA), and added to each slide and incubated at room temperature for 1.5 hr, followed by three washes with 1x DPBS containing 0.2% BSA. Goat anti-rabbit IgG (highly cross-absorbed by human and mouse IgG) conjugated with Alexa 488 dye (Invitrogen) and goat anti-mouse IgG (highly cross-absorbed by bovine, goat, rabbit, rat, and human IgG and human serum) conjugated with Alexa 555 dye (Invitrogen) were diluted at 1∶100 and used as secondary antibodies. Finally, the slides were washed four times with 1x DPBS containing 0.2% BSA, mounted with ProLong® Gold antifade reagent with DAPI (Invitrogen) and covered with a cover slip. Antibody binding and DNA staining were assessed using a Zeiss Z.1 fluorescence microscope.

### Quantitative Immunoblots

Whole cell lysates of *P. vivax* Belem (trophozoite and early schizonts) or Salvador I strains (schizonts) iRBCs were solubilized with reducing (2-β-Mercaptoethanol) sample buffer. Ten µl of sample were separated by SDS-PAGE and transferred to nitrocellulose membranes using a Bio-Rad Mini Trans-Blot Cell (Bio-Rad). The membranes were probed with adsorbed polyclonal rabbit antisera (1∶10,000) against each rPvMSP3 followed by goat anti-rabbit serum 1∶5000) conjugated to horseradish peroxidase (Promega, Madison, WI, USA). The band intensities were quantified using the Quantity One software (Bio-Rad) with the adjusted volume method and expressed as a percentage of total MSP3 protein. Simultaneously, duplicate samples were stained with mixture antibodies of all eleven rabbit anti-PvMSP3 or Ponceau S reagent after SDS-PAGE separation and nitrocellulose membrane transfer for normalization of the protein concentration in the parasite lysates.

### DNA and Protein Sequence Analysis

The Multicoil software (http://groups.csail.mit.edu/cb/multicoil/cgi-bin/multicoil.cgi ) was used to compare the input amino acid sequence to a database of known parallel two-stranded coiled-coils. A cutoff of 0.5 was applied for scoring the coiled-coil structures present in the PvMSP3 predicted proteins. The putative signal peptide cleavage site was predicted using SignalP 3.0 (http://www.cbs.dtu.dk/services/SignalP/) which uses the neural network model [65]. The TMHMM Server v. 2.0 (http://www.cbs.dtu.dk/services/TMHMM-2.0/) was used to search for transmembrane domains. The GPI Modification Site Prediction algorithm by Eisenhaber’s method [66] was used to evaluate the presence of post-translational modifications (http://mendel.imp.ac.at/gpi/gpi_server.html ). Conserved motifs were evaluated using the MEME package (http://meme.sdsc.edu/meme4/cgi-bin/meme.cgi).

## Supporting Information

Figure S1 **Coiled-coil region of the PvMSP3 protein family members predicted by a program of Multicoil (http://groups.csail.mit.edu/cb/multicoil/cgi-bin/multicoil.cgi) with 0.5 cut-offs for scoring a coiled-coil structure.** Below the amino sequence are the predicted frames of the coiled-coils represented by the ‘abcdefg’ convention for the heptad repeats. The repeated AXXAXXX sequence pattern representing predicted coiled-coil heptads is marked by a red underline.
(PDF)

Figure S2 **The typical tandemly-repeated heptad motif AXXAXXX, predicted to form α-helical coiled-coil structures, was found in the putative MSP3 proteins analyzed (see sequence IDs in Table S2) using the MEME algorithm (http://meme.sdsc.edu/meme4/cgi-bin/meme.cgi).** Numbers in parentheses refers to amino acid position.
(PDF)

Figure S3 **Glutamate-rich motifs from the C-termini of MSP3 proteins.** The high content of glutamic acid (E) residues are present amidst other acidic amino acids in clusters, resulting in hydrophilic C-terminal regions.
(PDF)

Figure S4 **Immunofluorescence experiments showing the expression of the PvMSP3 proteins in relation to PvMSP1.** Mixed stages of *P. vivax* (Sal 1) infected RBCs in air-dried thin smears were stained with a mixture of antibodies recognizing all PvMSP3 proteins (Green, Alexa 488). A monoclonal antibody (3F8.1A2) was used to detect PvMSP1 (Red, Alexa 555). Parasite nuclei were stained with DAPI (Blue) in ProLong® Gold antifade reagent. The slides were fixed with 0.25% paraformaldehyde in 1x DPBS at room temperature for 20 min, immediately upon removal from −80°C. **4A**. Individual IFAs and the merged co-localization images are shown. **4B**. different layers of one schizont were observed using a Zeiss LSM 510 META confocal microscope. The upper panel shows the merged pictures with the green (PvMSP3), red (PvMSP1), blue (parasite nuclei) and brightfield channels, and the lower panel shows the merged pictures without the brightfield channel.
(PDF)

Figure S5 **Cross reactivity is not caused by recombinant protein expression vector residues or 6xHis tag.**
(PDF)

Figure S6 **Cross-reactive antibodies present in the rPvMSP3 antisera were removed by serial passage through affinity columns.**
(PDF)

Figure S7 **Detection of PvMSP3 homologs PcyMSP3 and PkMSP3 with rabbit antiserum against specific rPvMSP3s.** Same volume (10 ul) of *P. cynomolgi* and *P. knowlesi* parasite extracts representing schizont stage from the Berok and H strain respectively were separated by 7.5% SDS-PAGE, transferred to nitrocellulose membranes and probed with primary antisera at 1∶5,000 dilution. All membranes were exposed for the same length of time for chemiluminesence detection.
(PDF)

Table S1 **Primers used for gene-specific PCR, quantitative real-time RT-PCR, and recombinant protein expression.**
(PDF)

Table S2 **Gene accession numbers or identification numbers used for analysis.**
(PDF)

Table S3 **Raw data of qRT-PCR detection of transcript level of PvMSP3 family members with TaqMan probes.**
(PDF)
