# Supplementary material for: Plasmodium vivax Merozoite Surface Protein-3 (PvMSP3): Expression of an 11 Member Multigene Family in Blood-Stage Parasites
Source: PLoS One. 2013 May 23;8(5):e63888. doi: 10.1371/journal.pone.0063888 (PMC3662707; doi:10.1371/journal.pone.0063888)
Supplement: Figure S1 — Coiled-coil region of the PvMSP3 protein family members predicted by a program of Multicoil (http://groups.csail.mit.edu/cb/multicoil/cgi-bin/multicoil.cgi) with 0.5 cut-offs for scoring a coiled-coil structure. Below the amino sequence are the predicted frames of the coiled-coils represented by the ‘abcdefg’ convention for the heptad repeats. The repeated AXXAXXX sequence pattern representing predicted coiled-coil heptads is marked by a red underline. (PDF) [file pone.0063888.s001.pdf]

Figure S1. Coiled-coil region of the PvMSP3 protein family members predicted by a program of Multicoil (http://groups.csail.mit.edu/cb/multicoil/cgi-bin/multicoil.cgi) with 0.5 cut-offs for scoring a coiled-coil structure. Below the amino sequence are the predicted frames of the coiled-coils represented by the ‘abcdefg’ convention for the heptad repeats. The repeated AXXXAXXX sequence pattern representing predicted coiled-coil heptads is marked by a red underline.

PvMSP3.1

```
   1    MKHLSGIPLFVLFLNLYVLQNNVVSNENVNLKNPNLRNGWAGKNVTLQDEQSGLVAEDGEEVNTKEDQNE
  71    LLHKSEETDFQTQGQEVKEIGGSENEKKQKQIKKANEAKEKALKEQKEAEDEVKKIEEALDKVKKAKAEA
                                abcdefgabcdefgabcdefgabcdefgabcdefgabcdefgabcd
 141    EEEAEIKKVVEKAKGVADKAKETAMNKAKKSKDEAEKIAGVNTNSMAYLYAGNAVTAEIEAKAEKEKAKK
        efg                                                      abcdefgabcdef
 211    AAEIAKHAVDAYELKKEAEKAQEEAEAAKTEIEKLSKVYKEGNVTEAVQSTAEAEKSASGASAKVKEAAH
        gabcdefg         abcdefgabcdefgabcdefg            abcdefgabcdefgabcdef
 281    NVAKKLMDAVQKLEKVSTELPKDNEQAATIDNVNEVVTEAVKEKEKAMISAEVAKAEAANAEAQLAKIEA
        g                                            abcdefgabcdefgabcdefgabcd
 351    ERAKYEANKIAEEYTDNVKGEAKKAEEKANEASSKATEASNNAKGASGEEKQTNPQAANVKAKAGEAIKA
        efgabcdefg           abcdefgabcdefgabcdefgabcdefg
 421    AKEAKKAKTEAYIALCVTKTLVAKENAKKAEQEAKNAKDKATKAAKEAEEAKKQAEKAEKITETVKNEAK
                           abcdefgabcdefgabcdefgabcdefgabcdefgabcdefgabcdefgab
 491    TATDEEAKASTGKKDAEINAGYVDEEVYAVNIEFEIAKEAAKTAAQHKALEILDKAEKNAEIAAENATAK
        cdefg                                            abcdefgabcdefgabcdefg
 561    AQEATKKAETAKTKATEAETAAKKAQDASEKAKAIAADVLAQKASTEAQSLKQEAEKLAENIKKSNVTDE
        abcdefgabcdefgabcdefgabcdefgabcdefgabcdefgabcdefg     abcdefg   abcdef
 631    EKAKADEAAKAAKDAADQASASAKKANDAKIAATNAQVVVTLQTKKAESAKAEDAAKEAMKARDKAAFEL
        gabcdefgabcdefgabcdefgabcdefgabcdefg            abcdefgabcdefgabcdefga
 701    LKIKKQDVLEQVDVSPSGSDNLNDVDEQVALEVGEQQNETEDAEPQEAEEGDEEDEEDTEEEEIQDESDH
        bcdefg
 771    TEESSAKQAAQQEKQQGEKVLNDEEAHNLLAQQHNKDNNAATEDAKLFQTIIKDFDDDDDFKNLQKDVNA
 841    LFKIK
```

PvMSP3.2

```
   1    MRQFFGTTILVLFLNLYVLQTNVVSNENVNLKNPNLRNGWAGKNLALQDEQSGIDAEDGGEENTCEGQSE
  71    LLQKSEAQVQTQGQEIKATGESDDPKKKKSPIEKANEAKDNALKEQEGAEAEVKKIEEAEKKVEGAEEGI
                                       abcdefgabcdefgabcdefgabcdefg          a
 141    KNAVKKAKEAAEFAKNTATEKAKKAKEKAGEITTASASMASFHAIVAEMAKDVAKAANENAKKAARIATH
        bcdefgabcdefg        abcdefg                             abcdefgabdefg
 211    VAKAYEAKKEAEEAKTKAEEAKKEAEKAQTEVEAAKKEAEKVMAEAEKAMAEANDAAQKLVEKPEGKKTE
           abcdefgabcdefgabcdefgabcdefgabcdefgabcdefg    abcdefg
 281    EMEWLEEVKKKVDEAIVEIQKSLDTATQNATAAGSNATKAATEANNAIPNEAKTDKEMKDNAIIKEANVK
         abcdefgabcdefgabcdefg                                       abcdefgab
 351    DVEDTANEAIKAAEQAKKSKAKAEILAGKAKIQVALEIAKIAAKVTGQAKNGIDELAKMATSANTLKKTS
        cdefgabcdefgabcdefgabcdefgabcdefg                   abcdefgabcdefgabcd
 421    TEKATEASKKAKEIEALLKKIEDIAKEEDNLVGKEEEAKKKVQEATEAKQVAIKAKAEAEIAVLVARVHL
        efgabcdefgabcdefgabcdefg          abcdefgabcdefgabcdefgabcdefg
 491    AKQEVTRLVQLADEEKNKAKKAAETAENAKNAAEKVSDKVSKNVKNASEILAAVESAKTEAQTAKTEGKN
         abcdefgabcdefgabcdefgabcdefgabcdefgabcdefgabcdefgabcdefgabcdefg
 561    AEMEAADAVEEALMLEYLLSKATDAANKAKEANGDNITSTLETAQQDALKTLGQADHIAMETFNLTQKAI
            abcdefg                                                         ab
 631    EASKKAQKAAEVAKAEAAKAEAYKAIEEVKKEEQKIKQLVAKAKESKATEEVKAAEAAEKVAKAEVDKAT
        cdefgabcdefg  abcdefgabcdefg                       abcdefgabcdefgabcde
 701    SEAEKAKTAAGEAKNASTLEAAKAKAMNAINALKEARTSIYKTEYEYLKLIKQDVLEPLKVSTEASDNVF
        fgabcdefgabcdefg
 771    DKNEQVAQEVEDRGSEENDAAEEDAAPSDVEEDEGELEDEEEEEEEVEQLEQPQEQQTDASSKDKGTAET
 841    QKSEDVLDDEGAHKLLAGELNTANKSNNAENALFKTVFDEVRDDTIQNLKQDVGQFFKGKEK
```

PvMSP3.3

```
   1    MKQLCGLAFLALLLNFLTCDNVATRGEIVNLKNPNLRNGWSMKNLSAQNEENIVHSDGSDDVTDKEEDGE
  71    VLEGQKGSPKKSAEQKVHAQEEVNKESLKSKAQNAKAEAEKAAKAAESAKENTLDALEKVNVPTELNNEK
                                    abcdefgabcdefgabcdefgabcdefg
 141    NFAESAATEAKKQEKISTEAAEEVKEIEVDGQLEKLKNEEEKTAKKARKQEIKTEIAEQAAKAQAAKTEA
                                                                   abcdefgabcd
 211    ETAQKDATTAKDEAIKETGKPKSQNTTKAVTMATEEEKKTKDEAQTASEKAGKTAEEAQKEVGKETADDD
        efgabcdefgabcdefg                abcdefgabcdefgabcdefgabcdefg    abcde
 281    KEVSQLEEEIKELERILKIVKDLASEASSASDNAKKAKLKTQIAAEVVKAEKARIEAEEAEKEAGEAKTK
        fgabcdefgabcdefgabcdefgabcdefgabcdefg     abcdefgabcdefgabcdefgabcdefg
 351    TEATEKEVLKISDESKAAKVKKAVEKAKEAEKQAKSEAEKAKGMADDAGGKGTTNLEDVLTKLSEVLTSV
        abcdefgabcdefg         abcdefgabcdefgabcdefg
 421    KSLASNAEVASKNAKKEMTKAQIAAEVAKAEKAKIEAENAKLLADTASKAAENIAKSSKAAKIANNVSTI
             abcdefg     abcdefg  abcdefgabcdefgabcdefgabcdefg
 491    AAEKSKVATEAADEAAKALDETENPESKIAEVTEKATKAVNAAEEAKKEKAKAEVAVEVAHAEVAKEKAQ
                                    abcdefgabcdefgabcdefgabcdefg         abcde
 561    EAKEAAKQVADKSKLEKAIQAADKASEKANEASKLAEEALSNLESLEKETGEIVEKVNAIEQKVQTAKNA
        fg            abcdefgabcdefgabcdefgabcdefgabcdefgabcdefgabcdefgabcdefg
 631    AIEAHKEKTKAEIAVEVAKAEEAKKEADNAKVAAEKAKETAEKIAKTSKSTEKITEEVRKATEFAKTAGD
                 abcdefgabcdefgabcdefgabcdefgabcdefg      abcdefg
 701    ETTLAATKAESEIPSEEKNQKELLDSIKQKAESAFQASQEAIKAKTEAENFLEIAKEVPKAEAAKEEAQK
                            abcdefgabcdefgabcdefgabcdefg                abcdef
 771    AATAAEEAKTEVLKIAEEVNKSDASESEKKKIETAANETAGEAEKAATFAKEAADAAKDTNKAVTLAVAK
        gabcdefgabcdefgabcdefg                 abcdefgabcdefg         abcdefga
 841    EKVEKALKAAKEAKKANEKASYALIRTKKQYALEPLEITSEAGYNITEKEEQVKEEIEEQDDKASEEEEE
        bcdefgabcdefgabcdefgabcdefg
 911    DTQQIDQTQIDEVDISVDNEEEEEGAAEEQIEGEKDTPTKEAKEEQTSGEKILDDKEAHKTLAEKFKDSN
 981    TAKTGGVEFLETLISDVGEDTLKNLQQDLHQYFKGK
```

PvMSP3.4

```
   1    MKHFGGIPLLVLFVNLYIHQNVVSNEIVNLKNPNLRNGWAGKDVTLQDEQNGLNAEDGDETDPNGSHNEL
  71    LQKSGKAELQMQAQDSDSKEDIKTKIDELVNKAEAANTKADTAVTEAEKLLKTATEAEKQIKVDEEKETT
                          abcdefg       abcdefgabcdefgabcdefgabcdefgabcdefg
 141    AATKAKEQAEKAFAEAKIKKEEAKTKKEDAKAKAKADDAKKKSEETDEAELKAKLEEVEKLVGQAEQAAS
             abcdefgabcdefgabcdefgabcdefgabcdefgabcdefgabcdefgabcdefgabcdefgab
 211    DAENEQKKAEWAAKIAEELKKAYEAKKKARESKVAAENAKNDATSAASGSKVQKVTEAIKIADDEMQTAA
        cdefgabcdefgabcdefgabcdefgabcdefgabcdefg
 281    KNATEAEGAERKAEQAASEAINAIEQKVDAQSKVNVVQNEADAASKAATAAKNAQTKAEIAVFVVKTEVS
             abcdefgabcdefgabcdefgabcdefgabcdefgabcdefgabcdefg
 351    KEEAVKSANAAEEAKTKAEEVATKSEEIKITNVTKSEKVKSAKSEAVNQAAAADKAKKEAEAAANSADKA
        abcdefgabcdefgabcdefgabcdefg       abcdefgabcdefgabcdefgabcdefgabcdefg
 421    QTSTKGIDNETTLSTAKKEAETAASNAGKAQTDASTALAKAKKAQIETEIAAEVVKAEKAKKDASSDADA
        abcdefg     abcdefgabcdefgabcdefgabcdefgabcdefg      abcdefgabcdefgabc
 491    AVEARKKAESEAEKAKSNEVAQEAAQKAKEQEAEALKAKGNAQTASDQAEKEAEGDGTEDKLAKVKEKEK
        defgabcdefg         abcdefgabcdefg    abcdefgabcdefg         abcdefgab
 561    DASNAATAAKNARTTAEIAAEVAKAQAAKTEAEAAKVAANAAKDEAQKVADESKSAEAQKAAEKASEAAT
        cdefgabcdefg         abcdefgabcdefgabcdefgabcdefg            abcdefgab
 631    TAGEKAAEAAKVADDATSVSNTATLSTMQEKANKAVEHAKEAKKAQTKAELAAEEAKAEAAKKEAQKAQE
        cdefgabcdefg                               abcdefgabcdefgabcdefgabcdef
 701    AADAAKKEAEEIAKKEKVDAKAVAEATSALDTAKTKAKEAKDKAGDVSKEVEKAEKEVEKVVGDDAQKSE
        gabcdefg              abcdefgabcdefgabcdefgabcdefgabcdefgabcdefg
 771    ITTAVSGIKGKTSEAVQAAKDAKKAKDLAEIALNVVKAEVANDKAKKAVPAAGVAKEDAKKAAASAEQPK
 841    KEAESAEEGAEIAKENEIEAGQKAKEAEDNAAVVQAKLLIAEQELKNAKAENDTEKFQSAKANALAAAEE
          abcdefgabcdefgabcdefgabcdefgabcdefgabcdefgabcdefgabcdefg  abcdefgabc
 911    AVKKGQAAEAAANAATDKAKKAAEAAEKAKKAAAESALKKKLKVLEIVKKYSKRGYNVVDNDEQVLNEVD
        defgabcdefgabcdefgabcdefgabcdefg
 981    EQASEAKEEEDEDEADDSASSDVEIEDDEEEEEEEDEEVKEEEGQAQSSAHQNSVAELENQKKESQEKSD
                                                abcdefgabcdefgabcdefgabcdefg
1051    EPPSDNNAQALLSDNYKNFTDFKKKAEDLTKNIINTIDGDAGVIDTLKDFANDVNQFILNM
```

PvMSP3.5

```
   1    MKQFCGIPLLVLFVNLYVLQNNVVSNEIVNLKNPNLRNGWVGKNLTLQDEKIVLNAEDGDEIDPNGSHIE
  71    LPQKSEKAELQMQEQGNDDNEDLKRQIGEFVDQAQLANTKADAAVAEAEKSLEKAKDAEEQVKQDEGNVT
                               abcdefg   abcdefgabcdefgabcdefgabcdefgabcdefg
 141    TATTKAKAEAQKAVDAAKTKKKDAKTNADNAKQESEKKGDQELRATLDKVKKFAEKAEQALSGAQTEQKK
              abcdefgabcdefgabcdefgabcdefg            abcdefgabcdefgabcdefgabc
 211    AEWAAKIAEELKKAYEAKRNANDSKEAAEKTKKDATSAVSDSTEQKVTASIQIIEEQKQTAVTKATEAEK
        Defgabcdefg      abcdefgabcdefgabcdefg            abcdefgabcdefgabcdef
 281    AAKETEKAAKETEQAAIEAINVIDQTTVQDKFKVVEGKADTASKAAAAAKKAQTKAQIAAYVVQTEVSKE
        gabcdefgabcdefgabcdefg                                              ab
 351    KAVKSAREANEAKTKAEDVAKESEKIKITDEDKSGNVKAAKTEAVEQANLADNAKKEAEAAAISANEAKT
        cdefgabcdefgabcdefgabcdefg       abcdefgabcdefgabcdefgabcdefgabcdefg
 421    SITVKDDDTTLNEAKTKAYDASIKAKQAEKDASNALKEAQKAQIETEIAAEVVKAEKAKKEASSDAAVAR
                  abcdefgabcdefgabcdefgabcdefgabcdefg      abcdefgabcdefgabcde
 491    EAKEKAESEANKAKSNAVAQEAAKRAQEQDVAASTAKGNAETAAKEAETAAEDNVSEETLTTVKQKVENA
        fgabcdefgabcdefgabcdefgabcdefg                             abcdefgabcd
 561    SNAATAANKARTKAEIAAEEAKAQAAKTEAEASKVAANAAKDEAQKAAEESKSAEAKKAADKASAAAKTA
        efgabcdefgabcdefg         abcdefgabcdefgabcdefg     abcdefgabcdefgabcd
 631    DEKAASAKKEADDATTESKSTTATVETMQKKADKAVEHAKEAKKAQTKAELAAEEAKAEAAKEEAKKAQE
        efgabcdefgabcdefgabcdefgabcdefgabcdefg     abcdefgabcdefgabcdefgabcdef
 701    AADAAKKEAEEIAKKEKVDAKAVAEATSALDTAKTKAKEAKDKAGDVSKEVEKAEKEVEKVVGDDAQKSE
        gabcdefg    abcdefg   abcdefgabcdefgabcdefgabcdefgabcdefgabcdefg
 771    ITTAVSGIKGKTSEAVQAAKDAKKAKDLAEIALNVVKAEVANDKAKKAVTAAGVAKEDAKKAAASAEQPK
                                                 abcdefgabcdefgabcdefg
 841    KEEESAEEDAEIAKENEIEAEQKAKEAEDNANAAQAQLKIAEQELKKAQEADNEEKFQSAKTNALAAAEE
          abcdefgabcdefgabcdefgabcdefgabcdefgabcdefgabcdefg         abcdefg
 911    AIKKGQAAEDAANEAKNNAVKAAEAAEKAKKAAAESALKKKLNVLEIVKKYSREGYNTVDSDEHVLNEVE
                   abcdefgabcdefgabcdefg
 981    EQASEEKEEGEREEEEEADHPILNDVEIDDDDEEEEEEEEEKEEEETEVKEEEDAKEEGHAQSSAHQSSI
                                   abcdefgabcdefgabcdefgabcdefgabcdefgabcdefg
1051    TELENPKKEYQEKSDKPPSDSNAQALLEDNYKNFTDFKKKAEDLTKNIINTIDGDAGVIETLKDFAKDMN
1121    QFILNM
```

PvMSP3.6

```
   1    MRQFFGTTILVLFLNLYVLQTNVVSNENVNLKNPNLRNGWAGKDVTLQDEQSGPNAKDGDEVNTNQDQIK
  71    LQQKSDETLLQMQGQDKEKVAESDDLKEAKEAKTKAEKEEAEAETVKQKILKAEKETEKAEQKIKDAINK
            abcdefgabcdefg          abcdefgabcdefgabcdefgabcdefgabcdefgabcdefg
 141    VKEYALQKESEVKEQTKAAKFAADEATAENAKEKKETANTALGEAQKAKKEMETAAVIVAEVVKAMEAEK
        abcdefgabcdefg             abcdefgabcdefgabcdefgabcdefg         abcdef
 211    ESQKANEEAQKANQEAENAKNNIINIETEKAGADNPNSKAEQERTIVNEKAKGAIENAKQASTRATGAAQ
        gabcdefgabcdefgabcdefgabcdefg                             abcdefgabcde
 281    KATGAAQKAAEEAQKAIAAITGNVKEIPPANVEEIKKIASDAVKDAKDAKKAKGEAQINAEIVKLELAKV
        fgabcdefgabcdefgabcdefg        abcdefgabcdefgabcdefg         abcdefgab
 351    EAKKVVASANKAKKEAAAAAKTSKSAKLTAGRAVKKAEEAEAKAKILENQNEQDAISSAETAATAAVAAV
        cdefgabcdefgabcdefg   abcdefgabcdefgabcdefgabcdefg
 421    AAVATAEAAATAGNEEEKKKEAEAEGKIKTLIQKVAEAIKAANQAKKAQIEAEIAVEVAKIEEHSEVAQK
                     abcdefgabcdefgabcdefgabcdefgabcdefg         abcdefgabcdef
 491    EVEEAEKANAKAKQAASEAQEAKTQTEKAAKAAEMVKAKDLAKTEVEIATKAEKEVADAKMEADEESSEA
        gabcdefgabcdefgabcdefgabcdefgabcdefg     abcdefgabcdefgabcdefg
 561    VEKAHAIKMQLEVAINAEKKAAKEVDITKLEDAKKEAQDATKNAKELLSIVQKASTLAIAKSKNAENSLE
        abcdefgabcdefgabcdefg        abcdefgabcdefgabcdefgabcdefg    abcdefg
 631    KVKKAAEVAKAKLAKAEAKNQAEEAKAIAAKADELSKGTKASTAEKTKAEAAAKEAAAQATKAADEAGKA
               abcdefgabcdefgabcdefgabcdefgabcdefg              abcdefgabcdefg
 701    ATAADNTKKAATSAEAKVEANKAVAAAKEAIIAKIKAAHEFLKTKNQDILEPVEMFSGGTENVTDREEQV
        abcdefgabcdefg
 771    KDEVEEQGDGSNEKAEEEEEEDSESALPNGSDGLEDDVDLDVDDDVEEEENVATNHQKGGKYEKGKVTDA
 841    VDDTNADKQFGDEFDAYNDIKKVTEALVKTMTSLVSEDPSVGDTINEFLSDMNQLFLSW
```

PvMSP3.7

```
   1   MKSNLKSTLYIIFLYWYVHIKDEQKWVTVNAETPNLRNGWSIKNGDIHNGENNLNRKYADYLNDTVEGNS
  71   KMEALEEGLSGQSEEAVVKAQQEDGEVQAQQEEAVDLQLADKQAAEKAAGEAQQFAKEATQAAEQAAEDA
                                         abcdefgabcdefgabcdefg   abcdefgabcde
 141   KKAAQAEKDAKAAQDEAKEKVKNAEKINEAVEQATSAKEQAQKASGEAETAKEKAAAATKAAEQAAAAAA
       fgabcdefgabcdefg       abcdefg          abcdefgabcdefgabcdefgabcdefgab
 211   AKTTEEAKTNKQQAMDQKGLAEEALKRAKEAATNAQQAKENAEIALERTKAEVSKELAEKEVLAAKEAQK
       cdefg            abcdefgabcdefgabcdefgabcdefgabcdefg
 281   EAEAAKNKAKAASDKVTSENEAVNKATLASKRADEALGRAQEYATIAEKAAGEAQKAETVQTAQVASKKA
        abcdefgabcdefgabcdefgabcdefg          abcdefgabcdefg               ab
 351   KDAETSANYERIIAELAAEVAKAEAEKNEAEKELKEANKADEEAKAVGAAIGADKAKEKAKIEAEKAKKQ
       cdefgabcdefgabcdefgabcdefgabcdefgabcdefgabcdefg  abcdefgabcdefgabcdefg
 421   LDEAKKLAKEVSSAEYNVEEKSVSEAKEKVSEIQKAVTAAKTAKELAVKAAKVATHILNAEKAKTEAQQA
       abcdefgabcdefg abcdefgabcdefgabcdefgabcdefgabcdefg      abcdefgabcdefg
 491   KKDALKAKAEAEKYASDSKAEKAKEAATKAATAADTATDKATKAEKESADAVTEANVNPDEIPDKEEEVK
       abcdefgabcdefg     abcdefgabcdefgabcdefgabcdefgabcdefg               a
 561   KKATQAADFSKEAVKAKNEALIEAEVAKAEAAKEEANMAQIEAGKAKAEAEKAAQTAPLSAVTEAKKKAE
       bcdefgabcdefgabcdefg     abcdefgabcdefgabcdefg            abcdefgabcde
 631   NMATLAKEKAETAANEAKGTNESADSIRDKAKEEVTEEVTNKITEIKGKAENASKAANEAKKAQTEAELA
       fg    abcdefgabcdefgabcdefg        abcdefgabcdefgabcdefgabcdefg     ab
 701   AAVAKAAVAKHEAKKAQELADDAKKKADEEAKPVESKAKDEAASAAEKAKENAKTAEDAADEVLLKAKDA
       cdefgabcdefgabcdefgabcdefg            abcdefgabcdefgabcdefgabcdefgabcd
 771   EEEVKNNWDNQQKKADITSKVNHVKEKTSQSIDAAKAAKKAKDLAEIALNVVKAEVANDKAKKAVTTAEA
       efgabcdefg                                 abcdefgabcdefgabcdefgabcdef
 841   AKDEAKKAAASAEQPKKEAESAVEDAETAKDEEKEVEKKAQEVAENANAAQEQLKIAEQELQTAKGATSM
       gabcdefg       abcdefgabcdefg   abcdefgabcdefgabcdefgabcdefgabcdefg
 911   ETLVQAKTKALAAAEDAVKKGQAAEDAANEAKNNAAKAAEAAEKAKKAAEESALKKKLKVLEIVKKYSKE
          abcdefgabcdefg          abcdefgabcdefgabcdefg    abcdefgabcdefg
 981   GYNTVDSDEHVLNEVEEQASEAKEEEEEDDEEEAEHSLPNDVEIEDDDDEGEEEEKEEEETEVKEEEDVK
1051   EEGHAQSSAHQSSVTELENQKAQSQEKSEKPLSDSNAQALLEDNYKDVVNFKKMADGLTKNIISTIDGDT
             abcdefgabcdefgabcdefg
1121   DVLDTLKDFAEDLNQFIFSM
```

PvMSP3.8

```
   1   MKHLSGIPLLVLFLNLYVIQNNVVSNEIVNLKNPNLRNGWAGKDVTLMDEQSGLDAKDGDEVNANEDQSE
  71   LLHKSDEGSLQTQGQNNEVVEESADVKKAKKAKADAEQAEAEAQKAKQKILDAEKETEKAKKEIKDAINK
                                   abcdefgabcdefgabcdefgabcdefgabcdefgabcdefg
 141   VKEYASSKESQVKKKVEEAKSAADEATKGSTKENTEQKAKAAEAALGEAQNAKVQMEKAAAIVDEVVKAM
       abcdefgabcdefg                 abcdefgabcdefgabcdefgabcdefg         ab
 211   EAEKEAQKAKEEAQKANEEAQNAKNKFVMIKPKQAGAGSPESKAEQEAKIANDKTTEAEQKAEQASAKAT
       cdefgabcdefgabcdefgabcdefg              abcdefgabcdefgabcdefgabcdefgab
 281   DAAQKATEAAQKAIEVAKKATQEKTGEEKEIEQENVTKIKEIASNAVKDAKDAKKAKREAQIKAEIVKLE
       cdefgabcdefg    abcdefgabcdefg     abcdefgabcdefgabcdefg  abcdefgabcde
 351   LAKEEAKKAVESAKKAKDEAAAAAKTSKSAKLAATRAAKKAEEAETKANLLENKNKQGPISLAATAEAAE
       fgabcdefgabcdefgabcdefg          abcdefgabcdefgabcdefg
 421   KEASTAVAAVATAEAAEKAKTEEVEKKEAEAEEKIKTLIQKVAKAIKAANQAKKAQIEAEIAVEVAKIEE
                    abcdefgabcdefgabcdefgabcdefgabcdefgabcdefg         abcdef
 491   HSEVAQKEVEEAEKANAKAKQAASEAQEAKTQTEKAAKAAEMVKAKDLAKTEVEIATKAEKEVADAKMEA
       gabcdefgabcdefgabcdefgabcdefgabcdefgabcdefg     abcdefgabcdefgabcdefg
 561   DEESSEAVEKAHAIKMQLEVAINAEKKAAKEVDITKLEDAKKEAQDATKNAKELLSIVQQASMLAIAKSQ
              abcdefgabcdefgabcdefg        abcdefgabcdefgabcdefgabcdefg
 631   DALNSLEKVKKAAEVAKAKLAKAEAKNQAEEAKAIAAKADELSKATKASTAEKTKAEAAAKEAAAQATKA
                     abcdefgabcdefgabcdefgabcdefgabcdefg              abcdefg
 701   ADEAGKAATAADNTKKAATSAEAKVEANKAVAAAKEAIIAKIKAAHEFLKTKNQDILEPVEMFSGGTENV
       abcdefgabcdefgabcdefg
 771   TDREEQVKEEVEEQGDGNNEQAEEAEEVDESESALPNGSDGLEDDVDFDVDDGEEEEENVATNKQKGGKY
 841   EKGKVTDAVDDTNADKQFGDEFDAYNDIKKVTEALVKTMTSLVSEDPSVGDTINEFLSDMNQLFLSW
```

PvMSP3.9

```
   1   MKSNLKSTLYIIFLYWYVHIKDEQKWVTVNAETPNLRNGWSIKNGDIHNGENNLNRKYADYLNDTVEGNS
  71   KMDAIEEGLSGQSEDAIVKAQQEDGEVEGQQDEAALQSEDEKEAENAAEEAQKFATQAQGAAEQAAQAAQ
                                               abcdefgabcdefgabcdefg   abcdef
 141   AAQDEAKKITENTEKIEEAVKQATDAKEEAENESREANNAKEEADAARKAKENKEDAVNQKKIAQAALE
       gabcdefgabcdefgabcdefgabcdefgabcdefgabcdefgabcdefg    abcdefgabcdefgab
 211   RAKTAATKAQTAKGKAEKALETTKAEVAKELAAKEAREAEKTRAVEEAQQIAKQAEEQLKTATKATQEAA
       cdefgabcdefgabcdefgabcdefg    abcdefgabcdefgabcdefgabcdefgabcdefgabcde
 281   QAAQAAQDEAKKITENTEKIEEAVKQATDAKEEAENESREANNAKEEADAARKAKENKEDAVNQKKIAQ
       fg   abcdefgabcdefgabcdefgabcdefgabcdefgabcdefgabcdefg    abcdefgabcde
 351   SALDKATNAATNAQKAKEKAEIALERTKAEVSKELAKKEVLEAEAAQKEAKDISDKMTIANKPVNKANLA
       fgabcdefgabcdefgabcdefgabcdefgabcdefgabcdefg                   abcdefg
 421   SKRAEEALEKAKKHVATAESATEEAKGANAVEKAKEASTKAKEAEKNAKNERIKAQLAAEVAKAEAVKDE
       abcdefgabcdefg     abcdefgabcdefgabcdefg      abcdefgabcdefgabcdefgabc
 491   AEKESKAAMDARRQAEAVKTANGAENAKKKAEIEAGKAKGHLKKAEELAKEVSSAEYEVTEDSVTKAKKK
       defgabcdefgabcdefg  abcdefgabcdefgabcdefgabcdefg        abcdefgabcdefg
 561   VSEAQEEAKAAKSAKELAVKAAEVATHILNAENAKKEAQQAKQAALQAKTDAKKYADQSNAEKAKEAATK
       abcdefgabcdefgabcdefg      abcdefgabcdefgabcdefgabcdefg     abcdefgabc
 631   AEAAATIATDKAKEAENEAEAAVAEAKVDPEQIPKKEEEVKKKATQAAVFSEDAKKAKNEALIEAEVAKA
       defgabcdefgabcdefgabcdefg                         abcdefg         abcd
 701   EAAKEEAKKAHIAAGKAKEEAEKAAQTAQLSEVTDAKSKAESMTTLAKEKADTAEKEANETNESAKSIQN
       efgabcdefgabcdefgabcdefg            abcdefgabcdefgabcdefgabcdefgabcdef
 771   EAKTEKPEDITKKIEDLKGKAEKVSNSAKEAKKAQTKAELAAAVAKAAVAKDEAKKAQDAADDAKNKADE
       g            abcdefgabcdefgabcdefg     abcdefgabcdefgabcdefgabcdefgabc
 841   AAKSVESKAKGEAASAAAKAKENATVAASAANEVSSKTKEAEEEIEKNWGDEKKKEEITSKVNHVKQETS
       defgabcdefg
 911   KSINAAEGAKRAKEVAELALKVVSAEVENDKAKKAVTTAEAAKDEAKKAVESAEKSKGEAESAVEDAETA
                     abcdefgabcdefgabcdefgabcdefgabcdefgabcdefgabcdefgabcdefg
 981   KEEEKEVEKKAQEASENANEAQAQLKIAEEELKKAKEADNEEKLQSAKTKALEAVDEAVKKGQAAEAAAN
                 abcdefgabcdefgabcdefgabcdefgabcdefg  abcdefgabcdefgabcdefgab
1051   EAKNKAAKATQSAEKAQKAAAESALKKKLNVLEIVKKYSKESYNTVDSDEHVLNEVEEQASEEKEEEEEE
       cdefgabcdefgabcdefgabcdefg
1121   EEAEHSVSNEVEIEDDEEEEEEEEEEGEETNTEGEKSEDNSPHSDTEPQEQNQSVKPPEQSEKTLNDSNT
1191   QALLSDNYKNVANFKKMADDLTKNIISTIDGDTGVIDTLKDFAEDLNQFIFSM
```

PvMSP3.10

```
   1   MKHTRSVTLYLFLLTLCAYLTGAEVIGGSPNNEAPNSSRHHLRNGFPGKNDSLPHEEPNNLEGKNESSDQ
  71   CDTINLGQVTEKEKKTIEQASVQAQDATKPEANNAEQIQAELQKVKTAKDESATAAKDAETAKKNAVDAG
                                            abcdefgabcdefgabcdefgabcdefgabcde
 141   KGLDAAKGAIKKAEEAAAEAKKQAGIAEKAEKDAEAAGKKDKLEDVNSQVQIAVEASTKAKDKKTEAEIA
       fgabcdefgabcdefgabcdefgabcdefgabcdefg
 211   VEIVKAVVAKEEAQKASDEAQKACEKAQKAHAKAQKASDTTKTVETFKTNAEAAAKNAKEKAGNANKAAT
         abcdefgabcdefgabcdefgabcdefgabcdefg          abcdefgabcdefgabcdefgab
 281   EAESANELSVAKQKAKDAEEAAKEAKKEQVKAEIAAEVAKAKVAKEEADAAQKKAEAAKKIVDKIAQDTK
       cdefg         abcdefgabcdefgabcdefg     abcdefgabcdefgabcdefgabcdefg
 351   VPEAQREAKLATQTASKATEAATEAGKKAQEAEESSKEAEEKAETSDAVKGKADAAEKAAGEAKKASIET
              abcdefgabcdefgabcdefgabcdefgabcdefgabcdefgabcdefg   abcdefg
 421   EIAIEVAKAEVLNAEVKKTAQEAEKDATEAKEQAEKAKAAAEEAKTHGEKAEKVGESTKAHSDEAQQENK
            abcdefgabcdefgabcdefgabcdefgabcdefgabcdefgabcdefgabcdefgabcdefgab
 491   NAKDASEEAENRAVDALEEAYAVEAHLARTKNAAESAKSATDMSELEKAKEEAIDAANIAHQKWLKATQA
       cdefgabcdefgabcdefgabcdefgabcdefg

 561   ATIAKEKKEAAKVAAEKAQTAANVVKDKAAKAEAKKAETEAVKAAVEARAAAEEAKQEAAKVGASKEPQE
              abcdefgabcdefgabcdefgabcdefgabcdefgabcdefgabcdefg
 631   TKNKANVEAEATGNEAKKAEDAAEEAKEAAKKANEATDANVARSEADKAIAAAKKAKKAREKAAYGLLKT
        abcdefgabcdefgabcdefgabcdefgabcdefg    abcdefg     abcdefg
 701   KNQYVLEPLDISPESADNITSKEEQVKEEMEDQGDEDSNEAEVEEALPNGSGAQEEDVNLEMDDEEEVEE
 771   VEENVATNQQTGGKREKRNTNDTVDDTNADKQFGDEFDTYNDIKKVTEALVKSMTSLVSDDPSVGDTINE
 841   FLSDMNHLFLSW
```

PvMSP3.11

```
  1   MRQFVRIAIFISLLNLYLQKDGAVRGEILNVNKTNLRNGTPEEGVPSEGELPADAQEGKGGDSIKEVIKR
 71   ASEETAKFADEAKEAFKKAQSLAEEAKEKAAEAAKAVGAMNGEKDTPPTTEKAQRASQAASAAEQKSNEA
       abcdefgabcdefgabcdefgabcdefgabcdefgabcdefg          abcdefgabcdefgabcd
141   QAAVRTAKEQETKALEAAQVVKGLAEKAEPTTGDKDEAEKKRKEAEEALSAVKANLSAVEAAKVAAEINA
      efgabcdefgabcdefg                  abcdefgabcdefgabcdefgabcdefgabcdefga
211   AEAVEEANTVMKELAKVKATEAADAAKKDLLGAEVAEGSNAKAEAEAHEFLKTTNQHIQEFEEMPSQGGD
      bcdefgabcdefg
281   HVKDKNEEVKEEVKEEAKEEAKEEVLESRGEEEEQTLEVEDDVDEEEEDEEDDDDEEDEDDEEDKEDEED
351   DDEDDGGDDDSGDNDSDDDDNDGDDHDEDEDD
```
