# Supplementary material for: Plasmodium vivax Merozoite Surface Protein-3 (PvMSP3): Expression of an 11 Member Multigene Family in Blood-Stage Parasites
Source: PLoS One. 2013 May 23;8(5):e63888. doi: 10.1371/journal.pone.0063888 (PMC3662707; doi:10.1371/journal.pone.0063888)
Supplement: Figure S2 — The typical tandemly-repeated heptad motif AXXAXXX, predicted to form α-helical coiled-coil structures, was found in the putative MSP3 proteins analyzed (see sequence IDs in Table S2) using the MEME algorithm (http://meme.sdsc.edu/meme4/cgi-bin/meme.cgi). Numbers in parentheses refers to amino acid position. (PDF) [file pone.0063888.s002.pdf]

Figure S2. The typical tandemly-repeated heptad motif AXXAXXX, predicted to form α-helical coiled-coil structures, was found in the putative MSP3 proteins analyzed (see sequence IDs in Table S2) using the MEME algorithm (http://meme.sdsc.edu/meme4/cgi-bin/meme.cgi). Numbers in parentheses refers to amino acid position.

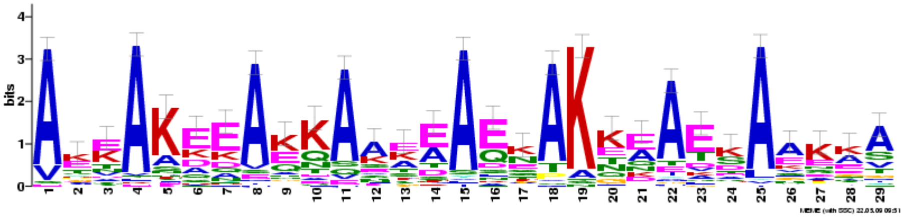

	AXXAXXXAXXAXXXAXXAXXXAXXAXXX
PfMSP3.1-FC27 (94)	AKQASQDAEQAAKDAENASKEAEEAAKEA
PfMSP3.1-FC27 (138)	ACTAASKAKKAVETALKAKDDAETALKTS
PfMSP3.1-CSL2 (94)	AKQASQDAEQAAKDAEQAAKDAEQASKDA
PfMSP3.1-CSL2 (138)	ACTAASKAKKAFETASNAKKAAESALKTN
PfMSP3.1-CSL2 (178)	SRKTKEYAEQVEKDYERAKNAYQKANQAV
$PkMSP3_{140}$ (202)	AENAVQEIQKAKKEADIAKNAATKLMEES
$PkMSP3_{140}$ (501)	VPEVTEAVKSAKEEAENAKKEEEQAEMES
$PkMSP3_{172}$ (241)	AEKAKEEAENAVKEIQKAKEEADIAKKAA
PcyMSP3.10 (243)	ATEAKKQAEKAKEAAEEAKTQEKKAEKAG
PcyMSP3.10 (435)	AETARNEAKNAENAAEEAKQAAKKANDAT
PcyMSP3.10 (110)	AKTEKENLPDSANDAETAKNNVVDARNGL
PvMSP3.1 (220)	AYELKKEAEKAQEEAEAAKTEIEKLSKVY
PvMSP3.1 (453)	AKNAKDKATKAAKEAEEAKKQAEKAEKIT
PvMSP3.1 (593)	AIAADVLAQKASTEAQSLKQEAEKLAENI
PvMSP3.2 (227)	AEEAKKEAEKAQTEVEAAKKEAEKVMAEA
PvMSP3.2 (688)	EKVAKAEVDKATSEAEKAKTAAGEAKNAS
PvMSP3.3 (101)	AQNAKAEAEKAAKAAESAKENTLDALEKV
PvMSP3.3 (373)	VEKAKEAEKQAKSEAEKAKGMADDAGGKG
PvMSP3.4 (231)	AYEAKKKARESKVAAENAKNDATSAASGS
PvMSP3.4 (388)	VKSAKSEAVNQAAAADKAKKEAEAAANSA
PvMSP3.4 (510)	AQEAAQKAKEQEAEALKAKGNAQTASDQA
PvMSP3.4 (821)	AGVAKEDAKKAAASAEQPKKEAESAEEGA
PvMSP3.5 (223)	AYEAKRNANDSKEAAEKTKKDATSAVSDS
PvMSP3.5 (508)	AQEAAKRAQEQDVAASTAKGNAETAAKEA
PvMSP3.5 (619)	ADKASAAAKTADEKAASAKKEADDATTES
PvMSP3.5 (821)	AGVAKEDAKKAAASAEQPKKEEESAEEDA
PvMSP3.6 (170)	AKEKKETANTALGEAQKAKKEMETAAVIV
PvMSP3.6 (204)	AMEAEKESQKANEEAQKANQEAENAKNNI
PvMSP3.6 (260)	AKGAIENAKQASTRATGAAQKATGAAQKA
PvMSP3.6 (358)	ANKAKKEAAAAAKTSKSAKLTAGRAVKKA
PvMSP3.6 (494)	AEKANAKAKQAASEAQEAKTQTEKAAKAA
PvMSP3.6 (689)	ATKAADEAGKAATAADNTKKAATSAEAKV
PvMSP3.7 (173)	ATSAKEQAQKASGEAETAKEKAAAATKAA
PvMSP3.7 (837)	AEAAKDEAKKAAASAEQPKKEAESAVEDA
PvMSP3.8 (215)	AQKAKEEAQKANEEAQNAKNKFVMIKPKQ
PvMSP3.8 (362)	AKKAKDEAAAAAKTSKSAKLAATRAAKKA
PvMSP3.8 (501)	AEKANAKAKQAASEAQEAKTQTEKAAKAA
PvMSP3.8 (696)	ATKAADEAGKAATAADNTKKAATSAEAKV
PvMSP3.8 (98)	AKKAKADAEQAEAEAQKAKQKILDAEKET

PvMSP3.9 (113)	AENAAEEAQKFATQAQGAAEQAAQAAQAA
PvMSP3.9 (162)	ATDAKEEAENESREANNAKEEADAAARKA
PvMSP3.9 (306)	ATDAKEEAENESREANNAKEEADAAARKA
PvMSP3.9 (590)	AENAKKEAQQAKQAALQAKTDAKKYADQS
PvMSP3.9 (948)	AEAAKDEAKKAVESAEKSKGEAESAVEDA
PvMSP3.10 (114)	VKTAKDESATAAKDAETAKKNAVDAGKGL
PvMSP3.10 (350)	VPEAQREAKLATQTASKATEAATEAGKKA
PvMSP3.10 (446)	ATEAKEQAEKAKAAAEEAKTHGEKAEKVG
PvMSP3.10 (502)	AVDALEEAYAVEAHLARTKNAAESAKSAT
PvMSP3.11 (143)	VRTAKEQETKALEAAQVVKGLAEKAEPTT
PvMSP3.11 (78)	ADEAKEAFKKAQSLAEEAKEKAAEAAKAV
