# Supplementary material for: Plasmodium vivax Merozoite Surface Protein-3 (PvMSP3): Expression of an 11 Member Multigene Family in Blood-Stage Parasites
Source: PLoS One. 2013 May 23;8(5):e63888. doi: 10.1371/journal.pone.0063888 (PMC3662707; doi:10.1371/journal.pone.0063888)
Supplement: Figure S3 — Glutamate-rich motifs from the C-termini of MSP3 proteins. The high content of glutamic acid (E) residues are present amidst other acidic amino acids in clusters, resulting in hydrophilic C-terminal regions. (PDF) [file pone.0063888.s003.pdf]

Figure S3. Glutamate-rich motifs from the C-termini of MSP3 proteins. The high content of glutamic acid (E) residues are present amidst other acidic amino acids in clusters, resulting in hydrophilic C-terminal regions.

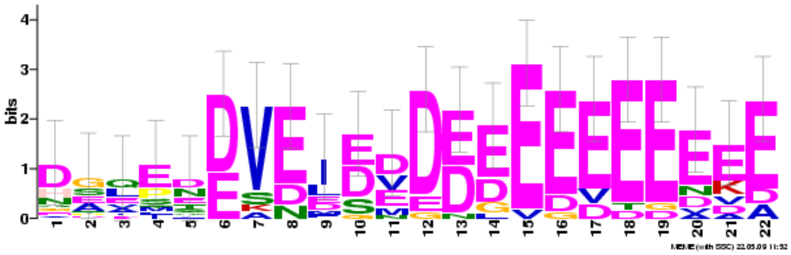

PfMSP3.1-FC27 (278)	NEEETESEISEDEEEEEEEEKE	1
PfMSP3.1-CSL2 (277)	NEEETESEISEDEEEEEEEEKE	1
PkMSP3$_{140}$ (773)	AGDMEDKELEEEEEEEEEEEDD	1
PkMSP3$_{172}$ (816)	DDVEDDVEDDNDDDEDEEEEEE	1
PcyMSP3.10 (548)	DGQEEDVNMEMDDGEEVDDEEE	1
PvMSP3.1 (741)	DAEPQEAEEGDEEDEEDTEEEE	1
PvMSP3.2 (793)	DAAPSDVEEDEGELEDEEEEEE	1
PvMSP3.3 (916)	QTQIDEVDISVDNEEEEEGAAE	1
PvMSP3.4 (996)	DSASSDVEIEDDEEEEEEEDEE	1
PvMSP3.5 (999)	HPILNDVEIDDDDEEEEEEEEE	1
PvMSP3.6 (801)	DGLEDDVDLDVDDDVEEEENVA	1
PvMSP3.7 (1015)	HSLPNDVEIEDDDDEGEEEEKE	1
PvMSP3.8 (809)	DGLEDDVDFDVDDGEEEEENVA	1
PvMSP3.9 (1124)	HSVSNEVEIEDDEEEEEEEEEE	1
PvMSP3.10 (751)	GAQEEDVNLEMDDEEEVEEVEE	1
PvMSP3.11 (312)	EEQTLEVEDDVDEEEEDEEDDD	1
