# Supplementary material for: Plasmodium vivax Merozoite Surface Protein-3 (PvMSP3): Expression of an 11 Member Multigene Family in Blood-Stage Parasites
Source: PLoS One. 2013 May 23;8(5):e63888. doi: 10.1371/journal.pone.0063888 (PMC3662707; doi:10.1371/journal.pone.0063888)
Supplement: Figure S4 — Immunofluorescence experiments showing the expression of the PvMSP3 proteins in relation to PvMSP1. Mixed stages of P. vivax (Sal 1) infected RBCs in air-dried thin smears were stained with a mixture of antibodies recognizing all PvMSP3 proteins (Green, Alexa 488). A monoclonal antibody (3F8.1A2) was used to detect PvMSP1 (Red, Alexa 555). Parasite nuclei were stained with DAPI (Blue) in ProLong® Gold antifade reagent. The slides were fixed with 0.25% paraformaldehyde in 1x DPBS at room temperature for 20 min, immediately upon removal from −80°C. 4A. Individual IFAs and the merged co-localization images are shown. 4B. different layers of one schizont were observed using a Zeiss LSM 510 META confocal microscope. The upper panel shows the merged pictures with the green (PvMSP3), red (PvMSP1), blue (parasite nuclei) and brightfield channels, and the lower panel shows the merged pictures without the brightfield channel. (PDF) [file pone.0063888.s004.pdf]

Figure S4. Immunofluorescence experiments showing the expression of the PvMSP3 proteins in relation to PvMSP1. Mixed stages of *P. vivax* (Sal 1) infected RBCs in air-dried thin smears were stained with a mixture of antibodies recognizing all PvMSP3 proteins (Green, Alexa 488). A monoclonal antibody (3F8.1A2) was used to detect PvMSP1 (Red, Alexa 555). Parasite nuclei were stained with DAPI (Blue) in ProLong® Gold antifade reagent. The slides were fixed with 0.25% paraformaldehyde in 1x DPBS at room temperature for 20 min, immediately upon removal from -80ºC. **4A**. Individual IFAs and the merged co-localization images are shown. **4B**. different layers of one schizont were observed using a Zeiss LSM 510 META confocal microscope. The upper panel shows the merged pictures with the green (PvMSP3), red (PvMSP1), blue (parasite nuclei) and brightfield channels, and the lower panel shows the merged pictures without the brightfield channel.

4A

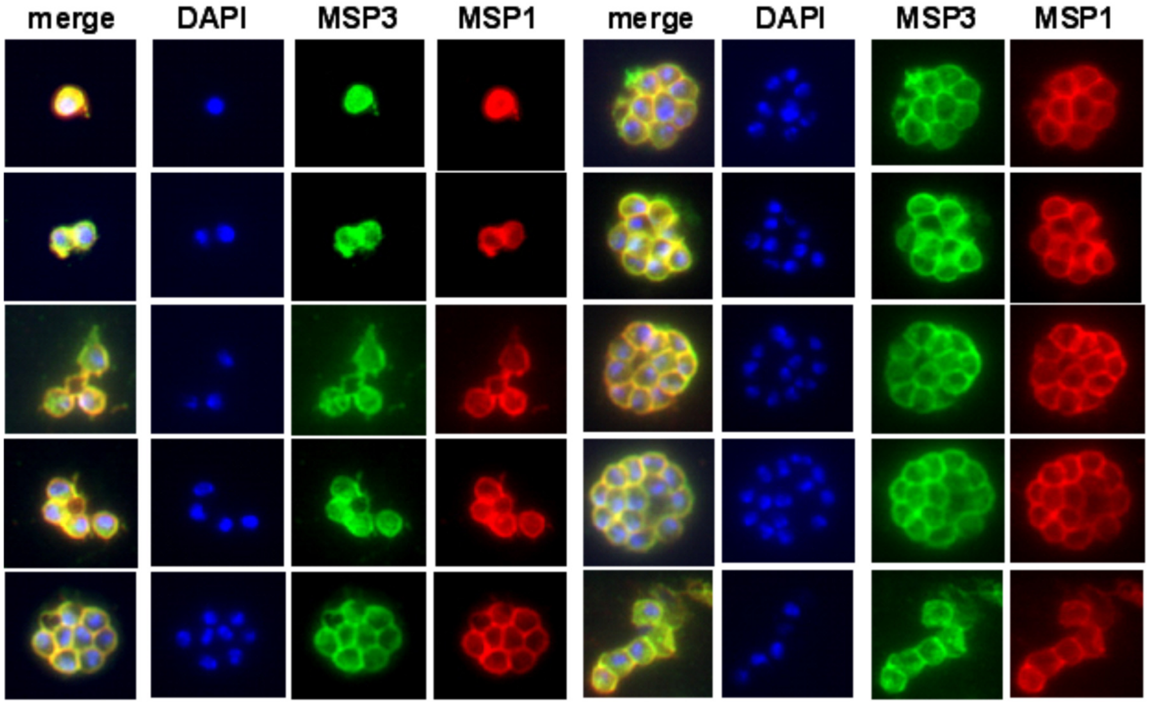

4B

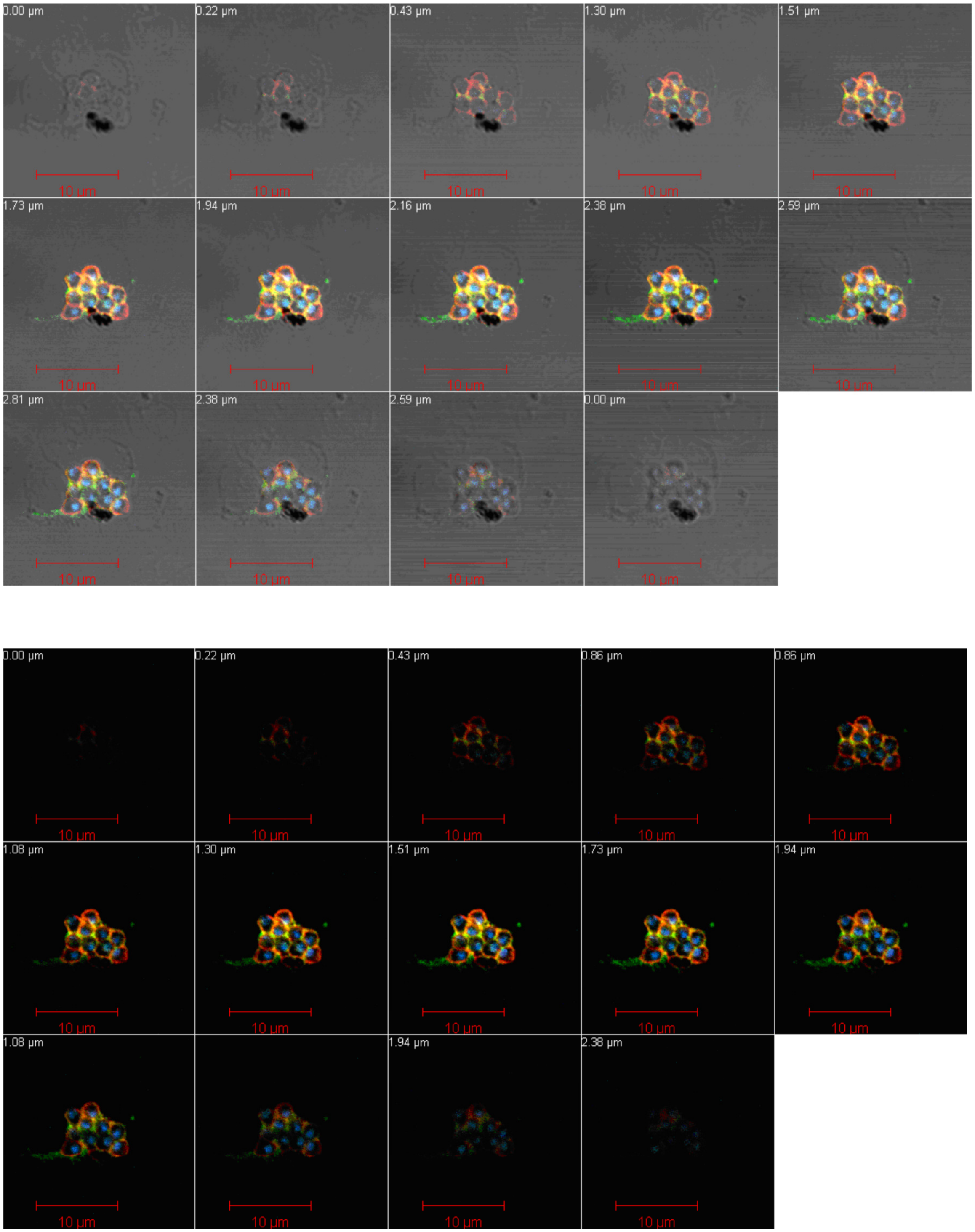
0.00 µm
0.22 µm
0.43 µm
1.30 µm
1.51 µm
1.73 µm
1.94 µm
2.16 µm
2.38 µm
2.59 µm
2.81 µm
2.38 µm
2.59 µm
0.00 µm
0.00 µm
0.22 µm
0.43 µm
0.86 µm
0.86 µm
1.08 µm
1.30 µm
1.51 µm
1.73 µm
1.94 µm
1.08 µm
1.94 µm
2.38 µm
10 µm
