# Supplementary material for: Plasmodium vivax Merozoite Surface Protein-3 (PvMSP3): Expression of an 11 Member Multigene Family in Blood-Stage Parasites
Source: PLoS One. 2013 May 23;8(5):e63888. doi: 10.1371/journal.pone.0063888 (PMC3662707; doi:10.1371/journal.pone.0063888)
Supplement: Figure S5 — Cross reactivity is not caused by recombinant protein expression vector residues or 6xHis tag. (PDF) [file pone.0063888.s005.pdf]

Figure S5

Cross reactivity is not caused by vector residues or 6xHis tag
(α-rPvMSP3.2)

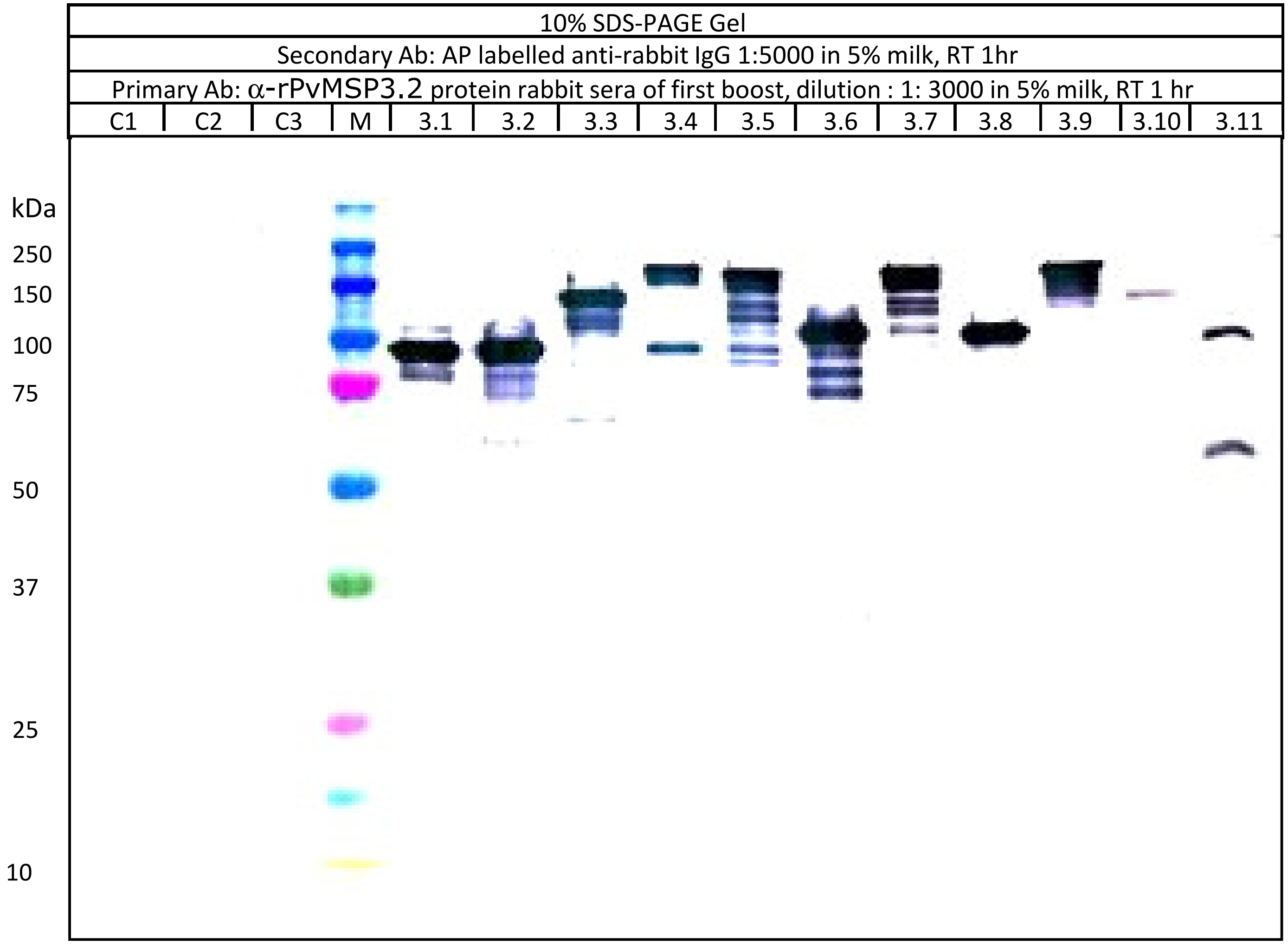

* C1-C3 = Control proteins in the same expression vector or contains 6x-His tag
