# Supplementary material for: Plasmodium vivax Merozoite Surface Protein-3 (PvMSP3): Expression of an 11 Member Multigene Family in Blood-Stage Parasites
Source: PLoS One. 2013 May 23;8(5):e63888. doi: 10.1371/journal.pone.0063888 (PMC3662707; doi:10.1371/journal.pone.0063888)

Figure S6

Cross-reactive antibodies present in the rPvMSP3 antiserum were removed by serial passage through affinity columns

Figure S6

rPvMSP3 proteins used for rabbit serum production and affinity gel coupling

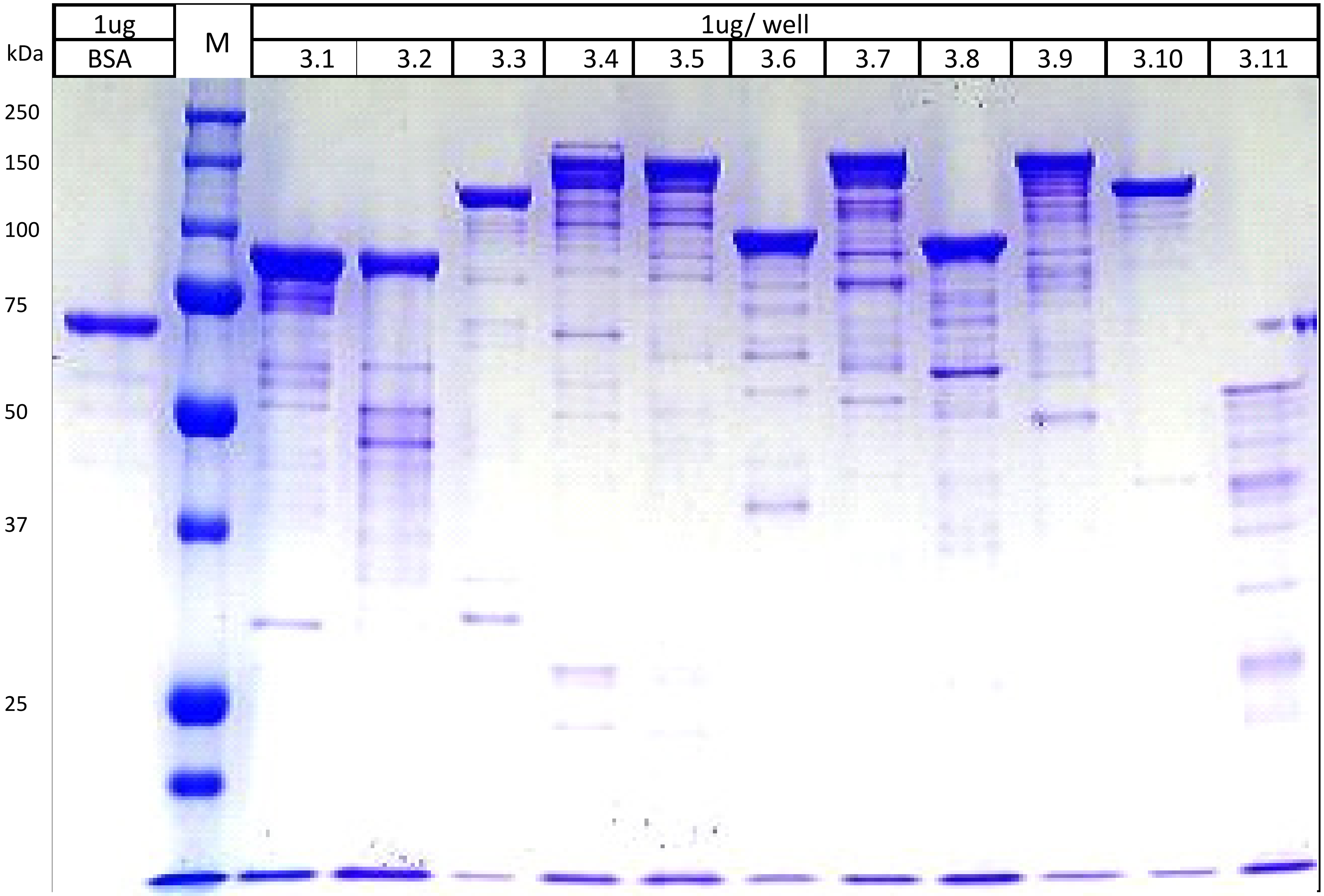

Figure S6

Immunoblot using α-rPvMSP3.1 Ab **before** affinity purification with rPvMSP3.2, rPvMSP3.8 and rPvMSP3.9 protein column

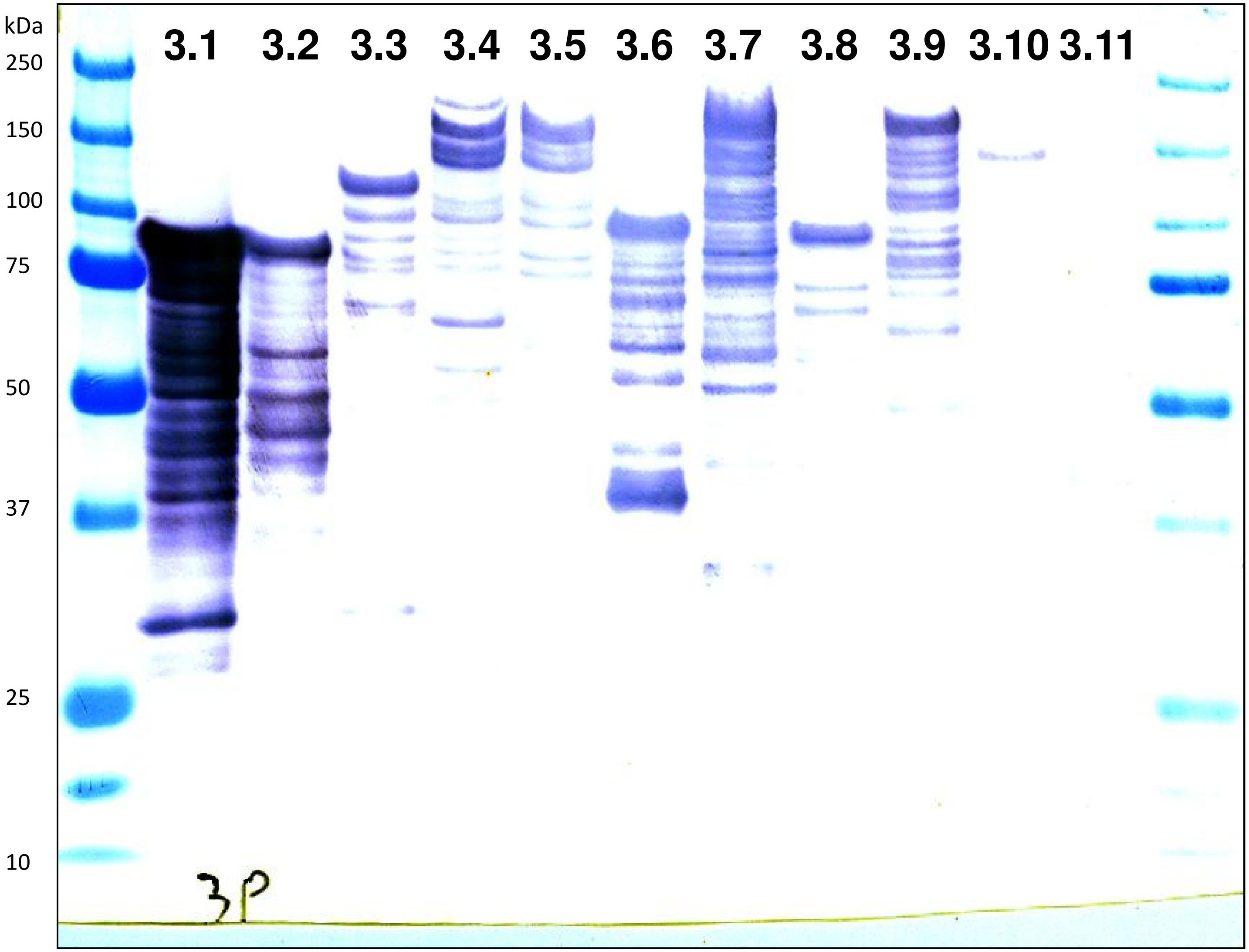

Figure S6

Immunoblot using α-rPvMSP3.1 Ab **after** affinity purification with rPvMSP3.2, rPvMSP3.8 and rPvMSP3.9 protein column

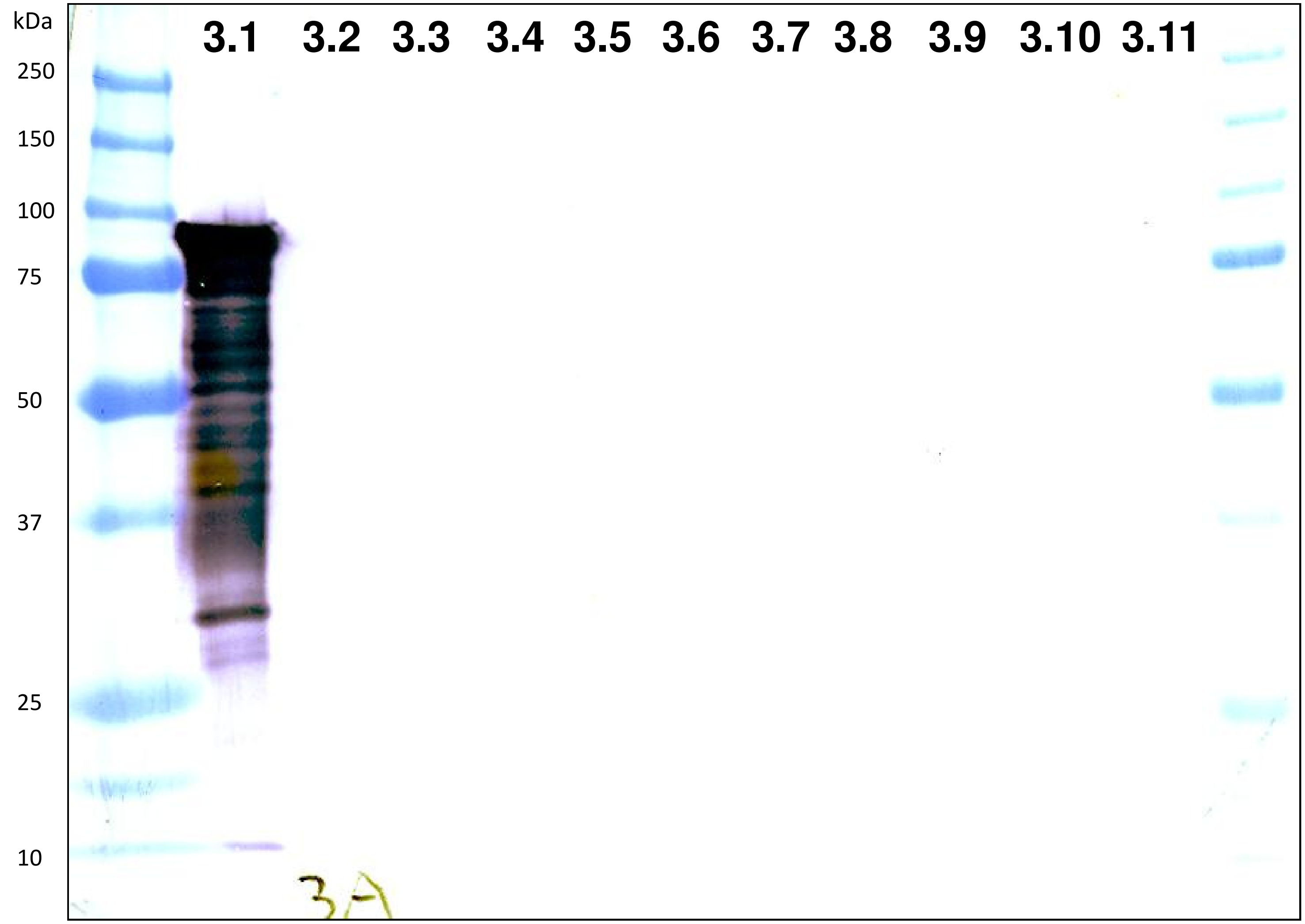

Figure S6

Immunoblot using α-rPvMSP3.2 Ab **before** affinity purification with rPvMSP3.1, rPvMSP3.6 and rPvMSP3.7 protein column

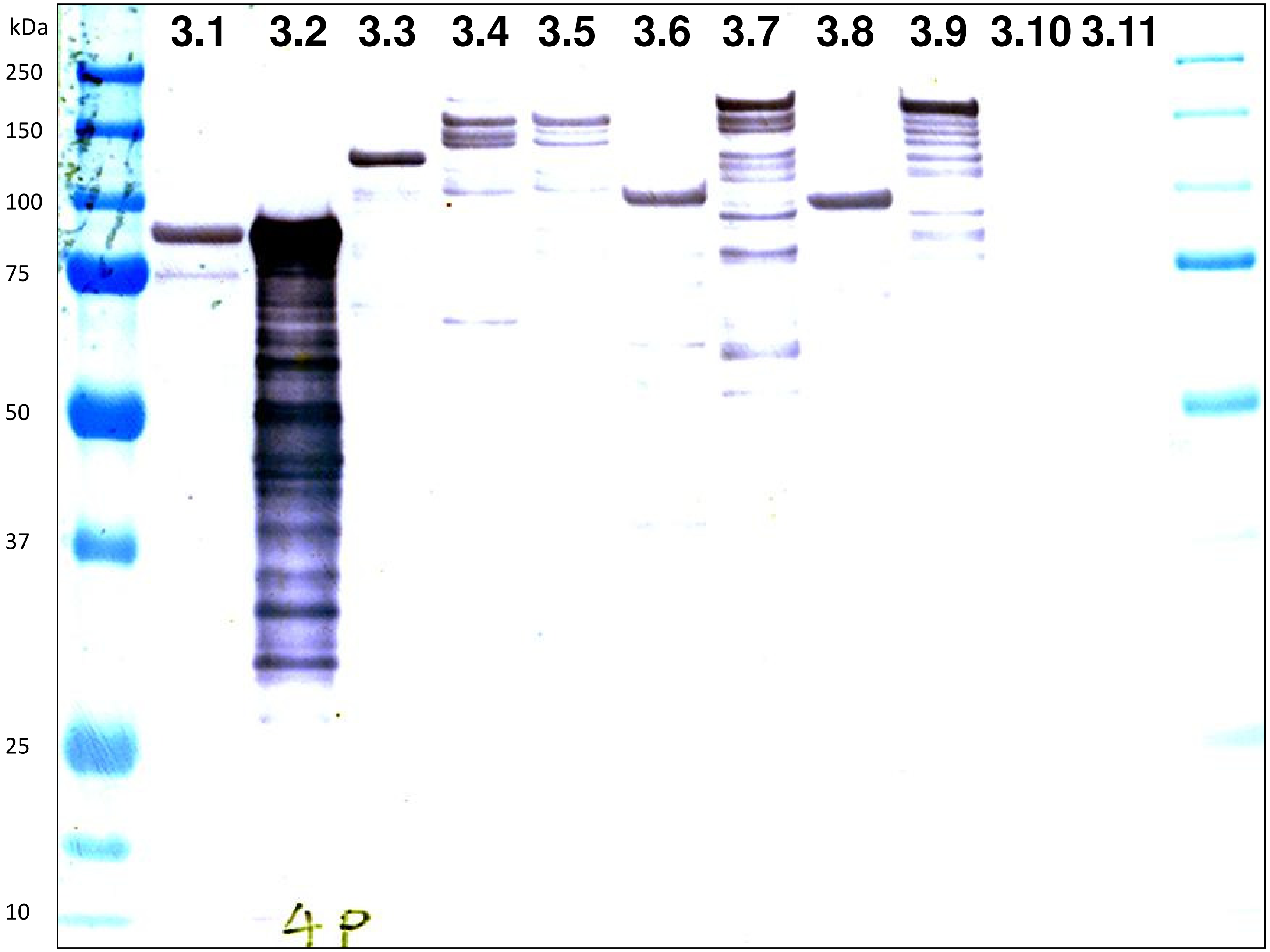

Figure S6

Immunoblot using α-rPvMSP3.2 Ab **after** affinity purification with rPvMSP3.1, rPvMSP3.6 and rPvMSP3.7 protein column

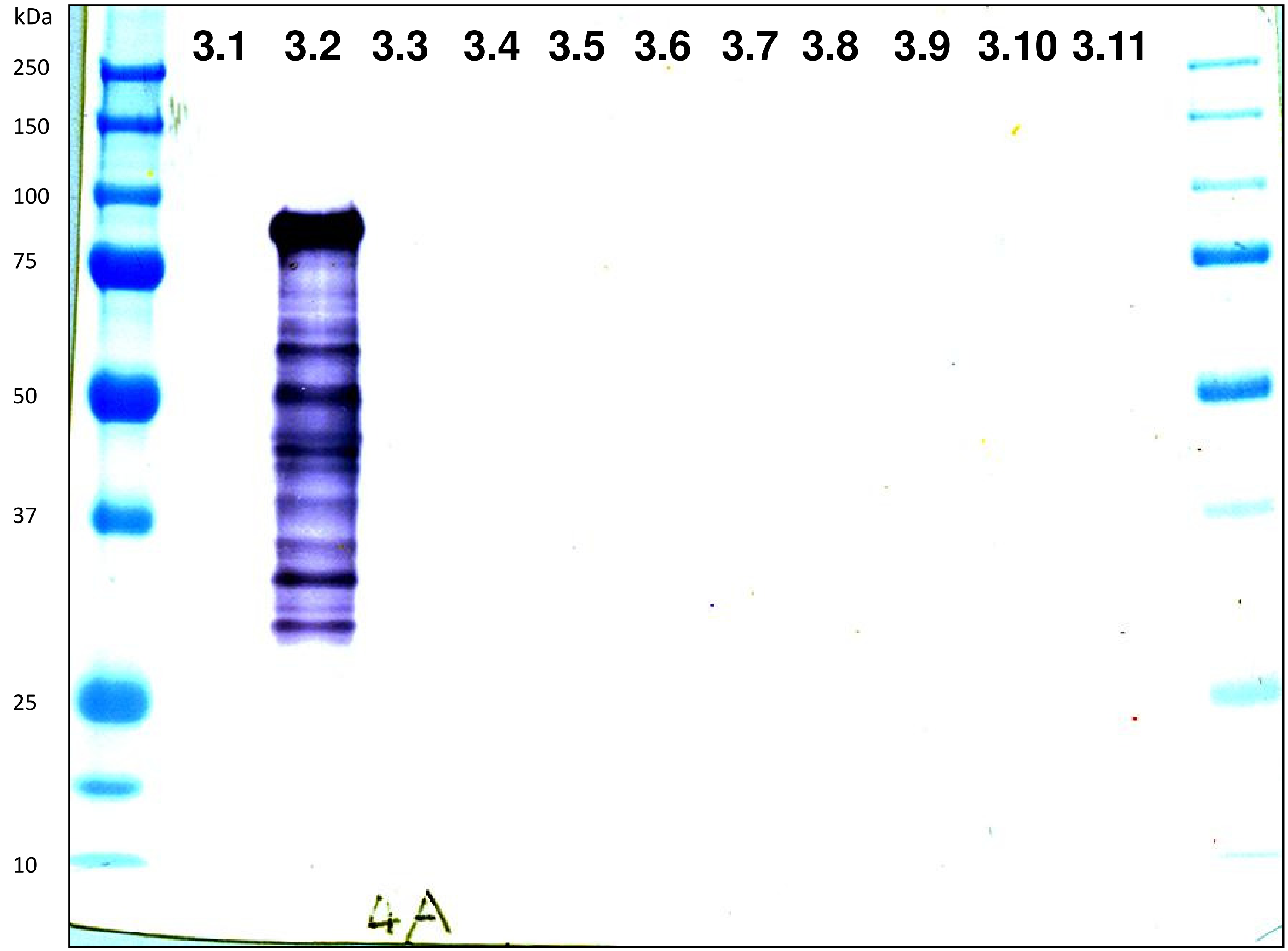

Figure S6

Immunoblot using α-rPvMSP3.3 Ab **before** affinity purification with rPvMSP3.5, rPvMSP3.7 and rPvMSP3.9 protein columns

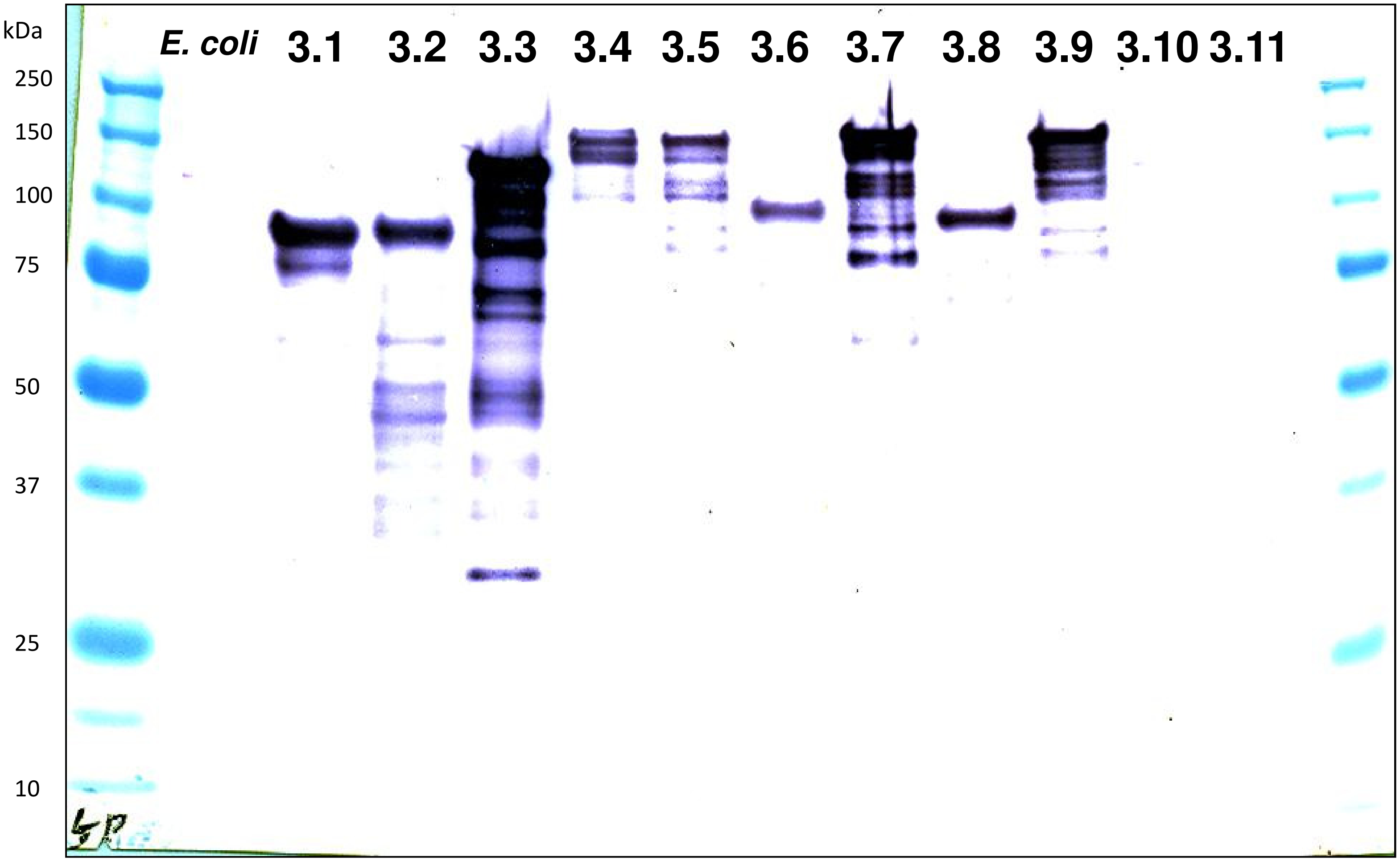

Figure S6

Immunoblot using α-rPvMSP3.3 Ab **after** affinity purification with rPvMSP3.5, rPvMSP3.7 and rPvMSP3.9 protein columns

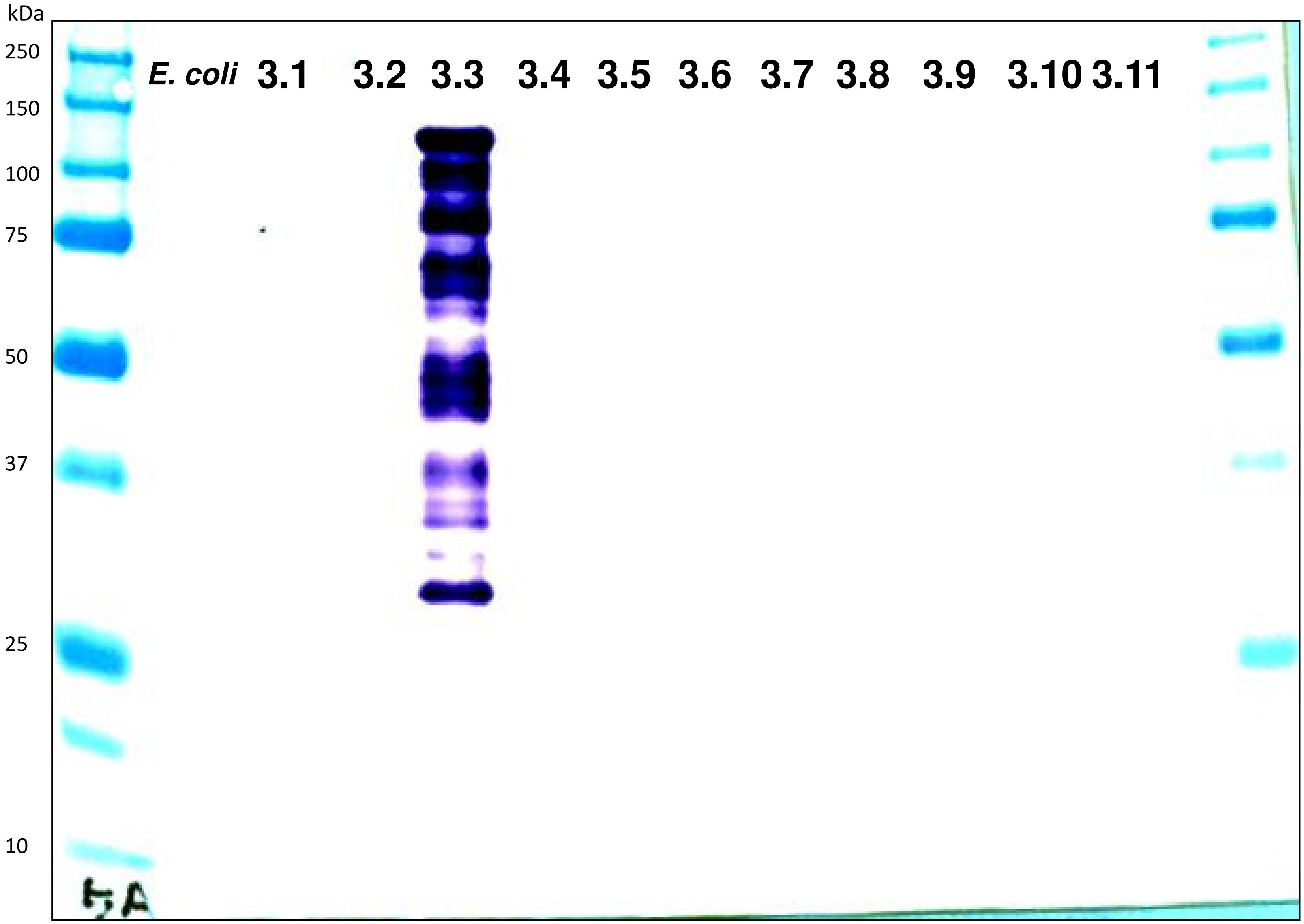

Figure S6

Immunoblot using α-rPvMSP3.4 Ab **before** affinity purification with rPvMSP3.3, rPvMSP3.6 and rPvMSP3.8 antigen columns

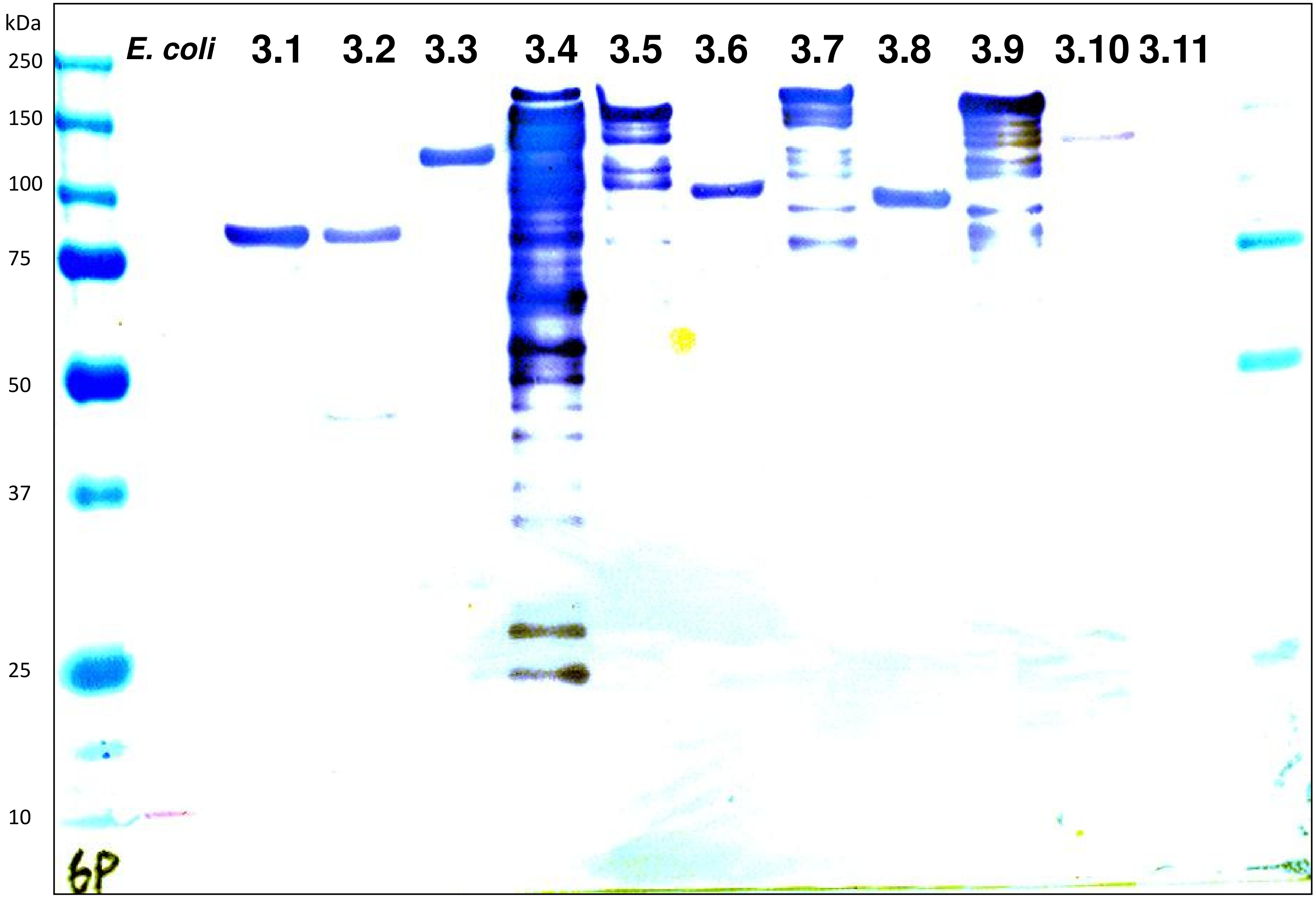

Figure S6

Immunoblot using α-rPvMSP3.4 Ab **after** affinity purification with rPvMSP3.3, rPvMSP3.6 and rPvMSP3.8 antigen columns

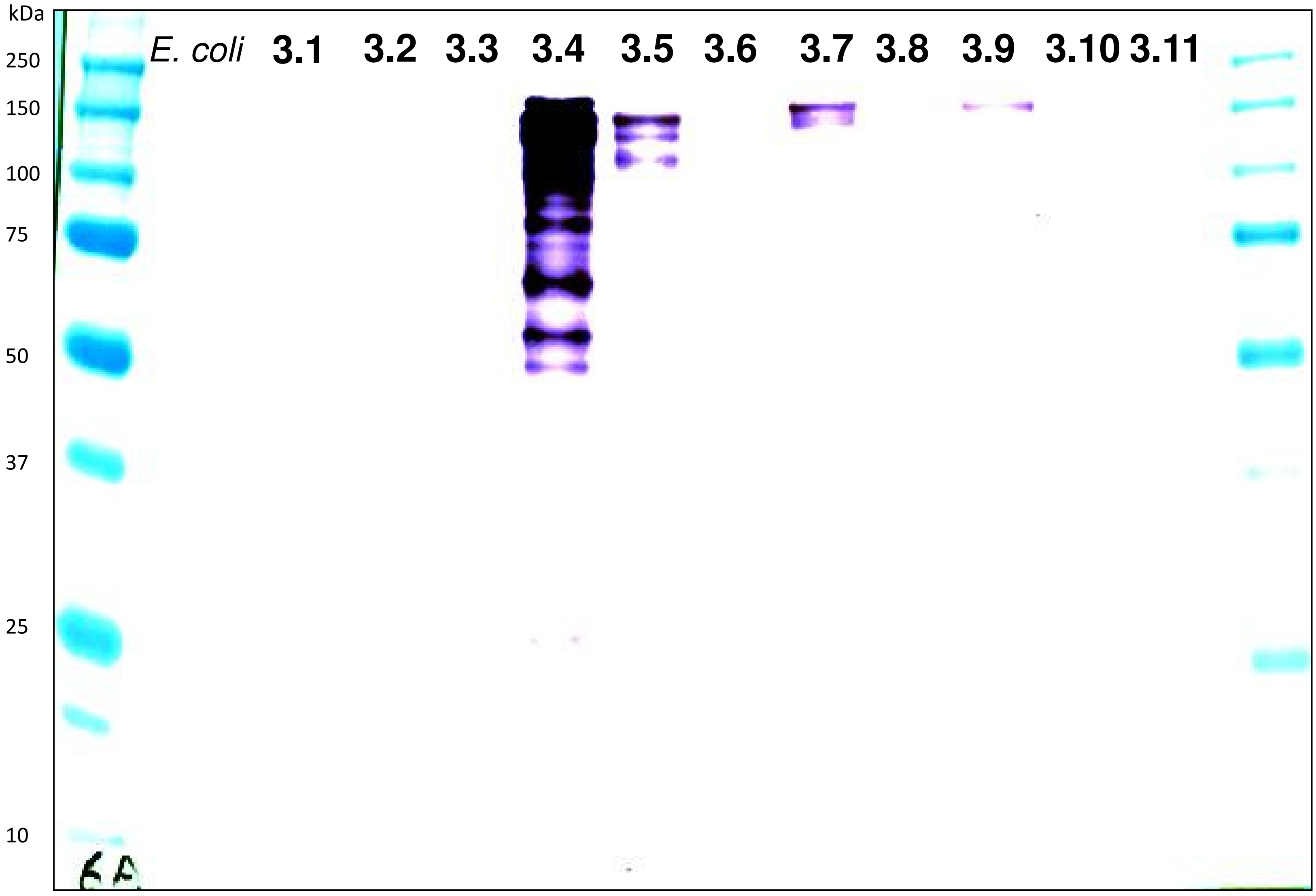

Figure S6

Immunoblot using α-rPvMSP3.4 Ab **after** secondary affinity purification with rPvMSP3.1, rPvMSP3.5, rPvMSP3.7 and rPvMSP3.9 antigen columns

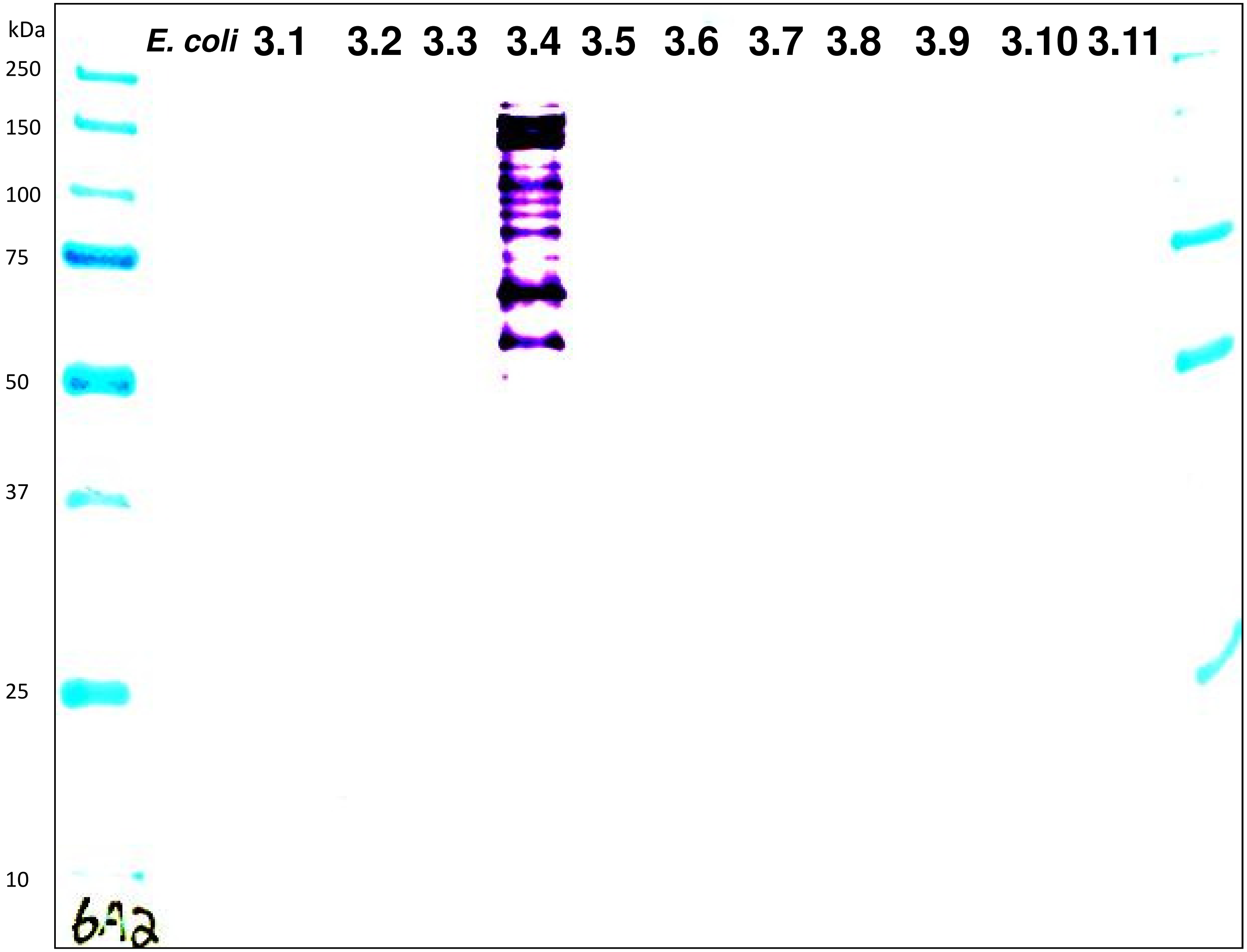

Figure S6

Immunoblot using α-rPvMSP3.5 Ab **before** affinity purification with rPvMSP3.1, rPvMSP3.2 and rPvMSP3.4 antigen columns

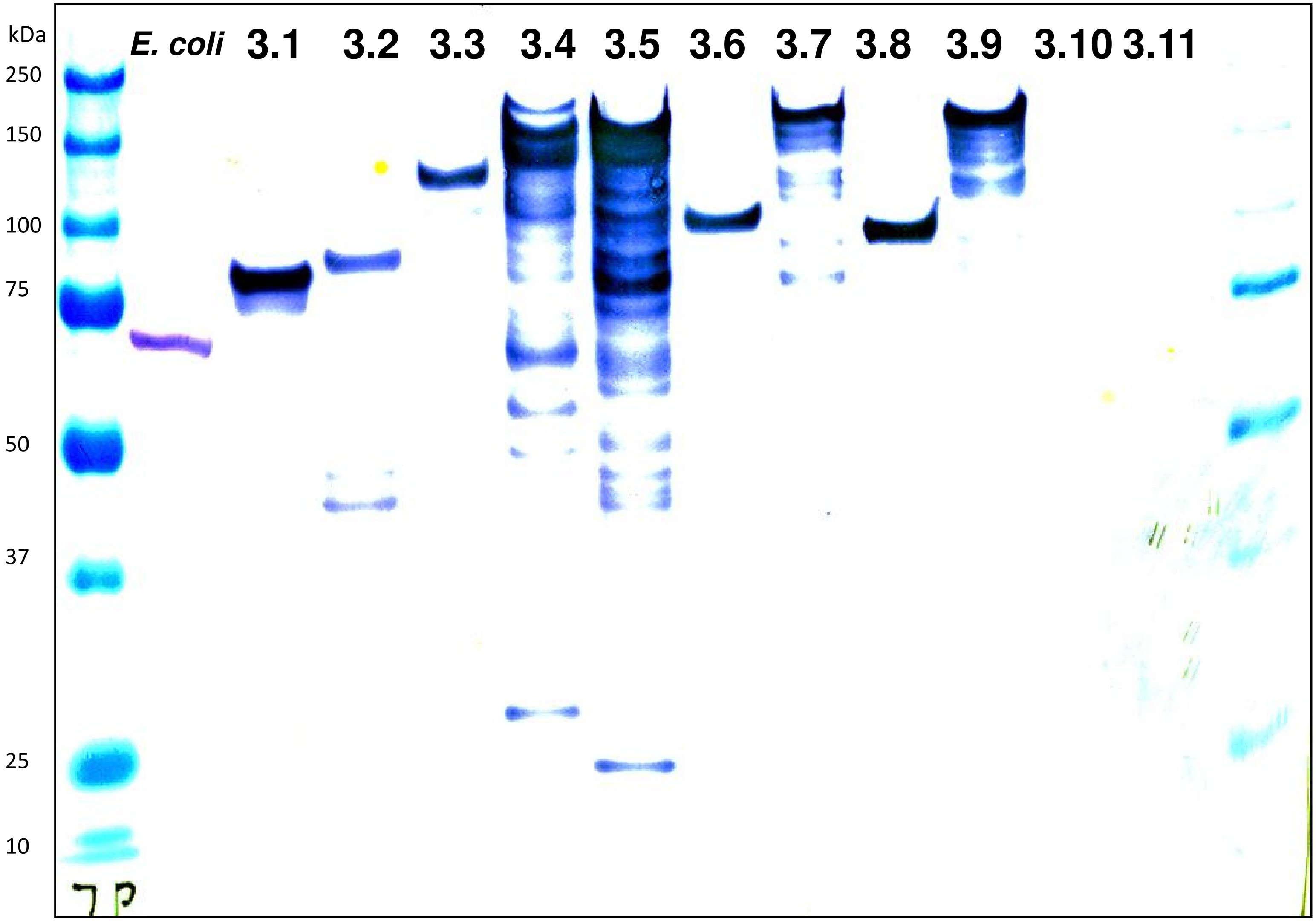

Figure S6

Immunoblot using α-rPvMSP3.5 Ab **after** affinity purification with rPvMSP3.1, rPvMSP3.2 and rPvMSP3.4 antigen columns

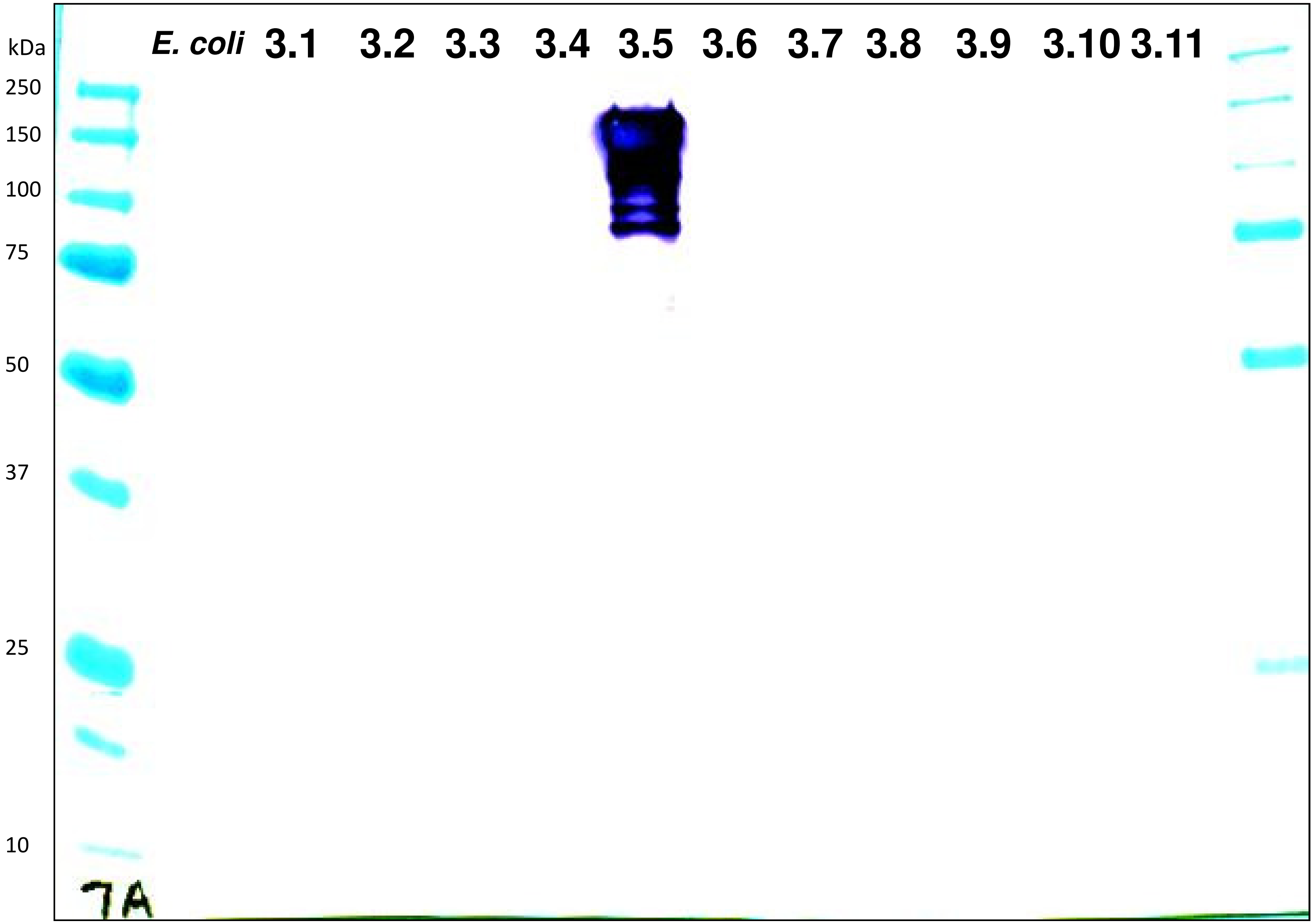

Figure S6

Immunoblot using α-rPvMSP3.6 Ab **before** affinity purification with rPvMSP3.1, rPvMSP3.7 and rPvMSP3.8 columns

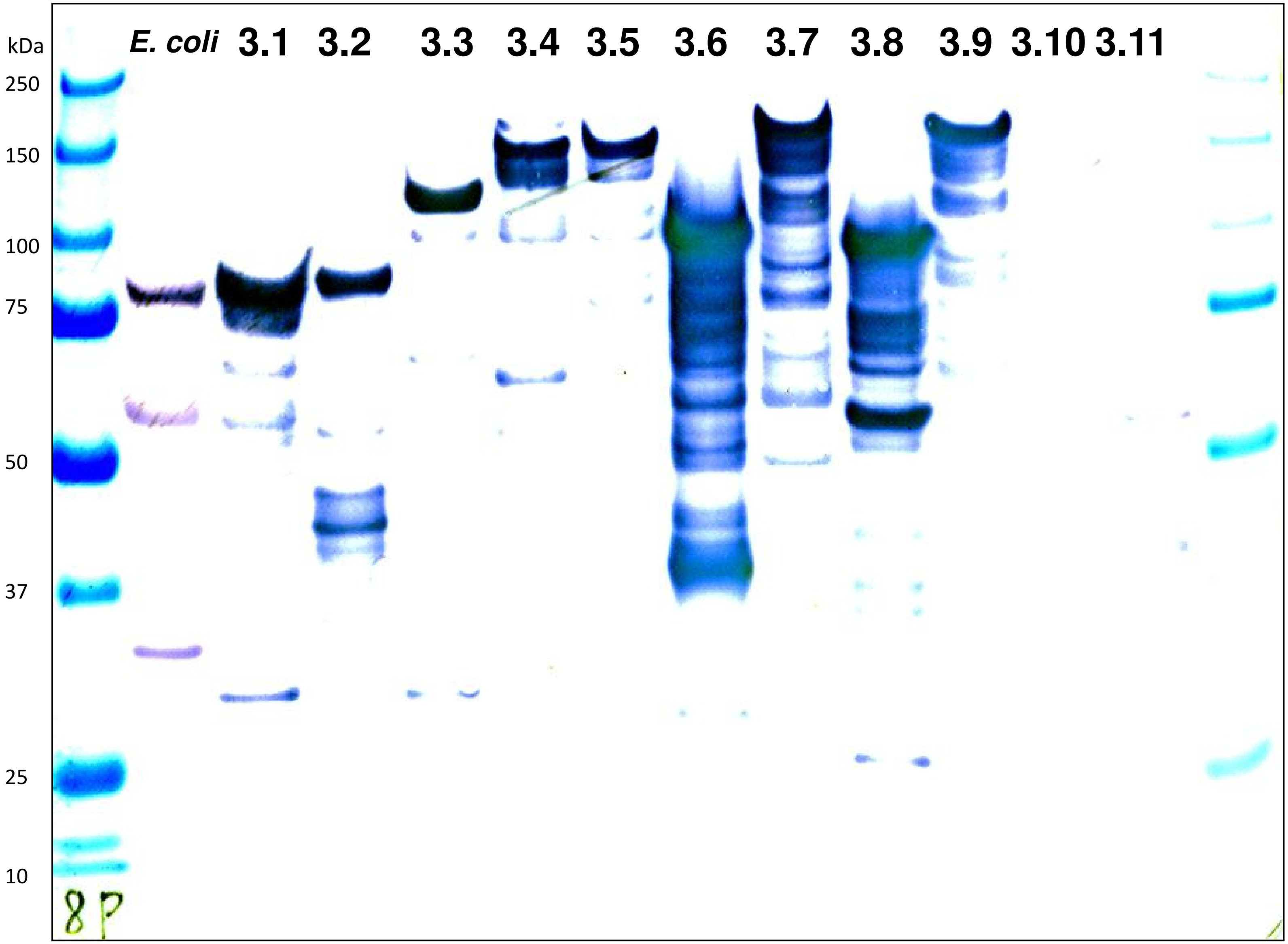

Figure S6

Immunoblot using α-rPvMSP3.6 Ab **after** affinity purification with rPvMSP3.1, rPvMSP3.7 and rPvMSP3.8 columns

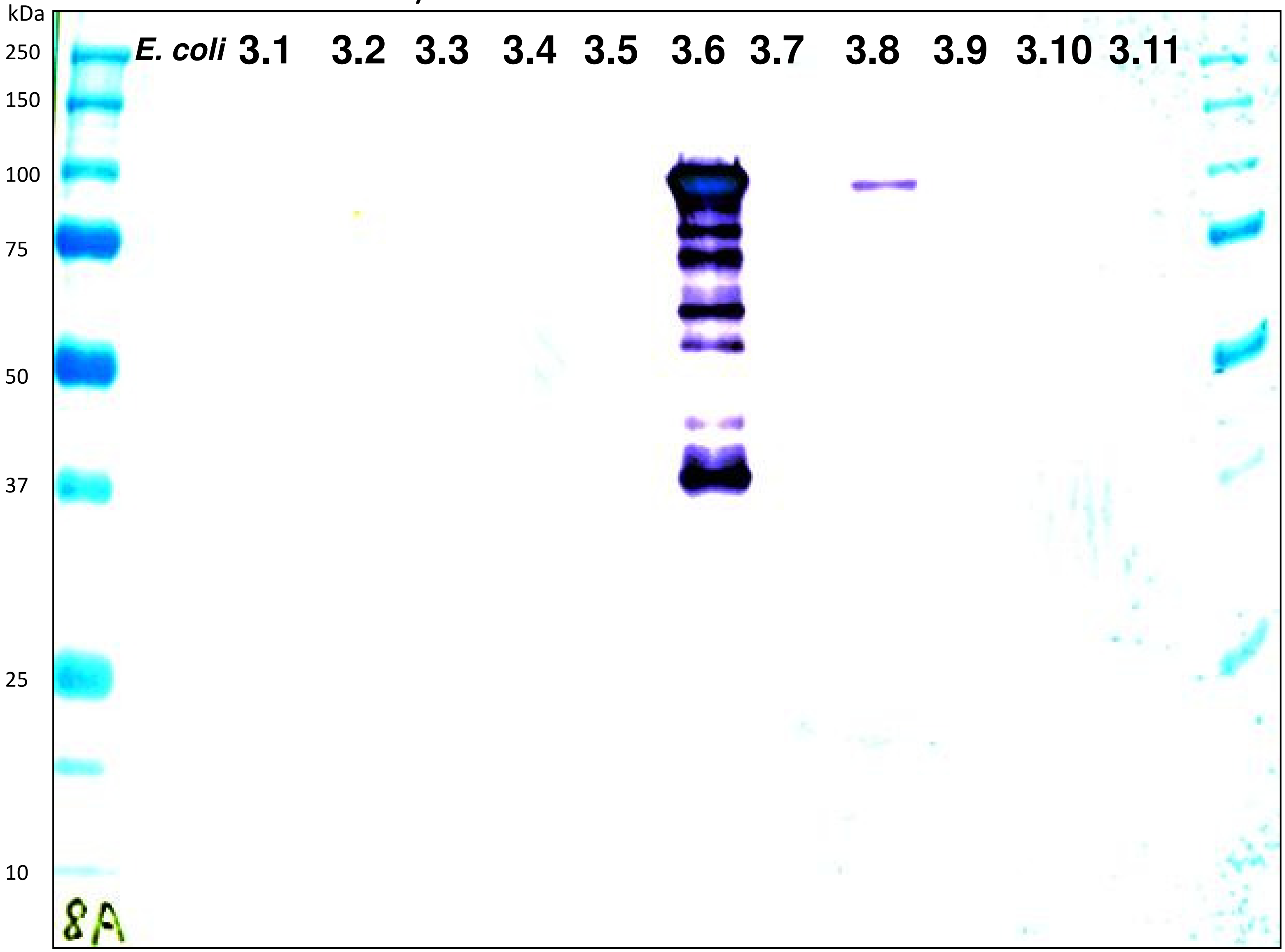

Figure S6

Immunoblot using α-rPvMSP3.7 Ab **before** affinity purification with rPvMSP3.4, rPvMSP3.5 and rPvMSP3.9 columns

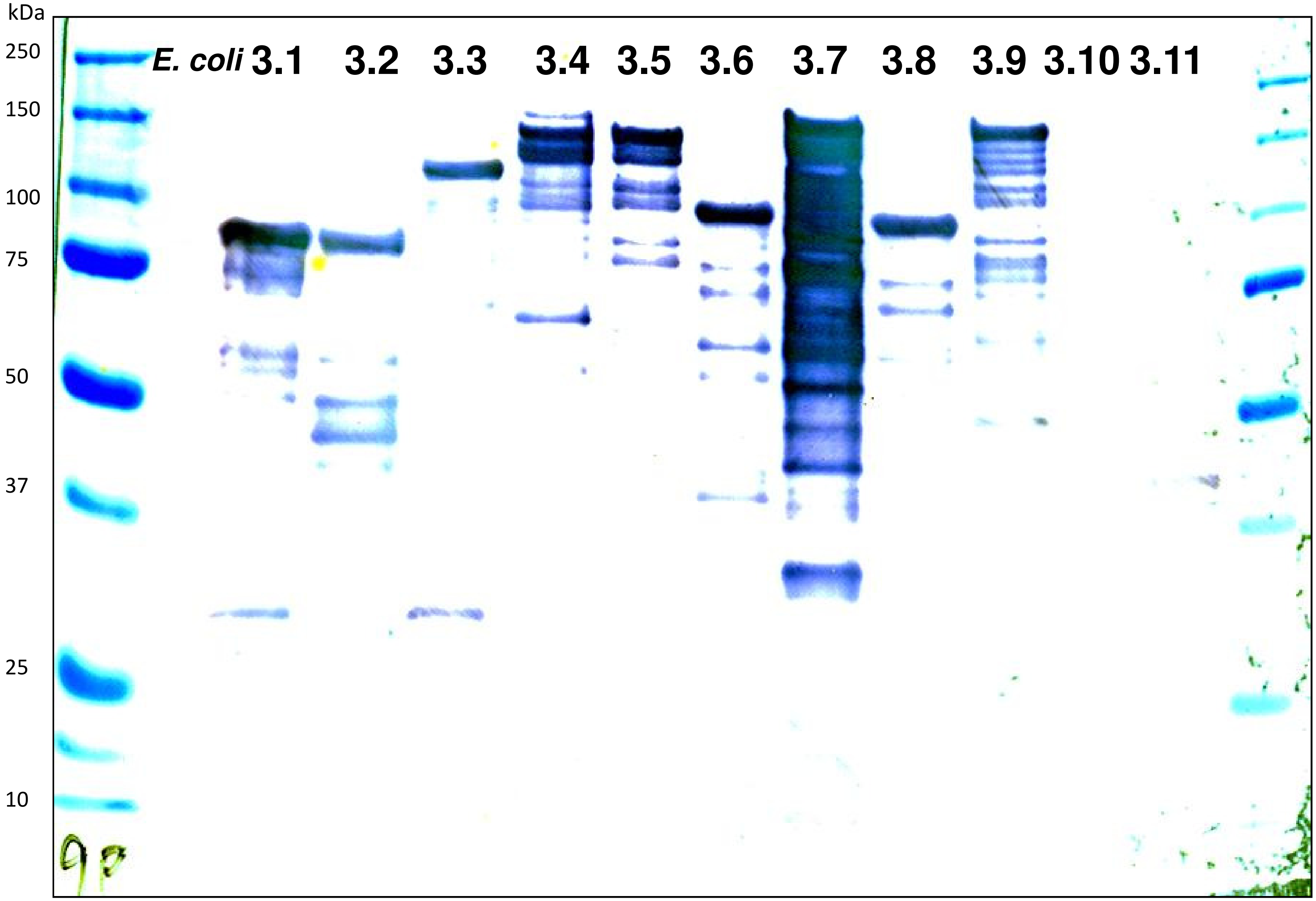

Figure S6

Immunoblot using α-rPvMSP3.7 Ab **after** affinity purification with rPvMSP3.4, rPvMSP3.5 and rPvMSP3.9 columns

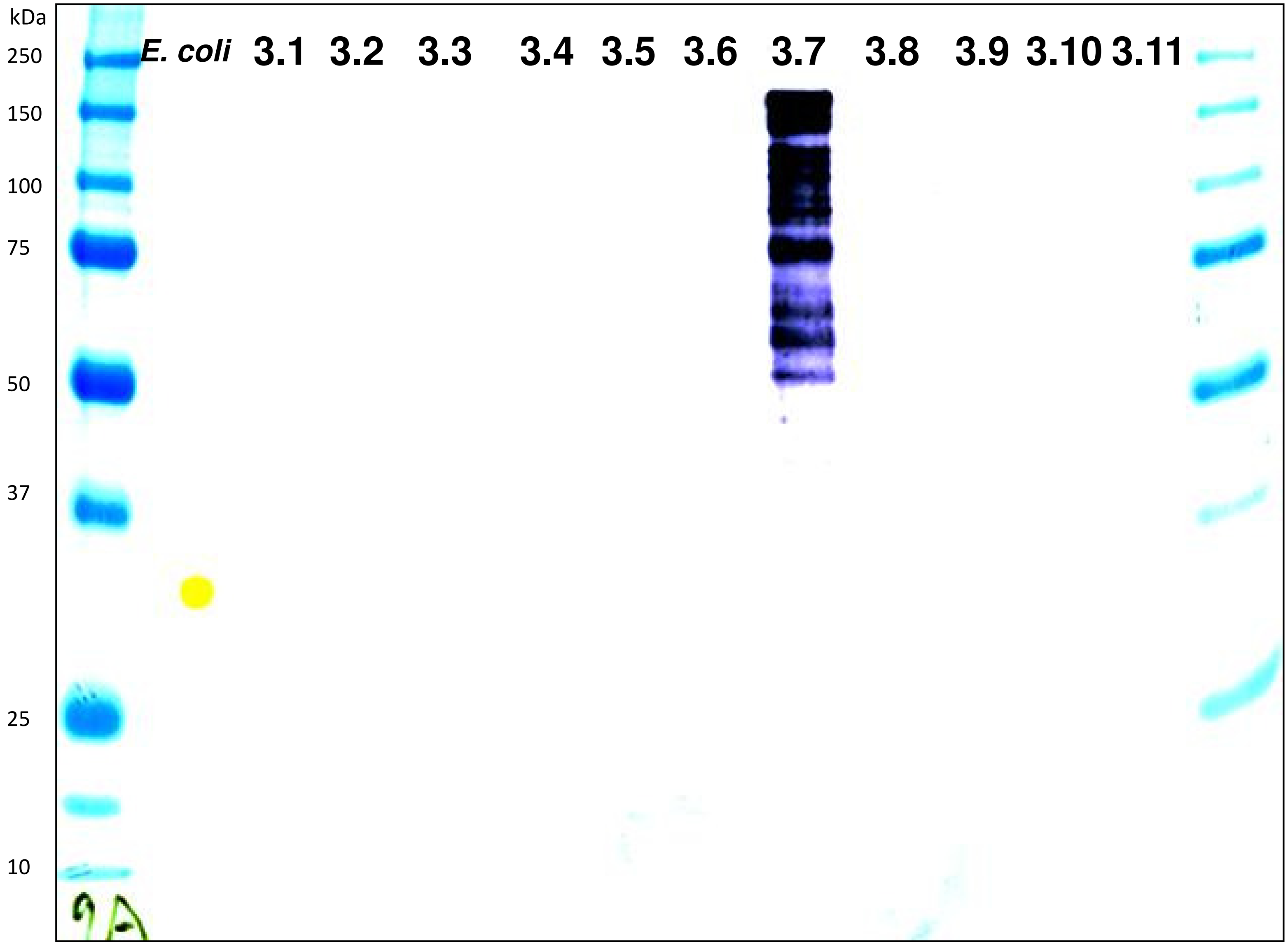

Figure S6

Immunoblot using α-rPvMSP3.8 Ab **before** affinity purification with rPvMSP3.2, rPvMSP3.3 and rPvMSP3.6 columns

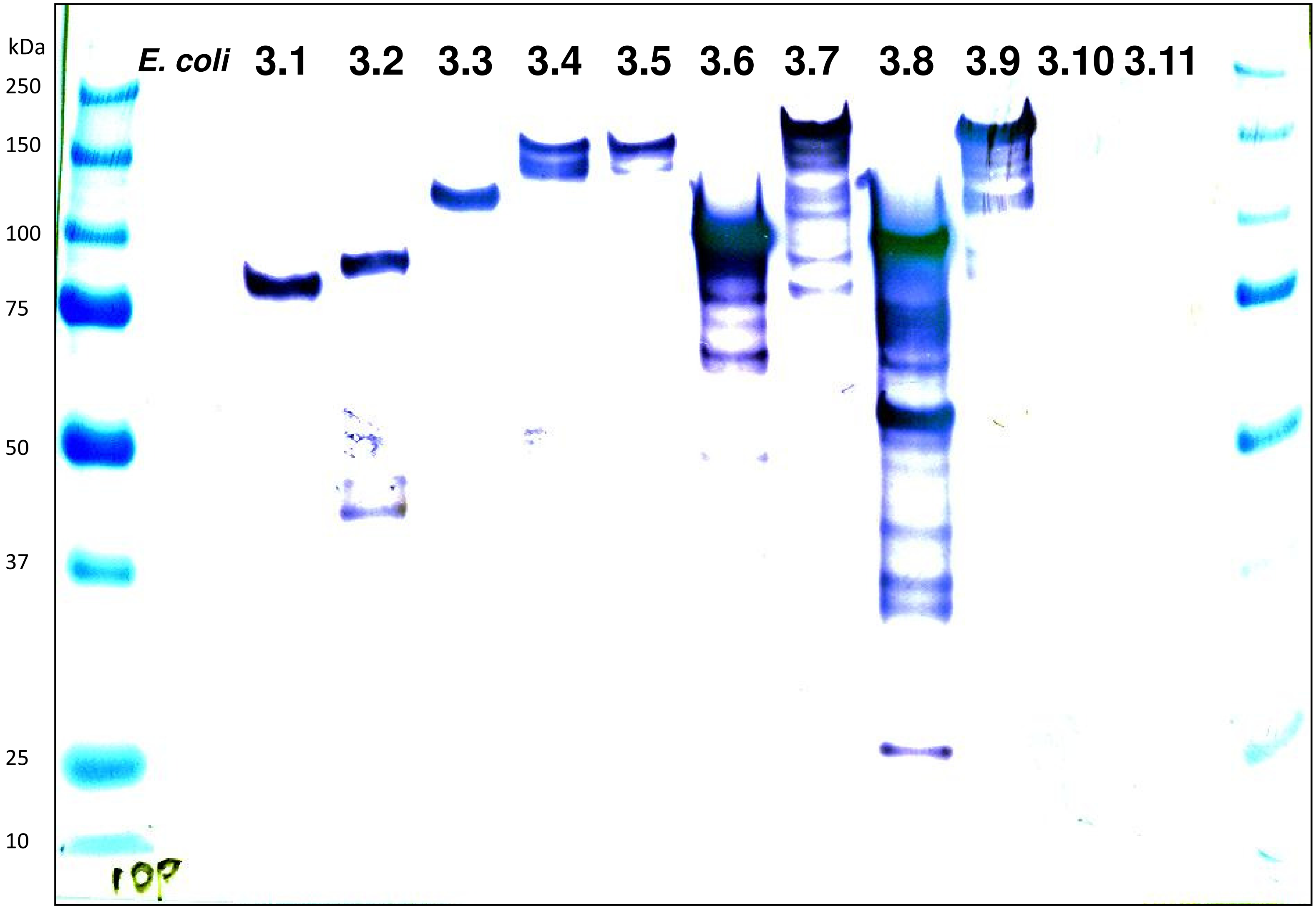

Figure S6

Immunoblot using α-rPvMSP3.8 Ab **after** affinity purification with rPvMSP3.2, rPvMSP3.3 and rPvMSP3.6 columns

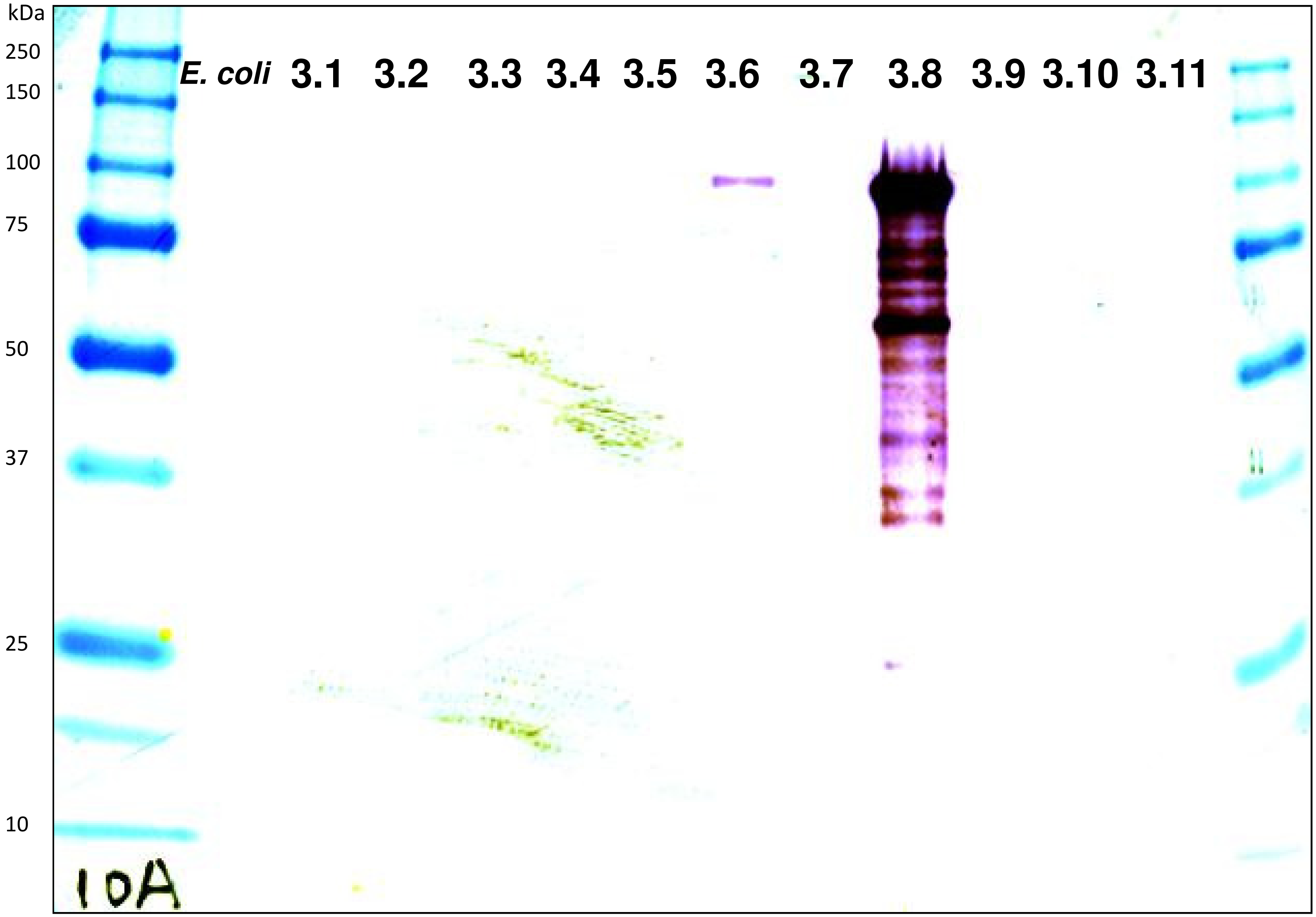

Figure S6

Immunoblot using α-rPvMSP3.8 Ab **after** secondary affinity purification with rPvMSP3.6 antigen columns

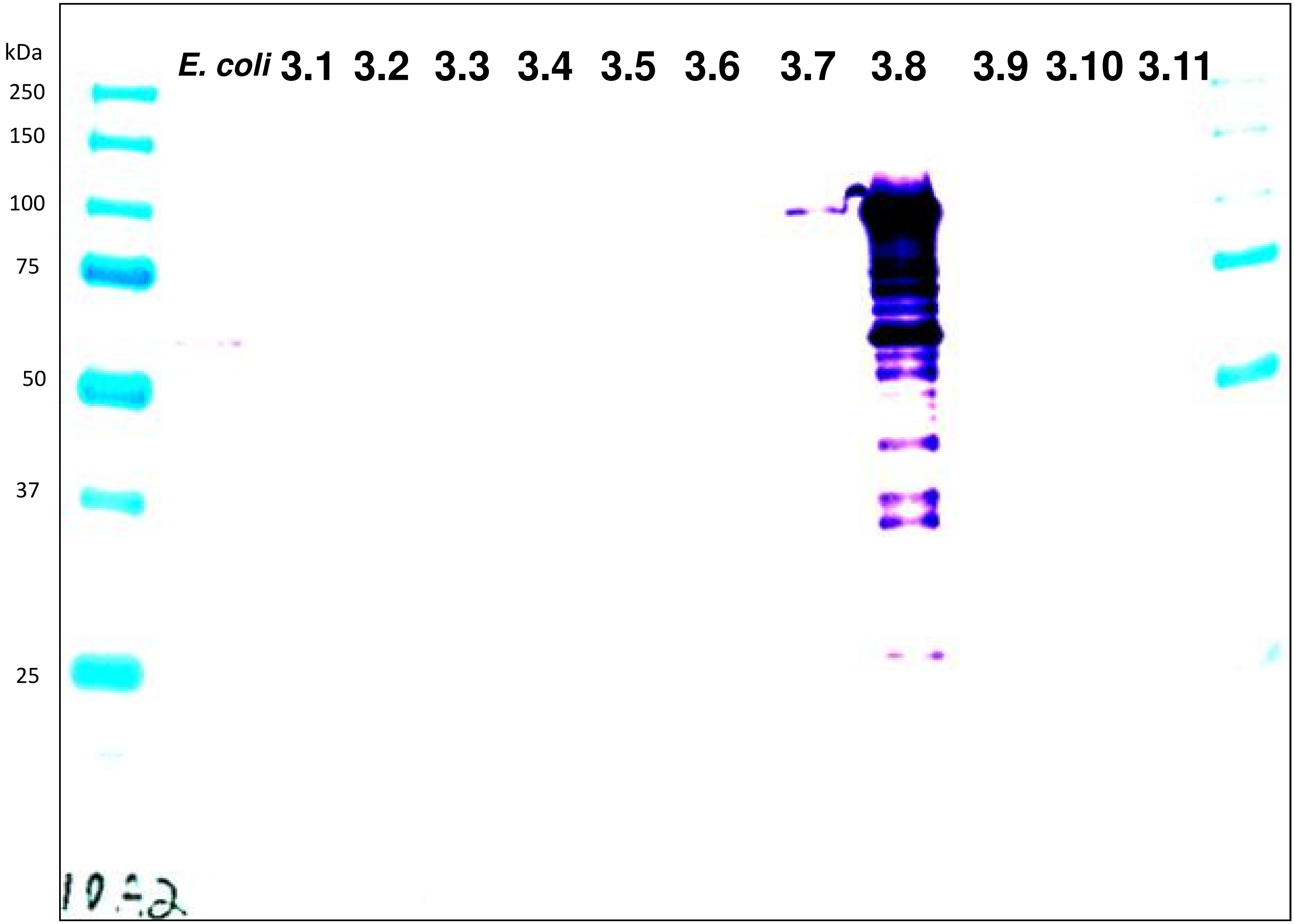

Figure S6

Immunoblot using α-rPvMSP3.9 Ab **before** affinity purification with rPvMSP3.4, rPvMSP3.5 and rPvMSP3.7 columns

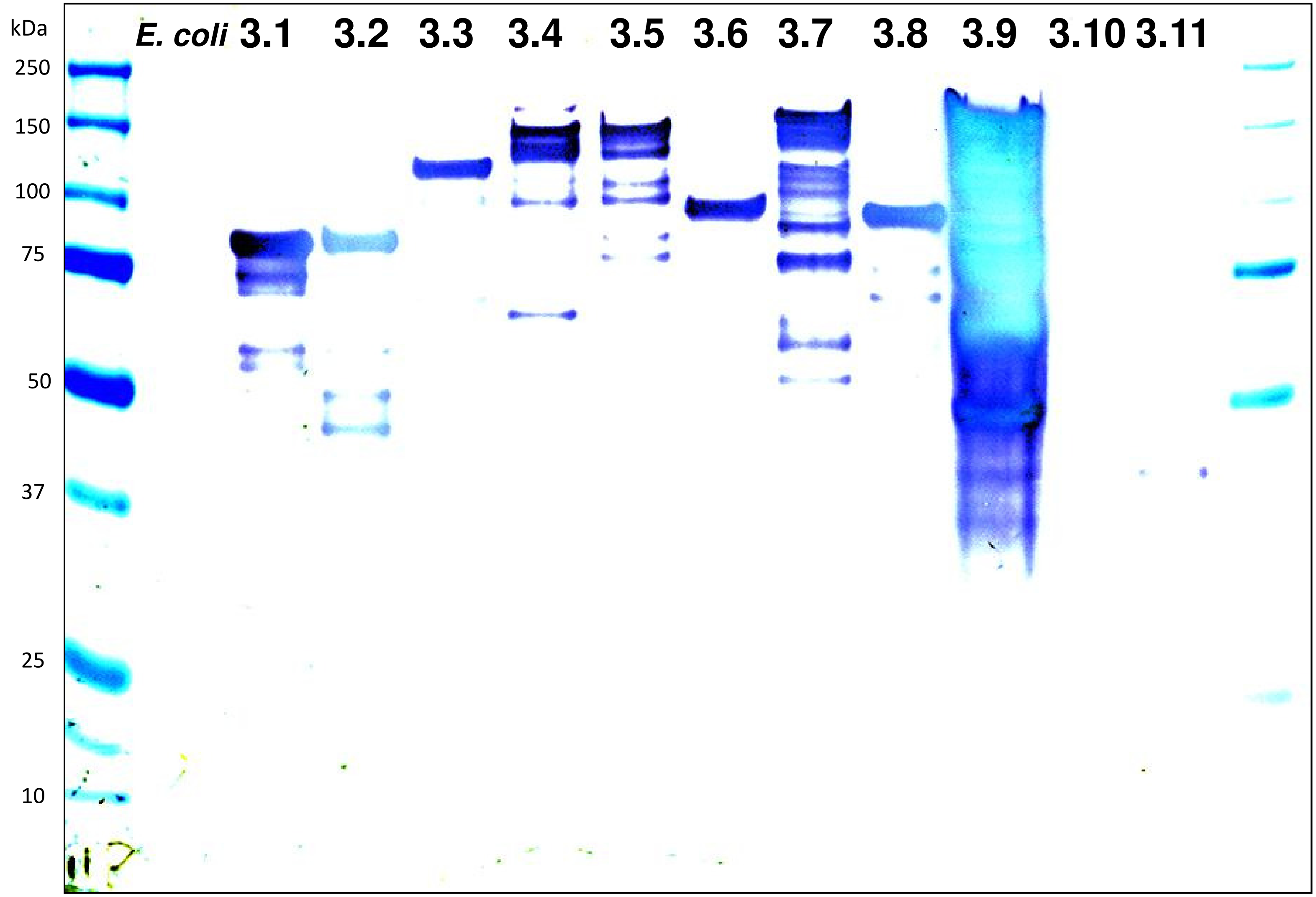

Figure S6

Immunoblot using α-rPvMSP3.9 Ab **after** affinity purification with rPvMSP3.4, rPvMSP3.5 and rPvMSP3.7 columns

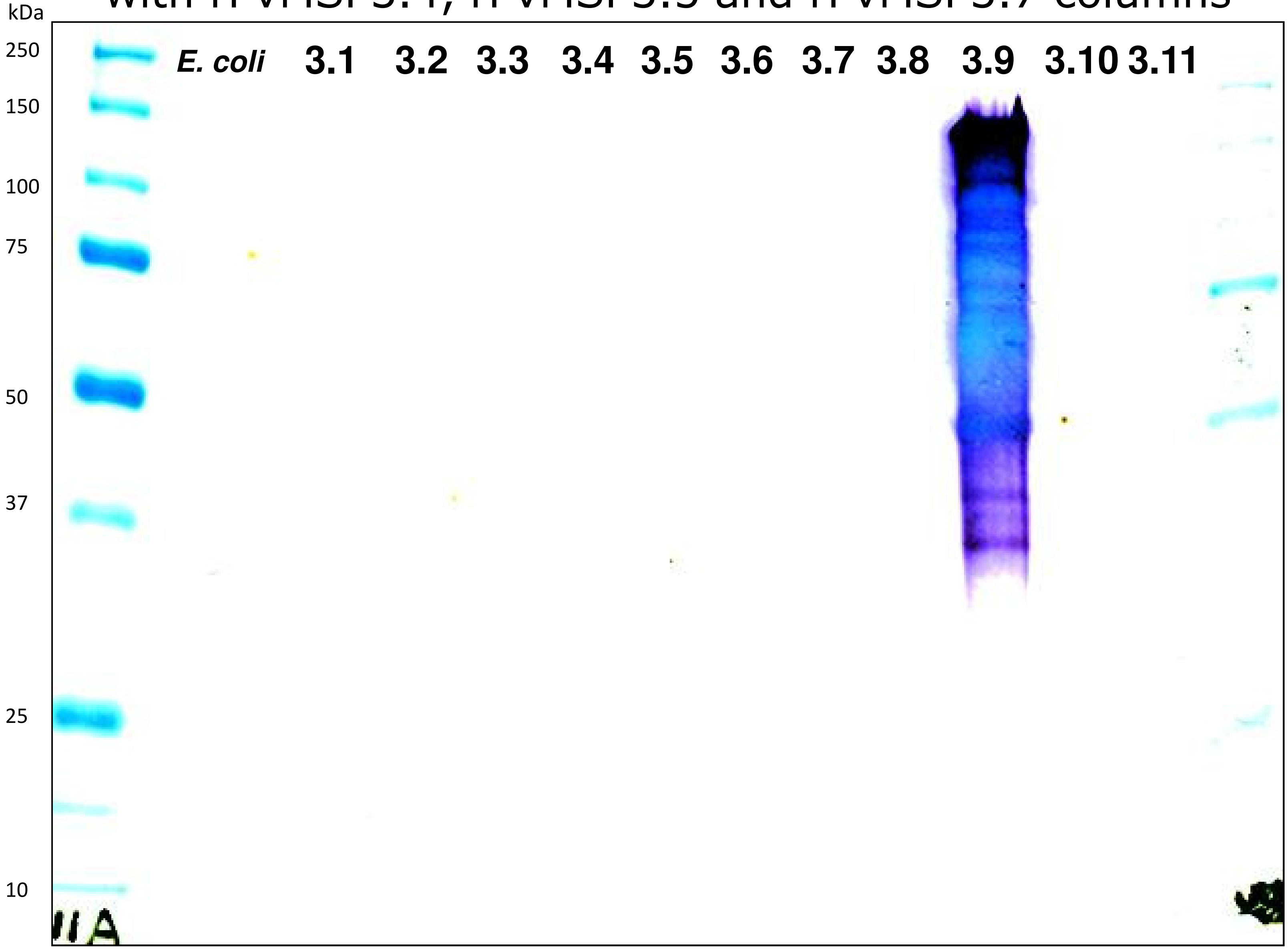

Figure S6

Immunoblot using α-rPvMSP3.10 Ab **before** affinity purification with rPvMSP3.1, rPvMSP3.8 and rPvMSP3.9 columns

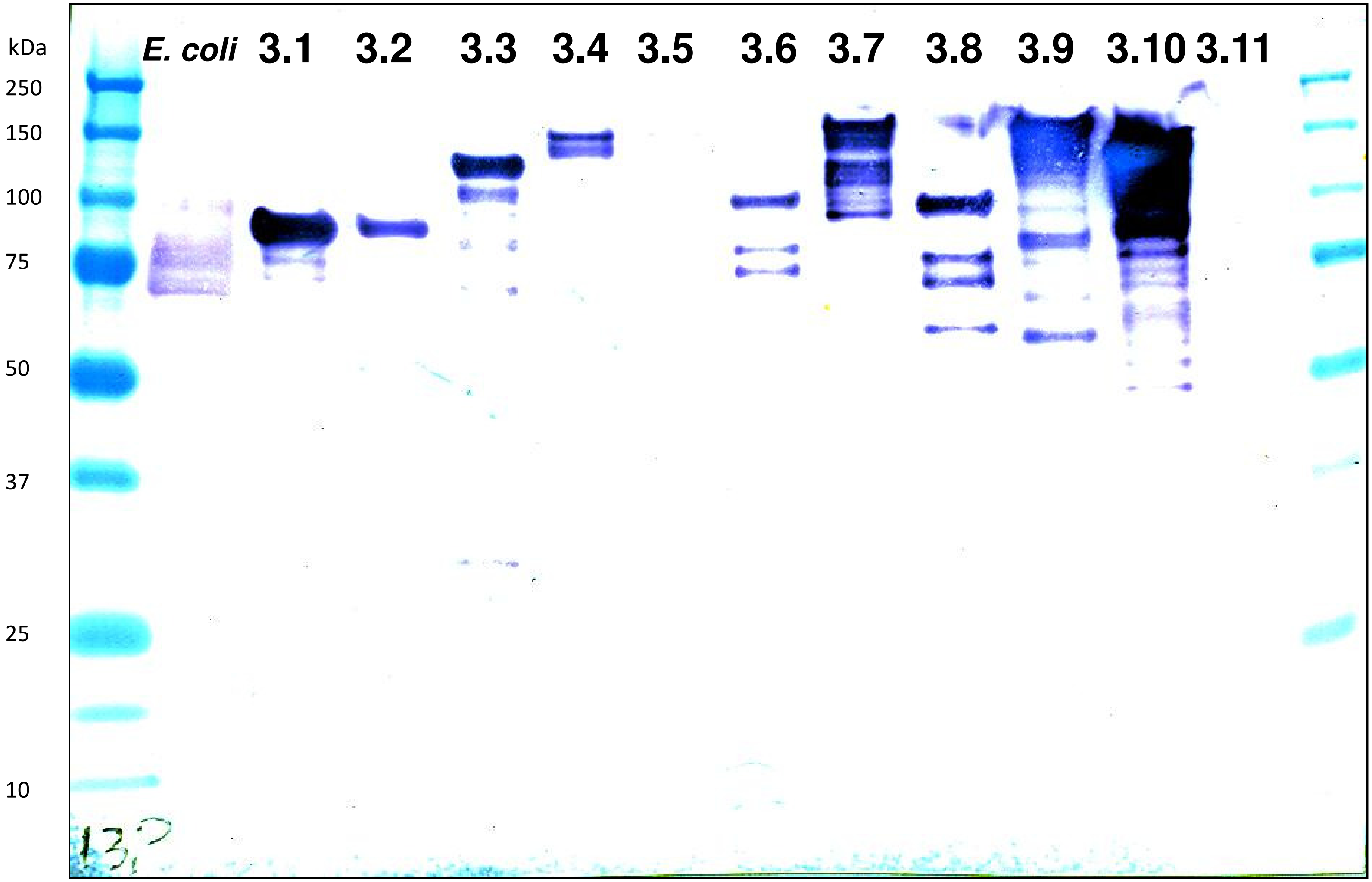

Figure S6

Immunoblot using α-rPvMSP3.10 Ab **after** affinity purification with rPvMSP3.1, rPvMSP3.8 and rPvMSP3.9 columns

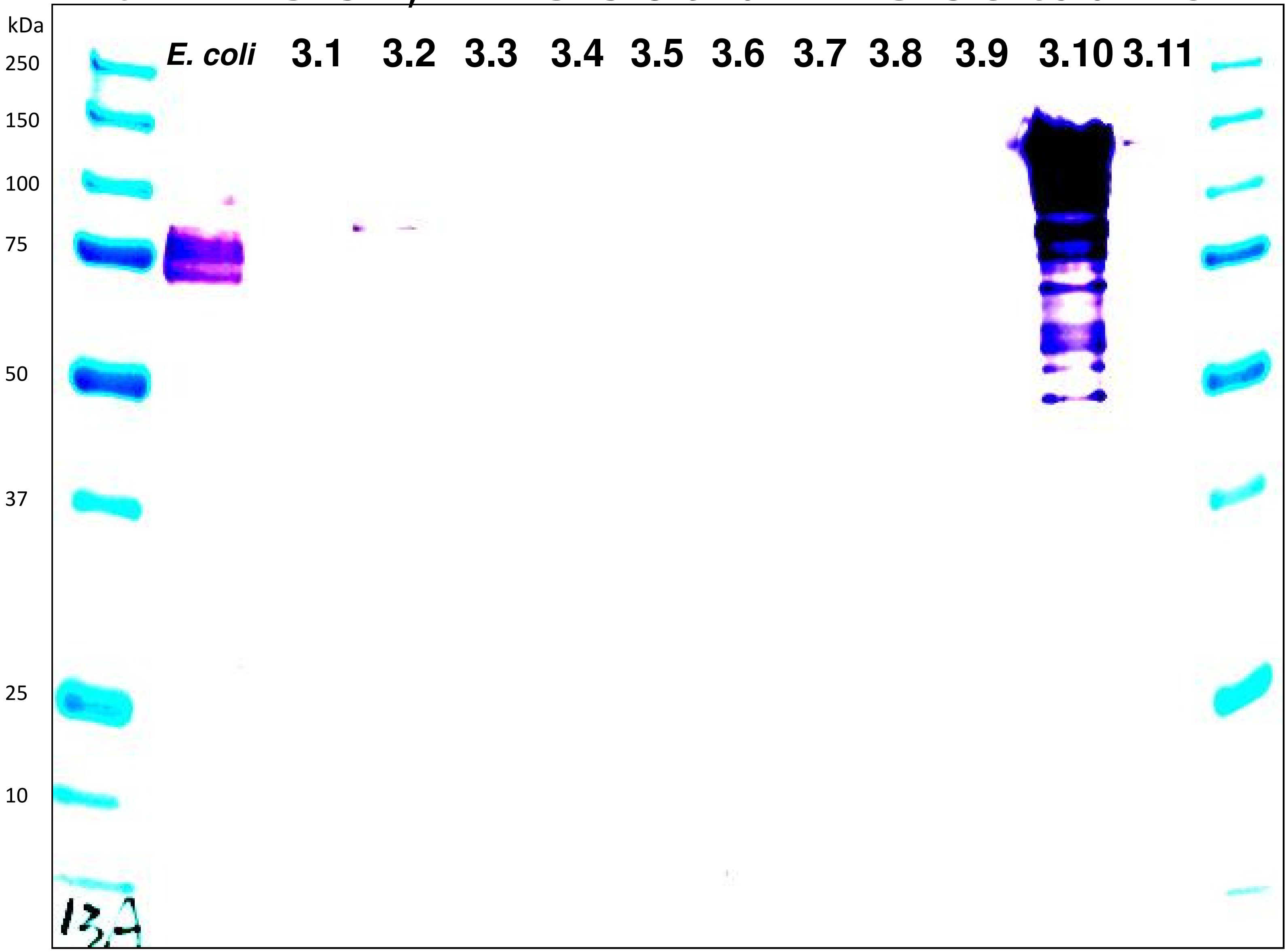

Figure S6

Immunoblot using α-rPvMSP3.10 Ab **after** secondary affinity purification with rPvMSP3.2, rPvMSP3.3 and rPvMSP3.4 antigen columns

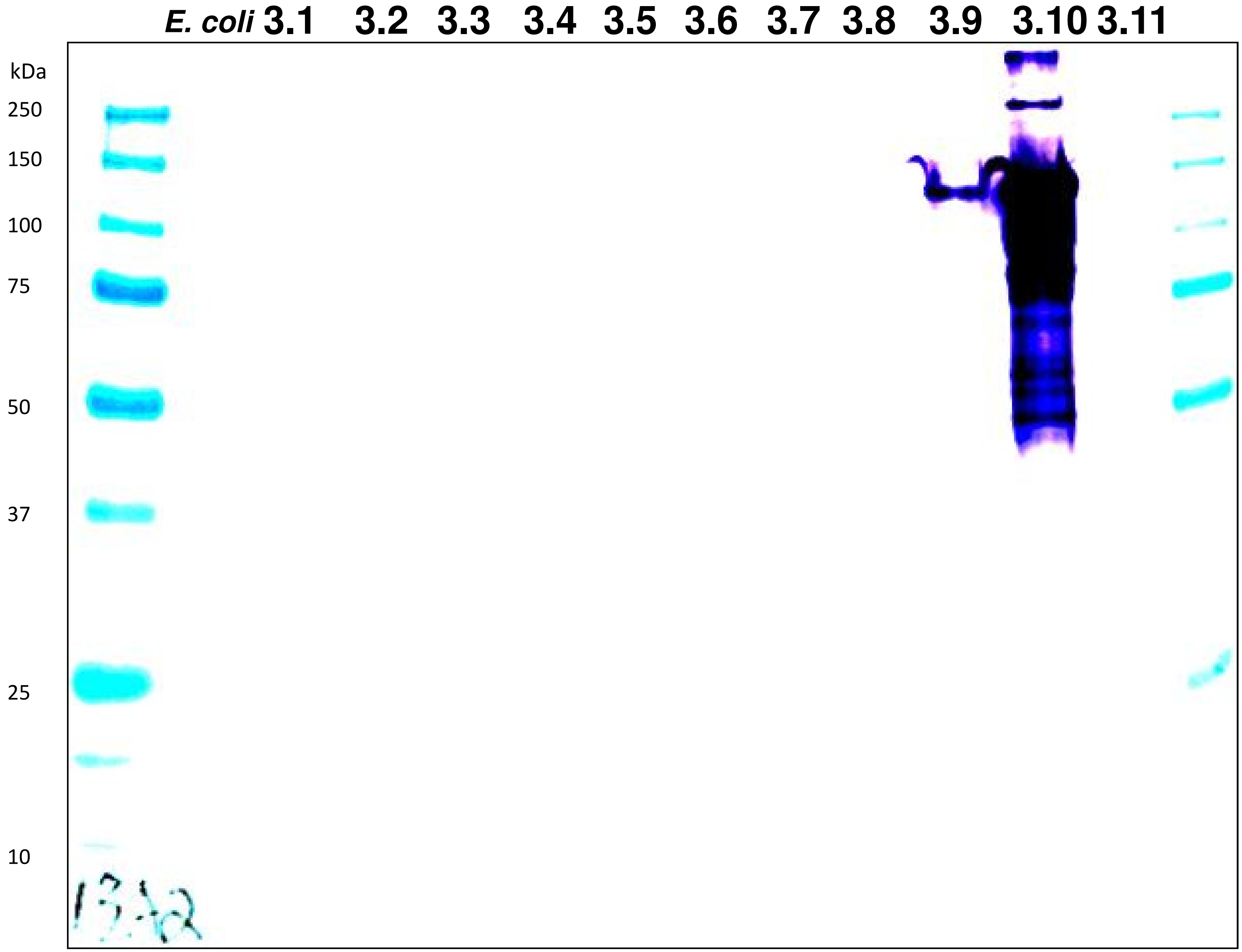

Figure S6

Immunoblot using α-rPvMSP3.11 Ab **before** affinity purification with rPvMSP3.2, rPvMSP3.3 and rPvMSP3.6 columns

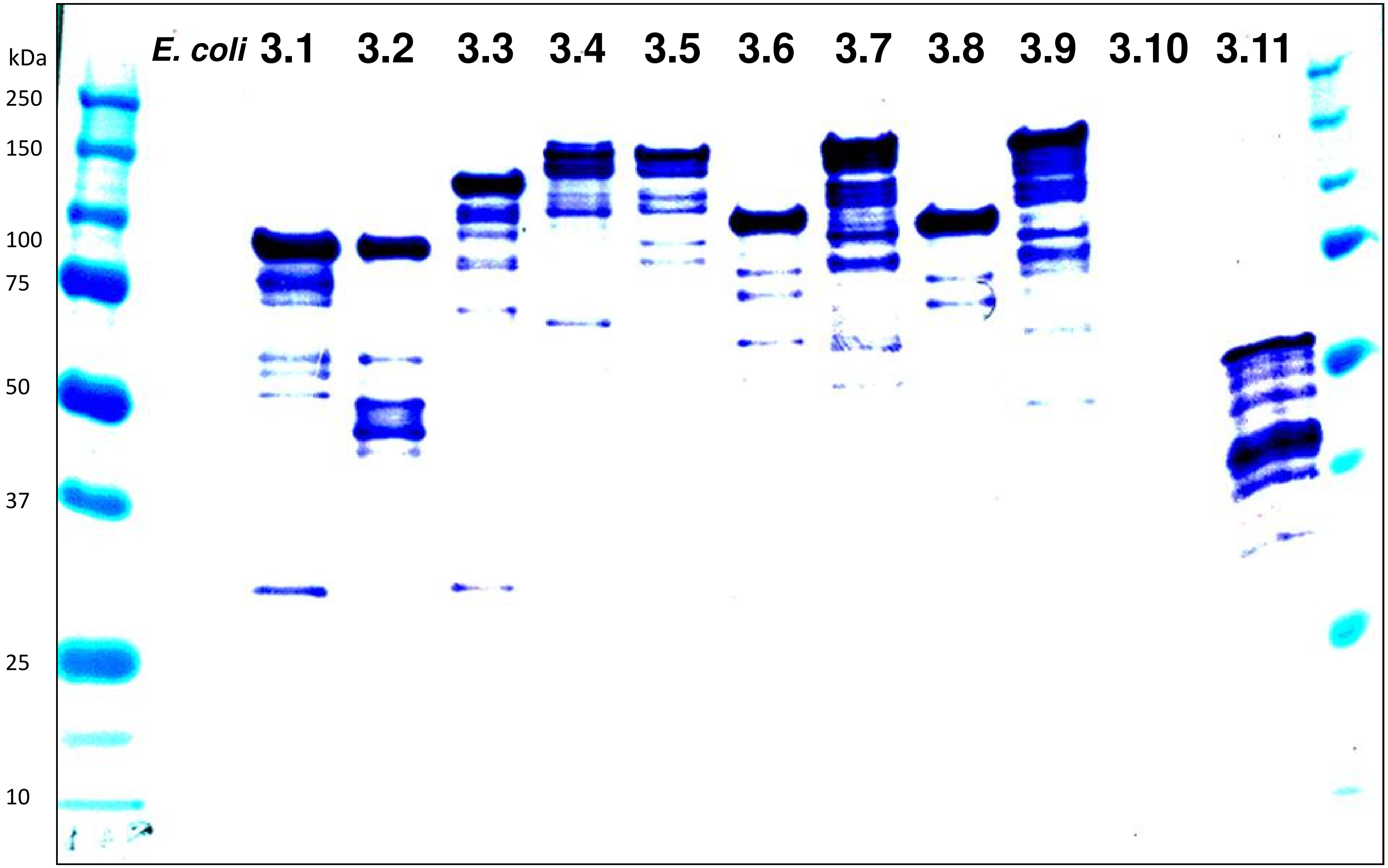

Figure S6

Immunoblot using α-rPvMSP3.11 Ab **after** affinity purification with rPvMSP3.2, rPvMSP3.3 and rPvMSP3.8 columns

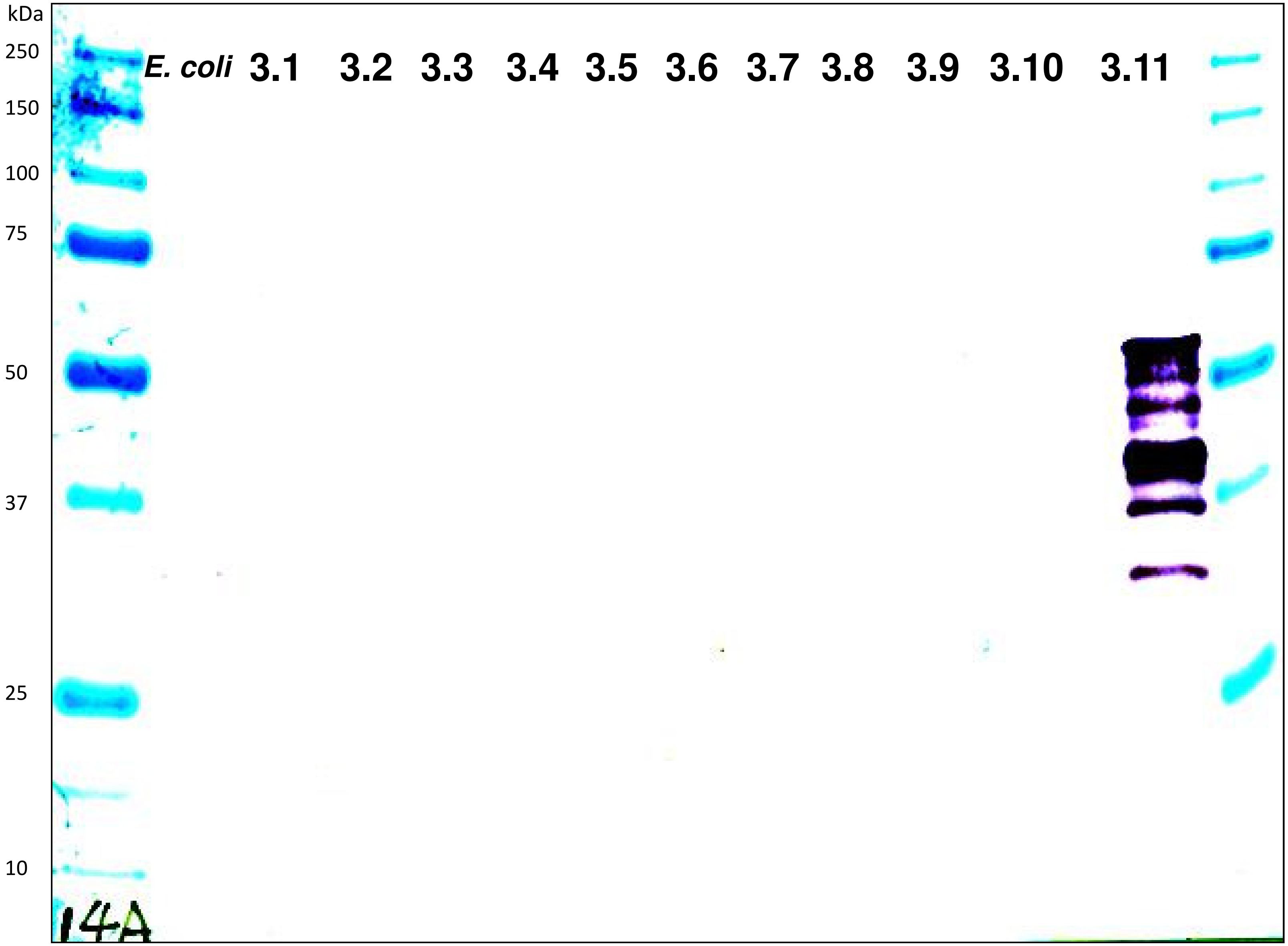

Supplement: Figure S6 — Cross-reactive antibodies present in the rPvMSP3 antisera were removed by serial passage through affinity columns. (PDF) [file pone.0063888.s006.pdf]
