# Supplementary material for: Plasmodium vivax Merozoite Surface Protein-3 (PvMSP3): Expression of an 11 Member Multigene Family in Blood-Stage Parasites
Source: PLoS One. 2013 May 23;8(5):e63888. doi: 10.1371/journal.pone.0063888 (PMC3662707; doi:10.1371/journal.pone.0063888)

Figure S7

Detection of PvMSP3 homologs PcyMSP3 and PkMSP3 with rabbit antiserum against specific rPvMSP3s. Same volume (10ul) of *P. cynomolgi* and *P. knowlesi* parasite extracts representing schizont stage from the Berok and H strain respectively were separated by 7.5% SDS-PAGE, transferred to nitrocellulose membranes and probed with primary antisera at 1:5,000 dilution. All membranes were exposed for the same length of time for chemiluminesence detection.

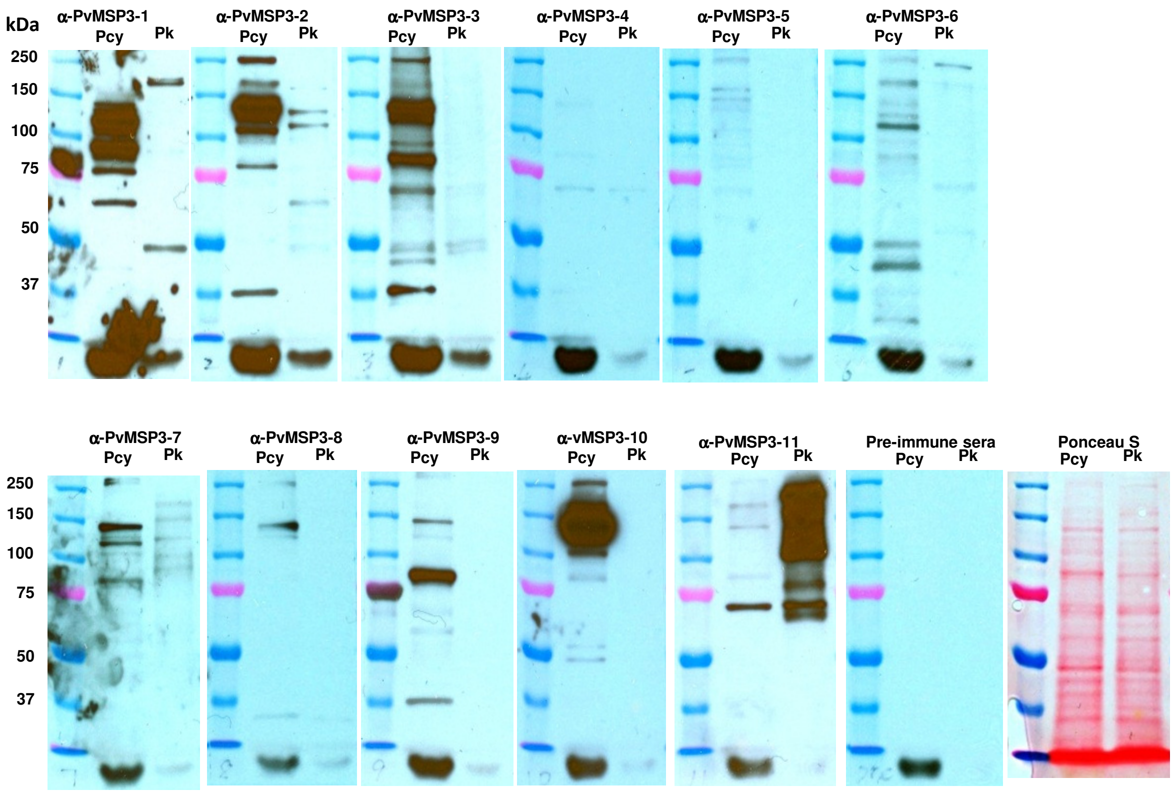

Supplement: Figure S7 — Detection of PvMSP3 homologs PcyMSP3 and PkMSP3 with rabbit antiserum against specific rPvMSP3s. Same volume (10 ul) of P. cynomolgi and P. knowlesi parasite extracts representing schizont stage from the Berok and H strain respectively were separated by 7.5% SDS-PAGE, transferred to nitrocellulose membranes and probed with primary antisera at 1∶5,000 dilution. All membranes were exposed for the same length of time for chemiluminesence detection. (PDF) [file pone.0063888.s007.pdf]
