# Supplementary material for: Plasmodium vivax Merozoite Surface Protein-3 (PvMSP3): Expression of an 11 Member Multigene Family in Blood-Stage Parasites
Source: PLoS One. 2013 May 23;8(5):e63888. doi: 10.1371/journal.pone.0063888 (PMC3662707; doi:10.1371/journal.pone.0063888)
Supplement: Table S1 — Primers used for gene-specific PCR, quantitative real-time RT-PCR, and recombinant protein expression. (PDF) [file pone.0063888.s008.pdf]

Table S1. Primers used for gene-specific PCR, quantitative real-time RT-PCR, and recombinant protein expression

Gene	Primer	Sequence (5' → 3') Real-time PCR	Sequence (5' → 3') Gene specific PCR	Sequence (5' → 3') Protein expression
*pvmsp*3.1	Sense	AGACCAAAACGAATTGCTACACAA	CACAAATAGTATGGCATATCTC	CACCATGGCACAAATTAAAAAAGCTAACGAAG
	Antisense	CTGATCCCCCTATTTCCTTGACT	TTCCGTGCTTACTTTCTCTA	CTACTCGAGCTTCAATAATTCAAAGGCTG
	Taqman probe	TCGGAAGAAACAGATTTTCAAACGCAAGGA		
*pvmsp*3.2	Sense	AGGCAAGTGATAACGTATTCGATAAA	AAAATCAGAAGCACAGGTGC	CACCATGGTTGAAAAAGCTAACGAAGCA
	Antisense	TCCTCTGCAGCATCGTTTTCT	CTTGCCATACTAGCTGATGC	CTACTCGAGTTCTGCTTCACTTGTTGCTT
	Taqman probe	AGGTGGCACAGGAAGTCGAAGATCGG		
*pvmsp*3.3	Sense	AGTTCAAAGATTCAAATACTGCGAAA	AGCAACTTTGTGGCTTAGCC	CACCATGGCAGCTGAACAAAAAGTGCATGC
	Antisense	TGTCTTCCCCAACATCGCTAA	CGGAGATCCCTTTTGTCCTT	CTACTCGAGCTTTTTTGTTCTAATCAATG
	Taqman probe	CGGGAGGAGTGGAATTTTTGGAAACTCTG		
*pvmsp*3.4	Sense	AGGCTACAATGTGGTCGATAATGA	TATTCACCAAAATGTTGTGAGC	CACCATGGCAAGTAAAGAAGACATAAAAAC
	Antisense	TCGCTC GATGCTGAATCGT	TCAATTCTGCTTCATCTGTC	CTACTCGAGTTCATCACTCTTTTCTTGGG
	Taqman probe	CAGGTGCTAAACGAAGTTGACGAACAGGC		
*pvmsp*3.5	Sense	GCAGAAGACGGTGATGAGATAGAC	GAAGCAATTTTGCGGTATCC	CACCATGGCAGAAGACTTAAAAAGACAAATAGG
	Antisense	TCTTCATTATCATCATTACCTTGTTCCT	TGCTAATTGTGCTTGATCAA	CTACTCGAGTTCTAACTCGGTTATGCTGC
	Taqman probe	ACGGAAGCCATATCGAATTGCCACAAA		
*pvmsp*3.6	Sense	ACGCAGACAAGCAGTTTGGA	GGGACAAGATAAAGAAAAGGTGG C	CACCATGGCACAACAAAAATCGGATGAAAC
	Antisense	AACTAAGGACGTCATCGTTTTTACG	CCCAATGCTGTATTCGCTGT	CTACTCGAGAGCTGATGTTGCTGCCTTTT
	Taqman probe	CGACGCGTACAACGACATTAAGAAGGTAAC AGA		
*pvmsp*3.7	Sense	AATTAAAAATAGCCGAACAGGAATTG	ACAACAAGAAGAAGCAGTCGATTT	CACCATGGCAGCACAACAAGAAGAAGCAGT
	Antisense	CCGCAAGGGCTTTCGTT	CATTGCCTGTTGCTTATTTG	CTACTCGAGTTCACTCTTTTCTTGGGATT
	Taqman probe	AGCTAAGGGAGCCACGTCGATGGAA		
*pvmsp*3.8	Sense	ACCAAAGCGAATTGCTACACAA	ACGCAGGGGCAAAATAACGAAGT	CACCATGGCACAGGGGCAAAATAACGAAGT
	Antisense	TGATTCCTCCACCACTTCGTT	GCACCTGCTTGTTTGGGCTT	CTACTCGAGAGCTGATGTTGCTGCCTTTT
	Taqman probe	CGGATGAAGGCTCACTCCAGACGC		
*pvmsp*3.9	Sense	GATAACTCTCCCCATAGTGACACTGA	CACGTTGCTACAGCAGAAAG	CACCATGGCATCAGAAGATGAAAAAGAGGCAGAA A
	Antisense	AGGAGCGCTTGGGTGTTG	CTTGTCGACGTGCATCCATT	CTACTCGAGAAGTTTTTTCTTTAAGGCTG
	Taqman probe	AAATCAAAGTGTGAAACCCCCGGAACA		
*pvmsp*3.10	Sense	TGGCGCCGAGGTTATAGG	ATGAAACACACCCGCAGCGT	CTAGCCATGGATCAAGTGACCGAGAAG
	Antisense	TCCTTAAATGGTGTCTGCTGGAA	AGGCTTTGTCGCATCCTGTG	ATGCTCGAGTAGGTGATTCATATCGGA
	Taqman probe	CCCCCAACAATGAAGCCCCGA		
*pvmsp*3.11	Sense	AAGGAAGCAACGCGAAAGC	ATGAGGCAATTCGTCAGAAT	CACCATGGCACTAAACGTTAACAAAACGAA
	Antisense	GGATGTGCTGGTTCGTTGTCT	ACTTCTTTGATGCTGTCCCC	CTACTCGAGATCATCCTCGTCTTCGTCAT
	Taqman probe	AGGCCGAAGCGCACGAATTTTTG		
pvSeryl-tRNA	Sense	CATCCCATATAGAATCGTCAGCAT		
	Antisense	GGAAGTAGCCCTCCAAATCAAA		
	Taqman probe	CCCTCAACAACGCCGCTGCTGT		
