# Supplementary material for: Plasmodium vivax Merozoite Surface Protein-3 (PvMSP3): Expression of an 11 Member Multigene Family in Blood-Stage Parasites
Source: PLoS One. 2013 May 23;8(5):e63888. doi: 10.1371/journal.pone.0063888 (PMC3662707; doi:10.1371/journal.pone.0063888)
Supplement: Table S2 — Gene accession numbers or identification numbers used for analysis. (PDF) [file pone.0063888.s009.pdf]

Table S2. Gene accession numbers or identification numbers used for analysis

Gene	Accession number/Gene ID	Strain	Source
*pvmsp*3.1	PVX_097670	Sal 1	PlasmoDB - *Plasmodium* Genomics Resource
*pvmsp*3.2	PVX_097675	Sal 1	PlasmoDB - *Plasmodium* Genomics Resource
*pvmsp*3.3	PVX_097680	Sal 1	PlasmoDB - *Plasmodium* Genomics Resource
*pvmsp*3.4	PVX_097685	Sal 1	PlasmoDB - *Plasmodium* Genomics Resource
*pvmsp*3.5	PVX_097690	Sal 1	PlasmoDB - *Plasmodium* Genomics Resource
*pvmsp*3.6	PVX_097695	Sal 1	PlasmoDB - *Plasmodium* Genomics Resource
*pvmsp*3.7	PVX_097700	Sal 1	PlasmoDB - *Plasmodium* Genomics Resource
*pvmsp*3.8	PVX_097705	Sal 1	PlasmoDB - *Plasmodium* Genomics Resource
*pvmsp*3.9	PVX_097710	Sal 1	PlasmoDB - *Plasmodium* Genomics Resource
*pvmsp*3.10	PVX_097720	Sal 1	PlasmoDB - *Plasmodium* Genomics Resource
*pvmsp*3.11	PVX_097725	Sal 1	PlasmoDB - *Plasmodium* Genomics Resource
pvSeryl-tRNA	PVX_000545	Sal 1	PlasmoDB - *Plasmodium* Genomics Resource
*pcymsp*3.10	KC109789	Berok	Galinski *et al*, unpublished
pkmsp3_{140}	PKH_145630	H	PlasmoDB - *Plasmodium* Genomics Resource, {David, 1985 #160}
pkmsp3_{172}	PKH_103020	H	PlasmoDB - *Plasmodium* Genomics Resource, Barnwell *et al* unpublished
*pfmsp*3.1	L07944.1	FC27	NCBI
*pfmsp*3.1	U08852.1	CSL2	NCBI
