# Supplementary material for: Plasmodium vivax Merozoite Surface Protein-3 (PvMSP3): Expression of an 11 Member Multigene Family in Blood-Stage Parasites
Source: PLoS One. 2013 May 23;8(5):e63888. doi: 10.1371/journal.pone.0063888 (PMC3662707; doi:10.1371/journal.pone.0063888)
Supplement: Table S3 — Raw data of qRT-PCR detection of transcript level of PvMSP3 family members with TaqMan probes. (PDF) [file pone.0063888.s010.pdf]

**Table S3.** Raw data of qRT-PCR detection of transcript level of PvMSP3 family members with TaqMan probes

| Well | Ct | Identifier | $2^{-\Delta\Delta Ct}$ | Well | Ct | Identifier | $2^{-\Delta\Delta Ct}$ | Average $2^{-\Delta\Delta Ct}$ | STDEV |
|---|---|---|---|---|---|---|---|---|---|
| B1 | 26.8 | Pvseryl-tRNA synthetase Primers / gDNA | | C1 | 26.9 | Pvseryl-tRNA synthetase Primers / gDNA | | | |
| B2 | 27.9 | PvMSP3.1 Primers / gDNA | | C2 | 27.9 | PvMSP3.1 Primers / gDNA | | | |
| B3 | 25.6 | PvMSP3.2 Primers / gDNA | | C3 | 25.4 | PvMSP3.2 Primers / gDNA | | | |
| B4 | 26.4 | PvMSP3.3 Primers / gDNA | | C4 | 26.6 | PvMSP3.3 Primers / gDNA | | | |
| B5 | 26.8 | PvMSP3.4 Primers / gDNA | | C5 | 26.8 | PvMSP3.4 Primers / gDNA | | | |
| B6 | 27.8 | PvMSP3.5 Primers / gDNA | | C6 | 28 | PvMSP3.5 Primers / gDNA | | | |
| B7 | 24.9 | PvMSP3.6 Primers / gDNA | | C7 | 24.4 | PvMSP3.6 Primers / gDNA | | | |
| B8 | 26.3 | PvMSP3.7 Primers / gDNA | | C8 | 26.3 | PvMSP3.7 Primers / gDNA | | | |
| B9 | 24.8 | PvMSP3.8 Primers / gDNA | | C9 | 25.1 | PvMSP3.8 Primers / gDNA | | | |
| B10 | 27.9 | PvMSP3.9 Primers / gDNA | | C10 | 28.3 | PvMSP3.9 Primers / gDNA | | | |
| B11 | 25.7 | PvMSP3.10 Primers / gDNA | | C11 | 25.8 | PvMSP3.10 Primers / gDNA | | | |
| B12 | 25.8 | PvMSP3.11 Primers / gDNA | | C12 | 26.3 | PvMSP3.11 Primers / gDNA | | | |
| D1 | 32 | Pvseryl-tRNA synthetase Primers / Trophs cDNA | | E1 | 31.6 | Pvseryl-tRNA synthetase Primers / Trophs cDNA | | | |
| D2 | 28.2 | PvMSP3.1 Primers / Trophs cDNA | 29.86 | E2 | 28.2 | PvMSP3.1 Primers / Trophs cDNA | 21.11 | 25.48 | 6.18 |
| D3 | 30 | PvMSP3.2 Primers / Trophs cDNA | 1.74 | E3 | 29.3 | PvMSP3.2 Primers / Trophs cDNA | 1.74 | 1.74 | 0.00 |
| D4 | 30.8 | PvMSP3.3 Primers / Trophs cDNA | 1.74 | E4 | 30.4 | PvMSP3.3 Primers / Trophs cDNA | 1.87 | 1.80 | 0.09 |
| D5 | 29.6 | PvMSP3.4 Primers / Trophs cDNA | 5.28 | E5 | 29.6 | PvMSP3.4 Primers / Trophs cDNA | 3.73 | 4.51 | 1.09 |
| D6 | 32.5 | PvMSP3.5 Primers / Trophs cDNA | 1.41 | E6 | 32.1 | PvMSP3.5 Primers / Trophs cDNA | 1.52 | 1.46 | 0.07 |
| D7 | 29.3 | PvMSP3.6 Primers / Trophs cDNA | 1.74 | E7 | 29.4 | PvMSP3.6 Primers / Trophs cDNA | 0.81 | 1.28 | 0.66 |
| D8 | 29.2 | PvMSP3.7 Primers / Trophs cDNA | 4.92 | E8 | 29.8 | PvMSP3.7 Primers / Trophs cDNA | 2.30 | 3.61 | 1.86 |
| D9 | 29.4 | PvMSP3.8 Primers / Trophs cDNA | 1.52 | E9 | 29.1 | PvMSP3.8 Primers / Trophs cDNA | 1.62 | 1.57 | 0.08 |
| D10 | 29.9 | PvMSP3.9 Primers / Trophs cDNA | 9.19 | E10 | 29.6 | PvMSP3.9 Primers / Trophs cDNA | 10.56 | 9.87 | 0.97 |
| D11 | 29.5 | PvMSP3.10 Primers / Trophs cDNA | 2.64 | E11 | 29.8 | PvMSP3.10 Primers / Trophs cDNA | 1.62 | 2.13 | 0.72 |
| D12 | 31.6 | PvMSP3.11 Primers / Trophs cDNA | 0.66 | E12 | 31.6 | PvMSP3.11 Primers / Trophs cDNA | 0.66 | 0.66 | 0.00 |
| F1 | 33.1 | Pvseryl-tRNA synthetase Primers / SZ cDNA | | G1 | 32.6 | Pvseryl-tRNA synthetase Primers / SZ cDNA | | | |
| F2 | 31.6 | PvMSP3.1 Primers / SZ cDNA | 6.06 | G2 | 31.2 | PvMSP3.1 Primers / SZ cDNA | 5.28 | 5.67 | 0.55 |
| F3 | 27.9 | PvMSP3.2 Primers / SZ cDNA | 16.00 | G3 | 28.2 | PvMSP3.2 Primers / SZ cDNA | 7.46 | 11.73 | 6.04 |
| F4 | 31.9 | PvMSP3.3 Primers / SZ cDNA | 1.74 | G4 | 31.9 | PvMSP3.3 Primers / SZ cDNA | 1.32 | 1.53 | 0.30 |
| F5 | 31.4 | PvMSP3.4 Primers / SZ cDNA | 3.25 | G5 | 31.6 | PvMSP3.4 Primers / SZ cDNA | 1.87 | 2.56 | 0.98 |
| F6 | 34 | PvMSP3.5 Primers / SZ cDNA | 1.07 | G6 | 33.7 | PvMSP3.5 Primers / SZ cDNA | 1.00 | 1.04 | 0.05 |
| F7 | 30.1 | PvMSP3.6 Primers / SZ cDNA | 2.14 | G7 | 30.1 | PvMSP3.6 Primers / SZ cDNA | 1.00 | 1.57 | 0.81 |
| F8 | 29.8 | PvMSP3.7 Primers / SZ cDNA | 6.96 | G8 | 29.6 | PvMSP3.7 Primers / SZ cDNA | 5.28 | 6.12 | 1.19 |
| F9 | 31 | PvMSP3.8 Primers / SZ cDNA | 1.07 | G9 | 30.9 | PvMSP3.8 Primers / SZ cDNA | 0.93 | 1.00 | 0.10 |
| F10 | 31.5 | PvMSP3.9 Primers / SZ cDNA | 6.50 | G10 | 31.5 | PvMSP3.9 Primers / SZ cDNA | 5.66 | 6.08 | 0.59 |
| F11 | 30.2 | PvMSP3.10 Primers / SZ cDNA | 3.48 | G11 | 29.9 | PvMSP3.10 Primers / SZ cDNA | 3.03 | 3.26 | 0.32 |
| F12 | 30.7 | PvMSP3.11 Primers / SZ cDNA | 2.64 | G12 | 31.2 | PvMSP3.11 Primers / SZ cDNA | 1.74 | 2.19 | 0.63 |
